# Empagliflozin Prevents Cardiac Arrest-Induced Renal Injury Through BHB-Dependent Mitoribosome Maintenance

**DOI:** 10.3390/ijms27146366

**Published:** 2026-07-17

**Authors:** Kazuhiro Hasegawa, Masanori Tamaki, Sumiyo Yamaguchi, Ikuko Shimizu, Takahiro Kida, Shinji Miyakami, Miho Tada, Chihiro Okinari, Makoto Otsuka, Masanori Minato, Shu Wakino

**Affiliations:** Department of Nephrology, Tokushima University Graduate School of Biomedical Sciences, 3-18-15 Kuramoto-cho, Tokushima 770-8503, Japan

**Keywords:** β-hydroxybutyrate, empagliflozin, cardiac arrest-induced acute kidney injury

## Abstract

Cardiac arrest followed by cardiopulmonary resuscitation (CA/CPR) induces systemic ischemia and frequently results in acute kidney injury (AKI). The ketone body β-hydroxybutyrate (BHB) maintains mitochondrial and peroxisomal homeostasis through activation of the C/EBPβ–Pck1 axis, whereas Pck1 preserves mitoribosome integrity and mtDNA-encoded oxidative phosphorylation (OXPHOS) translation. However, it remains unclear whether this pathway is disrupted during CA/CPR-induced AKI and whether empagliflozin can restore its activity. Male C57BL/6J mice and proximal tubule-specific Pck1 conditional knockout (CKO) mice were subjected to short-duration or standard CA/CPR protocols. Empagliflozin was administered orally for 7 days before CA/CPR induction. Circulating BHB levels, renal expression of C/EBPβ and Pck1, and markers of mitochondrial, peroxisomal, and mitoribosomal abundance and function were evaluated using established methods. CA/CPR markedly reduced circulating BHB levels and suppressed the C/EBPβ–Pck1 signaling axis. These changes were accompanied by depletion of peroxisomal markers, mitochondrial regulators, and mitoribosomal components as well as increased tubular apoptosis and albuminuria. Pck1 CKO mice exhibited severe organelle dysfunction and aggravated renal injury. In contrast, empagliflozin restored BHB levels, preserved C/EBPβ and Pck1 expression, and maintained mitochondrial, peroxisomal, and mitoribosomal integrity, thereby attenuating tubular injury and albuminuria. Notably, empagliflozin treatment increased BHB, C/EBPβ, and Pck1 levels in noninjured mice without inducing organelle expansion, suggesting that Pck1 activation alone is insufficient to promote mitoribosome biogenesis under basal conditions. Collectively, these findings demonstrate that empagliflozin protects against CA/CPR-induced AKI and identify Pck1 as a key metabolic regulator linking ketone signaling to organelle resilience.

## 1. Introduction

Cardiac arrest followed by cardiopulmonary resuscitation (CA/CPR) causes severe whole-body ischemia–reperfusion injury, and acute kidney injury (AKI) is a major contributor to the associated morbidity and mortality. Despite advances in resuscitative care, no established pharmacological strategy is currently available to prevent renal injury after cardiac arrest. Recent clinical evidence suggests that sodium–glucose cotransporter 2 (SGLT2) inhibitors may reduce multiorgan injury after cardiac arrest, while experimental studies have demonstrated renoprotective effects in models of cisplatin nephrotoxicity, ischemia–reperfusion injury, and endotoxemia.

Among the proposed mechanisms underlying the protective effects of SGLT2 inhibitors, ketone body metabolism has emerged as a critical pathway. In particular, β-hydroxybutyrate (BHB) functions not only as an alternative energy substrate but also as a signaling metabolite that regulates transcriptional programs in proximal tubular cells. BHB activates C/EBPβ and induces phosphoenolpyruvate carboxykinase 1 (Pck1), thereby promoting adaptive metabolic responses. Hatano et al. [1] identified the BHB–C/EBPβ–Pck1 axis as a central regulator of renal gluconeogenesis. In addition, BHB itself exerts direct renoprotective effects by attenuating ischemia–reperfusion injury [2], suppressing NLRP3-mediated cisplatin nephrotoxicity [3,4], and ameliorating lipopolysaccharide-induced septic AKI [5], even in the absence of changes in endogenous BHB production.

Recent studies have further demonstrated that Pck1 plays important roles beyond gluconeogenesis. Pck1 deficiency impairs mitochondrial fitness and exacerbates renal injury [6], whereas Pck1-mediated cataplerosis preserves mitochondrial integrity and delays kidney disease progression [7]. Moreover, Pck1 contributes to peroxisomal redox homeostasis and protects against cardiac ischemia–reperfusion injury [8], highlighting its broader role in organelle maintenance.

Importantly, Pck1 has also been shown to maintain mitochondrial ribosomes (mitoribosomes) and support translation of mtDNA-encoded oxidative phosphorylation (OXPHOS) subunits [9]. These findings identify Pck1 as a key regulator of mitochondrial proteostasis, a concept that has gained increasing attention in studies investigating the relationship between mitochondrial dysfunction and kidney disease [10].

Taken together, these observations suggest that Pck1 acts as a metabolic integrator linking ketone signaling to mitochondrial and peroxisomal homeostasis. However, several important questions remain unresolved. It is unclear whether the BHB–C/EBPβ–Pck1 axis is disrupted during CA/CPR-induced AKI, whether SGLT2 inhibition restores this pathway through BHB elevation, and how Pck1 mediates organelle protection during severe ischemic stress. Because Pck1 is essential for maintaining mitoribosome integrity and promoting proximal tubular survival under metabolic stress, we hypothesized that CA/CPR suppresses the BHB–C/EBPβ–Pck1 axis, whereas SGLT2 inhibition restores this pathway by increasing BHB levels. We further hypothesized that Pck1 preserves mitochondrial and peroxisomal homeostasis through maintenance of mitoribosome integrity. To test these hypotheses, we combined experimental CA/CPR models, proximal tubule-specific Pck1 knockout mice, ultrastructural analyses, and pharmacological SGLT2 inhibition with empagliflozin.

## 2. Results

### 2.1. CA/CPR Selectively Reduces Circulating BHB Levels Among Experimental AKI Models

**Figure 1:**
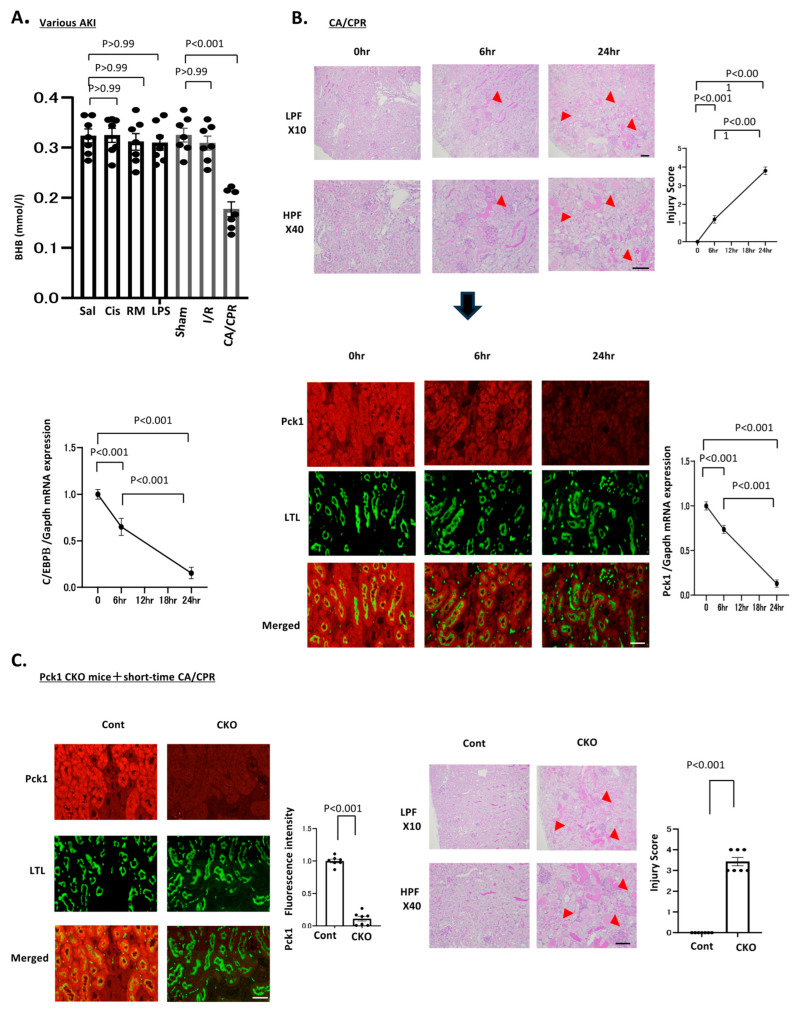
CA/CPR decreases circulating BHB and downregulates C/EBPβ–Pck1 signaling in proximal tubules. (**A**) Serum β-hydroxybutyrate (BHB) levels in multiple acute kidney injury (AKI) models. AKI was induced in 9-week-old male C57BL/6 mice using cisplatin (Cis), rhabdomyolysis (RM), endotoxemia (LPS), ischemia–reperfusion (I/R), or cardiac arrest/cardiopulmonary resuscitation (CA/CPR). Control mice received saline injections (Cis, RM, and LPS) or sham surgery (I/R and CA/CPR). Blood samples were collected 24 h after injury (*n* = 7 mice/group). Among all models examined, only CA/CPR significantly reduced circulating BHB levels. Data were analyzed using one-way ANOVA followed by Tukey’s post hoc test. (**B**) Time-course analysis of CA/CPR-induced AKI. CA/CPR was performed in 9-week-old mice, and kidneys were harvested at 0, 6, and 24 h after return of spontaneous circulation (ROSC) (*n* = 3 mice/time point). Periodic Acid–Schiff stain (PAS) demonstrated progressive tubular injury. Quantitative PCR showed time-dependent changes in C/EBPβ mRNA expression. Immunofluorescence staining (Pck1, green; biotinylated LTL, red) demonstrated progressive loss of Pck1 expression in LTL-positive proximal tubules. Data were analyzed using one-way ANOVA. (**C**) Pck1 deficiency exacerbates CA/CPR-induced AKI. Proximal tubule-specific Pck1 conditional knockout (CKO) mice were subjected to short-time CA/CPR (3-min arrest) at 9 weeks of age (*n* = 7 mice/group). Representative PAS-stained kidney sections from control (Cont) and CKO mice are shown; red arrowheads indicate lysed tubules. The right panel shows tubular injury scores. Pck1 CKO mice exhibited significantly greater tubular injury than control mice, indicating increased susceptibility to CA/CPR-induced AKI. Scale bars: 100 μm (immunofluorescence) and 50 μm (light microscopy). Data were analyzed using a two-tailed Student’s *t*-test. Exact *p*-values are indicated in the figures.

To determine whether metabolic alterations differ among etiologically distinct forms of AKI, we compared circulating BHB levels across several experimental models, including drug-induced AKI (cisplatin, rhabdomyolysis, and lipopolysaccharide [LPS]) and procedure-related AKI (ischemia–reperfusion injury and CA/CPR). Plasma BHB levels were measured 24 h after injury induction. Among all models examined, only CA/CPR caused a significant reduction in circulating BHB levels compared with sham controls (Figure 1A), whereas the other AKI models showed no evidence of BHB depletion. These findings indicate that CA/CPR uniquely induces endogenous BHB depletion, suggesting the presence of a metabolic vulnerability that may be therapeutically targeted through interventions that increase BHB levels, such as SGLT2 inhibition. Plasma BHB concentrations were 0.324 ± 0.013 mmol/L in Sham and 0.176 ± 0.01 mmol/L after CA/CPR (a 45.6% reduction; *p* < 0.001), while cisplatin (0.324 ± 0.01 mmol/L; *p* = 0.99), rhabdomyolysis (0.311 ± 0.016 mmol/L; *p* = 0.99), LPS (0.309 ± 0.01 mmol/L; *p* = 0.99), ischemia–reperfusion (0.308 ± 0.013 mmol/L; *p* = 0.99), and saline (0.323 ± 0.013 mmol/L; *p* = 0.99) showed no significant differences from Sham.

### 2.2. CA/CPR Dynamically Modulates the Renal BHB–C/EBPβ–Pck1 Axis

To investigate the mechanism underlying BHB depletion after CA/CPR, we examined temporal changes in the renal BHB–C/EBPβ–Pck1 axis. Kidney tissues collected at 0, 6, and 24 h after return of spontaneous circulation (ROSC) demonstrated rapid induction of C/EBPβ expression at 6 h, followed by partial decline at 24 h (Figure 1B). Pck1 expression exhibited a similar temporal pattern (Figure 1B). Immunofluorescence analysis further demonstrated that Pck1 expression was predominantly localized to proximal tubules. Quantitatively, renal C/EBPβ/GAPDH mRNA expression decreased from 1.000 ± 0.090 at 0 h to 0.650 ± 0.160 at 6 h (a 35.0% reduction; *p* < 0.001), and further to 0.153 ± 0.106 at 24 h (an 84.7% reduction; *p* < 0.001). Pck1/GAPDH mRNA expression similarly declined from 1.000 ± 0.080 at 0 h to 0.736 ± 0.080 at 6 h (a 26.4% reduction; *p* < 0.001), and to 0.130 ± 0.070 at 24 h (an 87.0% reduction; *p* < 0.001). These findings indicate that CA/CPR induces dynamic and time-dependent regulation of the C/EBPβ–Pck1 pathway in proximal tubular cells during the acute post-resuscitation phase, in association with systemic BHB depletion.

### 2.3. Pck1 Deficiency Markedly Increases Susceptibility to CA/CPR-Induced AKI

To determine whether reduced Pck1 expression contributes directly to CA/CPR-induced AKI, we used proximal tubule-specific Pck1 conditional knockout (Pck1 CKO) mice (Figure S1). Under basal conditions, Pck1 CKO mice exhibited no apparent AKI. However, following standard CA/CPR, all Pck1 CKO mice died, whereas control mice survived the procedure (Figure 1C). To permit histological evaluation, we established a milder injury model by reducing the duration of cardiac arrest to 3 min. Under these conditions, Pck1 CKO mice developed severe AKI with significantly higher tubular injury scores (Figure 1C). Quantitatively, tubular injury scores were 0.000 ± 0.000 in control mice and 3.428 ± 0.534 in Pck1 CKO mice (*p* < 0.001). These findings suggest that Pck1 is essential for protecting proximal tubular cells against CA/CPR-induced ischemic injury, even under relatively mild ischemic stress.

### 2.4. Pck1 Loss Causes Albuminuria and Mitochondrial Ribosomal Loss After CA/CPR

**Figure 2:**
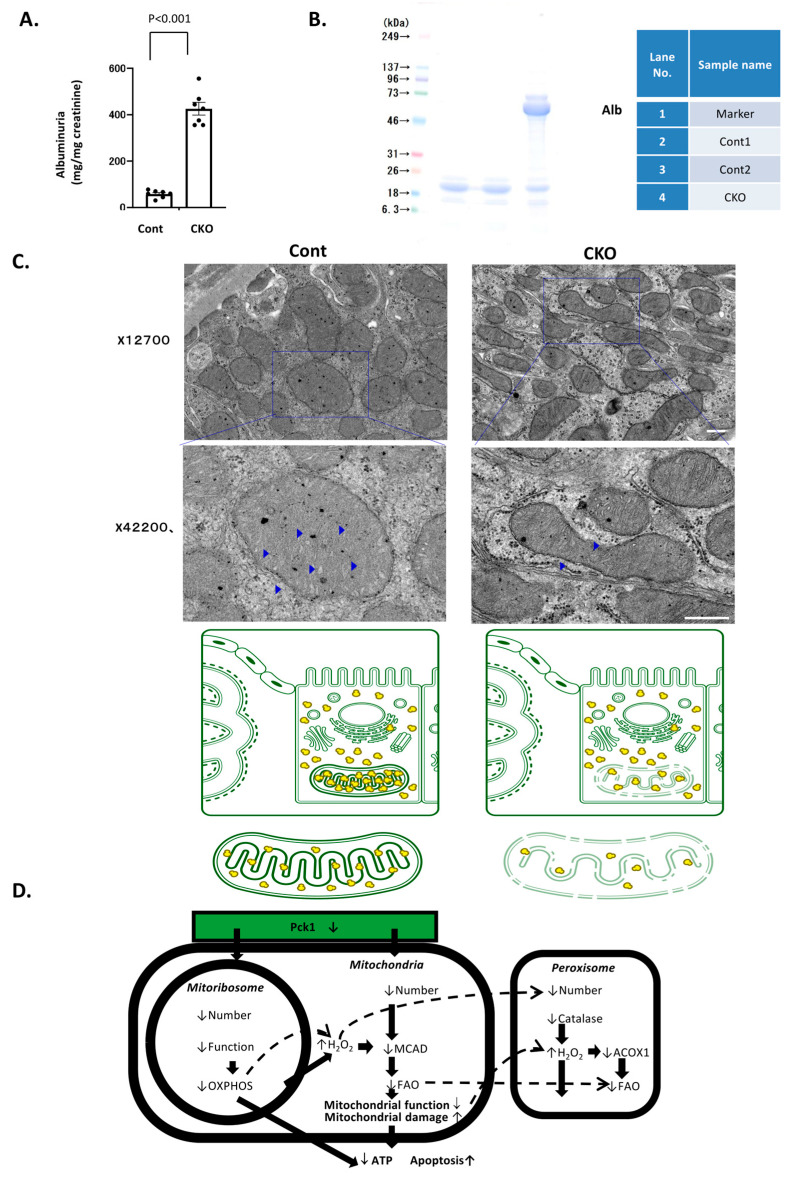
Albuminuria and ultrastructural abnormalities in proximal tubule-specific Pck1 CKO mice subjected to short-time CA/CPR. (**A**) Urinary albumin excretion in control (Cont) and proximal tubule-specific Pck1 CKO mice after short-time CA/CPR. Nineteen-week-old Cont and CKO mice were subjected to short-time CA/CPR, and urinary albumin-to-creatinine ratios were measured (*n* = 7 mice/group). CKO mice exhibited significantly increased albuminuria compared with Cont mice. Data were analyzed using a two-tailed Student’s *t*-test. Exact *p*-values are indicated in the figure. (**B**) SDS-PAGE analysis of urinary proteins. Urine samples from 9-week-old Cont and CKO mice were subjected to 15% SDS-PAGE followed by Coomassie Brilliant Blue staining. CKO mice exhibited increased albumin-sized bands, consistent with elevated urinary albumin excretion. (**C**) Transmission electron microscopy (TEM) of proximal tubules. Representative electron micrographs from each group are shown. Blue squares indicate enlarged regions, and blue arrowheads denote mitoribosomes, which were markedly reduced in CKO mice subjected to short-time CA/CPR. Kidney tissues were fixed and embedded in Epon epoxy resin. Ten proximal tubules per mouse were randomly selected for ultrastructural analysis. Scale bar: 50 nm. (**D**) Schematic illustration of the proposed mechanism underlying CA/CPR-induced AKI in Pck1 CKO mice. Loss of Pck1 reduced expression of mitoribosomal and mitochondrial proteins, resulting in tubular injury and ultrastructural abnormalities in mitochondria and mitoribosomes. Mitochondrial dysfunction was associated with peroxisomal injury, including reduced catalase expression and increased H_2_O_2_ accumulation, leading to tubular apoptosis. Elevated oxidative stress further impaired ACOX1 and MCAD expression, reducing fatty acid oxidation and ATP production.

At 24 h after short-duration CA/CPR, Pck1 CKO mice exhibited markedly increased albuminuria compared with control mice (Figure 2A). SDS-PAGE analysis confirmed a prominent urinary albumin band in Pck1 CKO mice, whereas control mice displayed only faint signals (Figure 2B), indicating substantial proximal tubular dysfunction. Quantitatively, albuminuria levels were 58.3 ± 16.0 mg/mg creatinine in control mice and 425.9 ± 72.4 mg/mg creatinine in Pck1 CKO mice (a 7.3-fold increase; *p* < 0.001).

Transmission electron microscopy revealed a striking reduction in mitochondrial ribosomes (mitoribosomes) in Pck1 CKO mice at both low- and high-magnification views, whereas control mice retained well-preserved ribosomal structures (Figure 2C). These findings are consistent with our previous report demonstrating that Pck1 is required for maintenance of mitochondrial ribosome abundance and function [9].

The observed ultrastructural abnormalities suggest that loss of Pck1 disrupts mitochondrial ribosomal integrity (Figure 2D), thereby impairing translation of mtDNA-encoded OXPHOS subunits. This defect in mitochondrial translation may subsequently reduce medium-chain acyl-CoA dehydrogenase (MCAD) expression and induce secondary peroxisomal dysfunction, as shown in the subsequent analyses.

### 2.5. Pck1 Deficiency Disrupts Mitochondrial and Peroxisomal Homeostasis After CA/CPR

**Figure 3:**
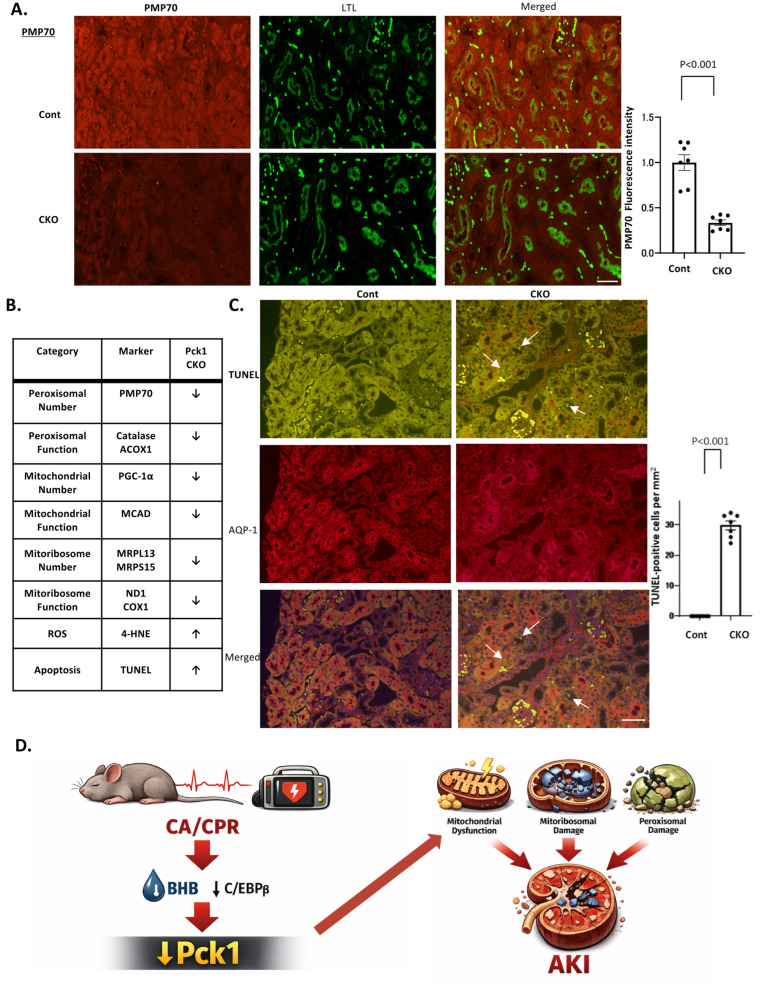
Pck1 deficiency disrupts peroxisomal, mitochondrial, and mitoribosomal homeostasis after CA/CPR. (**A**) Representative immunofluorescence staining for the peroxisomal marker PMP70 in control (Cont) and proximal tubule-specific Pck1 CKO mice after short-time CA/CPR (*n* = 7 mice/group). Quantitative fluorescence intensity is shown in the accompanying graphs. Scale bar: 50 μm. (**B**) Summary of organelle marker expression in Cont and CKO mice. CKO mice exhibited reduced expression of peroxisomal proteins (PMP70, catalase, and acyl-CoA oxidase 1 [ACOX1]), mitochondrial proteins (PGC-1α and medium-chain acyl-CoA dehydrogenase [MCAD]), and mitoribosomal proteins (MRPL13 and MRPS15), indicating impaired organelle number and function. (**C**) Apoptosis in proximal tubules of Cont and CKO mice (*n* = 7 mice/group). Representative immunofluorescence images show double staining for TUNEL (green) and AQP1 (red), a proximal tubule marker. White arrows indicate apoptotic tubular cells. Scale bar: 50 μm. (**D**) Schematic illustration of the proposed mechanism of CA/CPR-induced AKI. CA/CPR-induced reductions in circulating BHB and C/EBPβ expression suppress Pck1 expression in proximal tubules, leading to mitoribosome loss and impaired mitochondrial and peroxisomal function. Peroxisomal dysfunction, impaired fatty acid oxidation, oxidative stress, and mitochondrial injury collectively promote tubular apoptosis and AKI. The schematic illustration in panel (**D**) was generated using artificial intelligence-based software and manually verified for accuracy. Data were analyzed using a two-tailed Student’s *t*-test. Exact *p*-values are indicated in the figures.

To investigate the effects of Pck1 deficiency on organelle homeostasis after CA/CPR, we evaluated mitochondrial and peroxisomal markers in proximal tubules from Pck1 CKO mice subjected to short-duration CA/CPR. Immunofluorescence staining demonstrated marked reduction in the peroxisomal membrane protein PMP70 in Pck1 CKO mice compared with controls (Figure 3A). Consistently, catalase and ACOX1, key enzymes involved in peroxisomal β-oxidation, were also significantly reduced in Pck1-deficient kidneys (Figures S2–S4). Quantitatively, PMP70 fluorescence intensity decreased from 1.000 ± 0.229 in controls to 0.336 ± 0.080 in CKO mice (a 66.4% reduction; *p* < 0.001). Catalase decreased from 1.000 ± 0.118 to 0.582 ± 0.171 (a 41.8% reduction; *p* < 0.001), and ACOX1 decreased from 1.000 ± 0.107 to 0.778 ± 0.097 (a 22.2% reduction; *p* < 0.001).

Mitochondrial markers showed similar alterations. Expression of PGC-1α, a master regulator of mitochondrial biogenesis, and MCAD, a representative enzyme involved in mitochondrial fatty acid oxidation, was significantly reduced in Pck1-deficient proximal tubules (Figures S5 and S6). PGC-1α decreased from 1.000 ± 0.118 to 0.785 ± 0.089 (a 21.5% reduction; *p* < 0.001), and MCAD decreased from 1.000 ± 0.105 to 0.754 ± 0.095 (a 24.6% reduction; *p* < 0.001). In contrast, expression of 4-HNE, a marker of lipid peroxidation and oxidative stress, was markedly increased (Figure S7), indicating enhanced reactive oxygen species generation secondary to mitochondrial dysfunction. 4-HNE increased from 3.659 ± 4.886 in controls to 313.857 ± 55.119 in CKO mice (an 85.7-fold increase; *p* < 0.001). Collectively, these findings demonstrate that Pck1 deficiency disrupts both mitochondrial and peroxisomal homeostasis, resulting in impaired β-oxidation and increased oxidative stress following CA/CPR.

### 2.6. Pck1 Deficiency Induces Coordinated Organelle Dysfunction, Including Mitoribosome Loss

The coordinated nature of these abnormalities is summarized in Figure 3B, which integrates defects across multiple organelle systems. Notably, Pck1 deficiency caused marked reductions in the mitochondrial ribosomal proteins MRPL13 and MRPS15 (Figure S8A), indicating substantial loss of mitochondrial ribosome abundance. Furthermore, expression of ND1 and COX1, mtDNA-encoded OXPHOS subunits that depend on mitochondrial ribosomal translation, was also significantly decreased in Pck1 CKO mice (Figure S8B), demonstrating impaired mitochondrial translational function. Together with the reductions in PGC-1α and MCAD and the increase in 4-HNE, these findings indicate widespread organelle dysfunction in Pck1-deficient proximal tubules following CA/CPR.

### 2.7. Pck1 Deficiency Enhances Proximal Tubular Apoptosis After CA/CPR

To determine whether organelle dysfunction was associated with increased cell death, we performed dual immunofluorescence staining for TUNEL and AQP1. Pck1 CKO mice exhibited significantly increased numbers of apoptotic proximal tubular cells compared with control mice (Figure 3C). TUNEL-positive cells increased from 0 ± 0 per mm^2^ in controls to 29.857 ± 3.891 per mm^2^ in CKO mice (*p* < 0.001). These findings indicate that loss of Pck1 sensitizes proximal tubular cells to apoptosis following CA/CPR, consistent with the observed mitochondrial and peroxisomal abnormalities.

### 2.8. Loss of Pck1 Links CA/CPR-Induced BHB Depletion to Mitochondrial Ribosomal Failure and Organelle Collapse

A mechanistic summary of these findings is presented in Figure 3D. CA/CPR-induced depletion of circulating BHB suppresses C/EBPβ and Pck1 expression in proximal tubules. Reduced Pck1 expression leads to loss of mitochondrial ribosomes, as evidenced by decreased MRPL13 and MRPS15 expression (Figure S8A), and impaired mitochondrial translational activity, as reflected by reduced ND1 and COX1 expression (Figure S8B). Quantitatively, MRPL13 and MRPS15 expression was significantly reduced in Pck1-deficient kidneys (*p* = 0.005), whereas ND1 and COX1 expression showed a more pronounced reduction (*p* < 0.001). These alterations impair translation of mtDNA-encoded OXPHOS subunits, resulting in mitochondrial dysfunction. The mitochondrial translational defect subsequently contributes to reduced MCAD expression, impaired peroxisomal metabolism, increased oxidative stress, and ultimately apoptosis. Collectively, these findings identify Pck1 as a critical metabolic safeguard that preserves mitoribosome integrity and organelle homeostasis during ischemic stress.

### 2.9. Empagliflozin Restores the BHB–C/EBPβ–Pck1 Axis and Attenuates CA/CPR-Induced AKI

**Figure 4:**
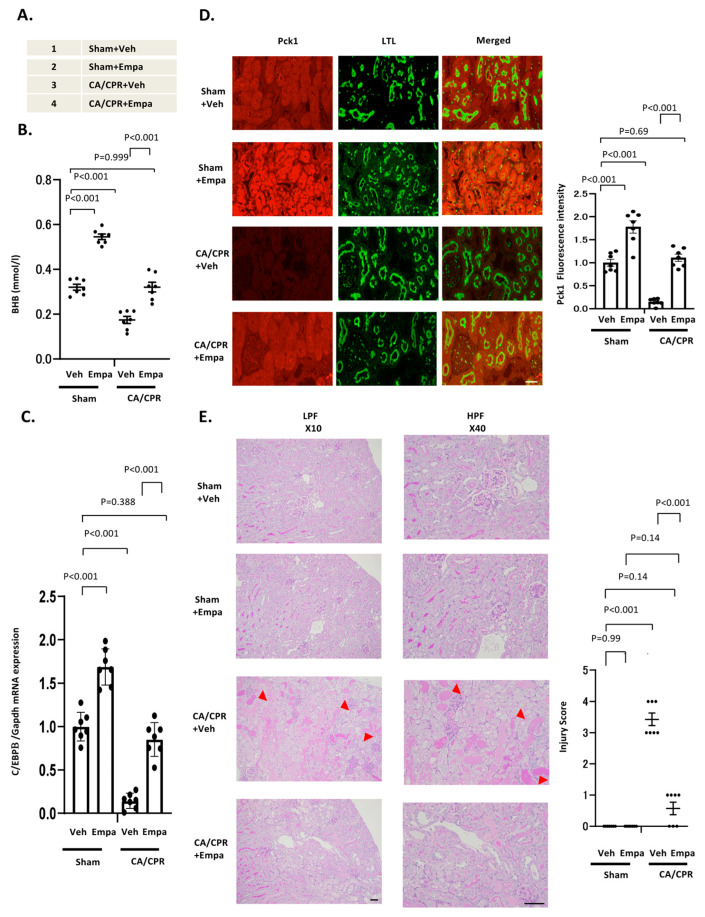
Empagliflozin restores BHB–C/EBPβ–Pck1 signaling and attenuates tubular injury after CA/CPR. (**A**) Experimental design for empagliflozin administration. Eight-week-old male C57BL/6J mice received daily oral gavage of vehicle (0.5% methylcellulose) or empagliflozin (10 mg/kg in 0.5% methylcellulose) for 7 days. Mice were then assigned to four groups: Veh + Sham, Empa + Sham, Veh + CA/CPR, and Empa + CA/CPR (*n* = 7 mice/group). CA/CPR was induced using the standard 7.5-min cardiac arrest protocol. (**B**) Serum BHB levels in each experimental group. Blood was collected 24 h after sham or CA/CPR procedures. Empagliflozin increased BHB levels in sham-operated mice and prevented the CA/CPR-induced decline in circulating BHB. (**C**) Renal C/EBPβ mRNA expression. Quantitative PCR analysis demonstrated that CA/CPR markedly reduced C/EBPβ expression in vehicle-treated mice, whereas empagliflozin preserved C/EBPβ expression after CA/CPR. (**D**) Representative immunofluorescence staining for Pck1 in proximal tubules. Kidney cryosections were stained for Pck1 (green) and biotinylated LTL (red). Empagliflozin increased Pck1 expression in sham-operated mice and prevented CA/CPR-induced loss of Pck1 expression in proximal tubules. (**E**) Representative PAS and tubular injury scores. PAS-stained kidney sections are shown; red arrowheads indicate lysed tubules. Veh + CA/CPR mice exhibited severe tubular injury, which was significantly attenuated in Empa + CA/CPR mice. Scale bars: 100 μm (immunofluorescence) and 50 μm (light microscopy). Quantitative data were analyzed using two-way ANOVA followed by Tukey’s post hoc test (*n* = 7 mice/group). Exact *p*-values are indicated in the figures.

After 7 days of treatment, 9-week-old mice underwent sham or CA/CPR procedures. Plasma BHB levels were significantly increased in Sham + Empa mice compared with Sham + Veh mice, confirming the expected ketogenic effect of SGLT2 inhibition. Consistent with previous observations, Veh + CA/CPR mice exhibited marked reductions in circulating BHB levels, whereas Empa + CA/CPR mice maintained BHB levels comparable to those of Veh + Sham mice (Figure 4A). Quantitatively, plasma BHB concentrations were 0.320 ± 0.0347 mmol/L in Veh + Sham, 0.545 ± 0.0328 mmol/L in Empa + Sham (a 70.3% increase; *p* < 0.001), 0.174 ± 0.0413 mmol/L in Veh + CA/CPR (a 45.6% reduction vs. Sham; *p* < 0.001), and 0.320 ± 0.057 mmol/L in Empa + CA/CPR (a 84.0% increase vs. Veh + CA/CPR; *p* < 0.001). These findings demonstrate that empagliflozin prevents CA/CPR-induced BHB depletion.

Real-time PCR analysis showed that C/EBPβ mRNA expression was significantly increased in Sham + Empa mice, consistent with BHB-mediated transcriptional activation. Immunofluorescence analysis further demonstrated increased Pck1 expression in proximal tubules from Sham + Empa mice. In contrast, Veh + CA/CPR mice exhibited marked reductions in both C/EBPβ and Pck1 expression, whereas Empa + CA/CPR mice maintained expression levels comparable to those observed in Veh + Sham mice (Figure 4B). Quantitatively, C/EBPβ/GAPDH mRNA expression was 1.000 ± 0.165 in Veh + Sham, 1.688 ± 0.207 in Empa + Sham (a 68.8% increase; *p* < 0.001), 0.143 ± 0.088 in Veh + CA/CPR (an 85.7% reduction; *p* < 0.001), and 0.852 ± 0.194 in Empa + CA/CPR (a 5.9-fold increase vs. Veh + CA/CPR; *p* < 0.001). Pck1 fluorescence intensity was 1.000 ± 0.195 in Veh + Sham, 1.777 ± 0.355 in Empa + Sham (a 77.7% increase; *p* < 0.001), 0.147 ± 0.074 in Veh + CA/CPR (an 85.3% reduction; *p* < 0.001), and 1.108 ± 0.200 in Empa + CA/CPR (a 7.5-fold increase vs. Veh + CA/CPR; *p* < 0.001).

Histological analysis further confirmed that empagliflozin markedly attenuated tubular injury following CA/CPR (Figure 4C). Quantitatively, tubular injury scores were 0 ± 0 in Veh + Sham, 0 ± 0 in Empa + Sham, 3.428 ± 0.534 in Veh + CA/CPR, and 0.571 ± 0.534 in Empa + CA/CPR (an 83.3% reduction vs. Veh + CA/CPR; *p* < 0.001). Together, these findings demonstrate that empagliflozin restores the BHB–C/EBPβ–Pck1 signaling axis and protects against CA/CPR-induced AKI (Figure 4D).

### 2.10. Empagliflozin Preserves Proximal Tubular Function and Mitoribosome Integrity After CA/CPR

**Figure 5:**
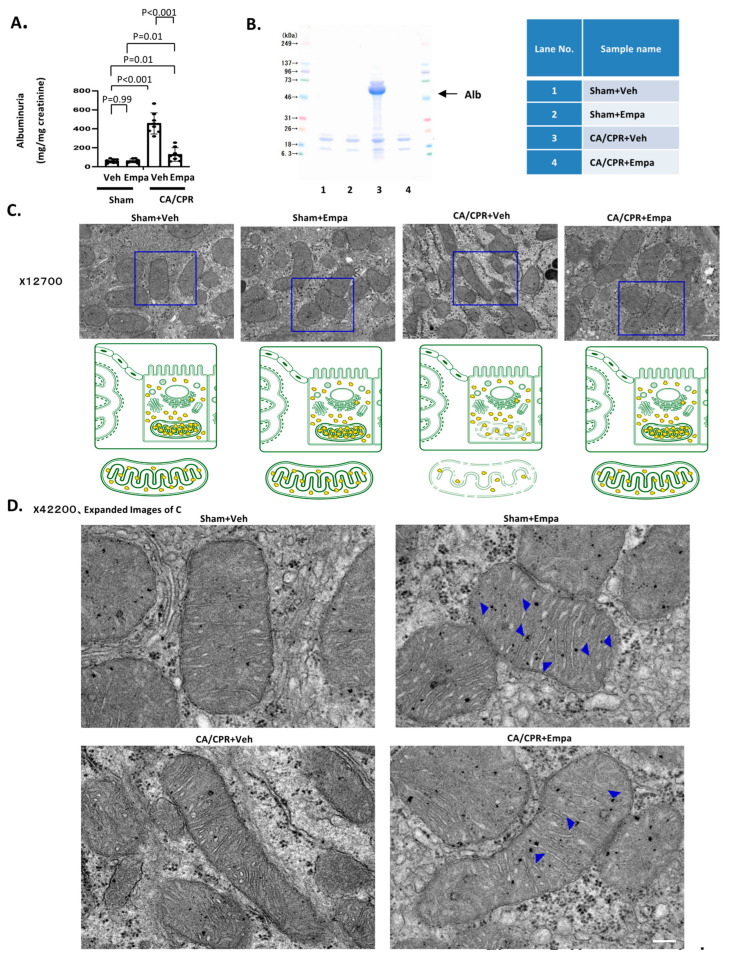
Empagliflozin reduces albuminuria and preserves mitoribosomes after CA/CPR. (**A**) Urinary albumin excretion in the four experimental groups. Urinary albumin-to-creatinine ratios were measured in Veh + Sham, Empa + Sham, Veh + CA/CPR, and Empa + CA/CPR mice. Empagliflozin significantly attenuated CA/CPR-induced albuminuria. Data were analyzed using one-way ANOVA with Bonferroni correction. Exact *p*-values are indicated in the figure. (**B**) SDS-PAGE analysis of urinary proteins. Urine samples from the four groups were analyzed by 15% SDS-PAGE followed by Coomassie Brilliant Blue staining. Veh + CA/CPR mice exhibited prominent albumin-sized bands, which were markedly reduced in Empa + CA/CPR mice. (**C**) Representative TEM images of proximal tubules. Electron micrographs from each group are shown, with schematic illustrations below. Blue squares indicate regions enlarged in panel (**D**). (**D**) Enlarged TEM images highlighting mitoribosomes. High-magnification images from panel (**C**) are shown. Blue arrowheads indicate mitoribosomes. Kidney tissues were embedded in Epon epoxy resin for TEM analysis. Ten proximal tubules per mouse were randomly selected for ultrastructural evaluation. Scale bar: 500 nm. Empagliflozin preserved mitoribosome abundance and morphology after CA/CPR.

Vehicle-treated CA/CPR mice exhibited significant increases in urinary albumin excretion, whereas Empa + CA/CPR mice showed markedly reduced albuminuria, with levels comparable to those in Veh + Sham mice (Figure 5A). SDS-PAGE analysis of urinary proteins confirmed these findings (Figure 5B). Quantitatively, albuminuria levels were 60.275 ± 20.421 mg/mg creatinine in Veh + Sham, 62.200 ± 19.060 mg/mg creatinine in Empa + Sham (a 3.2% increase; *p* < 0.001), 457.087 ± 112.121 mg/mg creatinine in Veh + CA/CPR (a 7.6-fold increase vs. Veh + Sham; *p* < 0.001), and 129.487 ± 71.109 mg/mg creatinine in Empa + CA/CPR (a 71.7% reduction vs. Veh + CA/CPR; *p* < 0.001).

Transmission electron microscopy demonstrated marked differences in mitochondrial ribosomal density among the experimental groups. Sham + Empa mice exhibited increased mitoribosome abundance, whereas Veh + CA/CPR mice showed marked mitoribosome loss. In contrast, Empa + CA/CPR mice retained mitochondrial ribosomal density comparable to that observed in Veh + Sham mice (Figure 5C).

### 2.11. Empagliflozin Preserves Mitochondrial and Peroxisomal Homeostasis After CA/CPR

**Figure 6:**
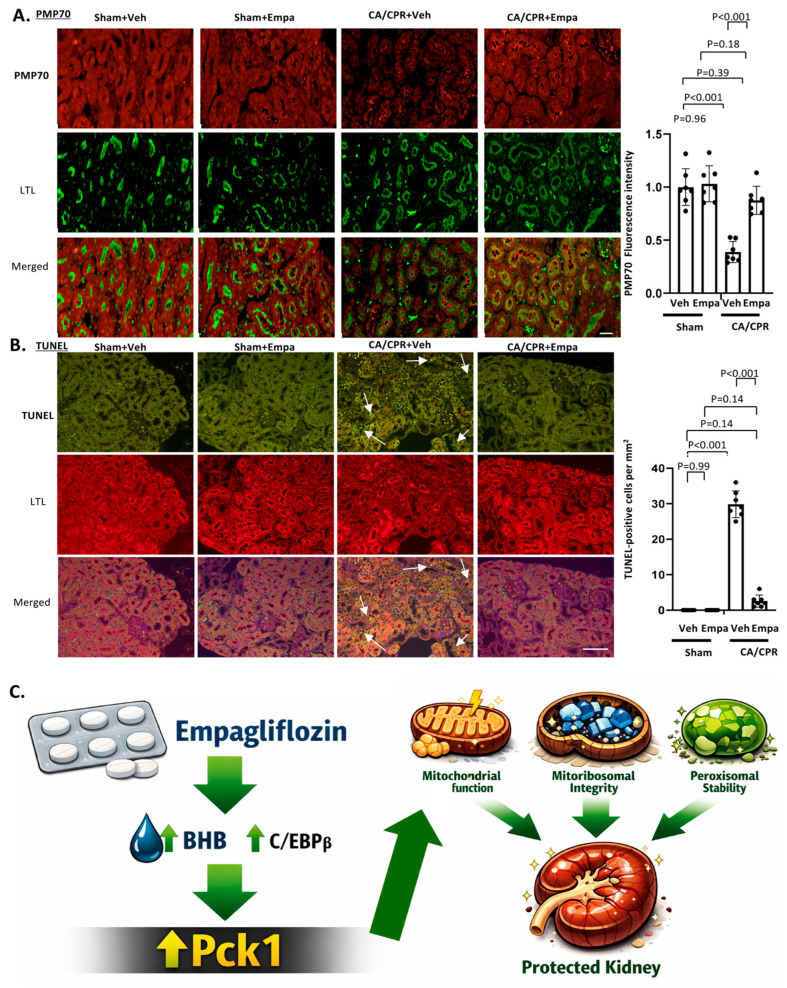
Empagliflozin preserves mitochondrial, peroxisomal, and mitoribosomal integrity after CA/CPR. (**A**) Representative immunostaining for the peroxisomal marker PMP70. Kidney sections from Veh + Sham, Empa + Sham, Veh + CA/CPR, and Empa + CA/CPR mice were stained for PMP70. Empagliflozin preserved PMP70 expression after CA/CPR, whereas Veh + CA/CPR mice showed marked reductions. Quantitative fluorescence intensity is shown in the accompanying graph. (**B**) TUNEL staining of apoptotic tubular cells. Representative images of TUNEL-positive apoptotic cells (arrows) in proximal tubules are shown (*n* = 7 mice/group). Empagliflozin significantly reduced CA/CPR-induced tubular apoptosis. Quantitative analysis of TUNEL-positive cells is shown in the accompanying graph. (**C**) Schematic illustration of the protective effects of empagliflozin in CA/CPR-induced AKI. Empagliflozin increased circulating BHB levels and enhanced C/EBPβ-mediated Pck1 expression in proximal tubules. Preservation of Pck1 expression maintained mitochondrial, peroxisomal, and mitoribosomal integrity, thereby reducing tubular injury and protecting against AKI. The schematic illustration in panel (**C**) was created using artificial intelligence-based software and carefully checked to ensure scientific correctness. Scale bars: 100 μm (immunofluorescence) and 50 μm (light microscopy). Quantitative data were analyzed using one-way ANOVA followed by Bonferroni correction (*n* = 7 mice/group). Exact *p*-values are indicated in the figures.

Immunofluorescence staining demonstrated marked reduction in PMP70 expression in Veh + CA/CPR mice, whereas Empa + CA/CPR mice maintained PMP70 expression at levels comparable to those of Veh + Sham mice (Figure 6A). Similarly, expression of peroxisomal functional markers, including catalase and ACOX1, was reduced in Veh + CA/CPR mice but preserved in Empa + CA/CPR mice (Figures S9–S11). Quantitatively, PMP70 fluorescence intensity was 1.000 ± 0.173 in Veh + Sham, 1.031 ± 0.169 in Empa + Sham (a 3.1% increase; *p* < 0.001), 0.388 ± 0.099 in Veh + CA/CPR (a 61.2% reduction; *p* < 0.001), and 0.875 ± 0.133 in Empa + CA/CPR (a 125.6% increase vs. Veh + CA/CPR; *p* < 0.001). Catalase expression was 1.000 ± 0.126 in Veh + Sham, 1.065 ± 0.208 in Empa + Sham (a 6.5% increase; *p* < 0.001), 0.595 ± 0.163 in Veh + CA/CPR (a 40.5% reduction; *p* < 0.001), and 0.943 ± 0.144 in Empa + CA/CPR (a 58.5% increase vs. Veh + CA/CPR; *p* < 0.001). ACOX1 expression was 1.000 ± 0.093 in Veh + Sham, 1.029 ± 0.108 in Empa + Sham (a 2.9% increase; *p* < 0.001), 0.765 ± 0.112 in Veh + CA/CPR (a 23.5% reduction; *p* < 0.001), and 1.022 ± 0.128 in Empa + CA/CPR (a 33.6% increase vs. Veh + CA/CPR; *p* < 0.001).

Mitochondrial markers showed parallel changes. Expression of PGC-1α, a regulator of mitochondrial biogenesis, and MCAD, a mitochondrial fatty acid oxidation enzyme, was markedly reduced in Veh + CA/CPR mice but preserved in Empa + CA/CPR mice (Figures S12 and S13). PGC-1α expression was 1.000 ± 0.040 in Veh + Sham, 1.060 ± 0.139 in Empa + Sham (a 6.0% increase; *p* < 0.001), 0.705 ± 0.059 in Veh + CA/CPR (a 29.5% reduction; *p* < 0.001), and 0.975 ± 0.128 in Empa + CA/CPR (a 38.3% increase vs. Veh + CA/CPR; *p* < 0.001). MCAD expression was 1.000 ± 0.120 in Veh + Sham, 1.050 ± 0.077 in Empa + Sham (a 5.0% increase; *p* < 0.001), 0.780 ± 0.065 in Veh + CA/CPR (a 22.0% reduction; *p* < 0.001), and 1.014 ± 0.086 in Empa + CA/CPR (a 30.0% increase vs. Veh + CA/CPR; *p* < 0.001). Conversely, expression of 4-HNE, a marker of lipid peroxidation and oxidative stress, was markedly increased in Veh + CA/CPR mice and substantially suppressed by empagliflozin treatment (Figure S14). 4-HNE levels were 6.385 ± 4.112 in Veh + Sham, 4.146 ± 5.216 in Empa + Sham (a 35.0% decrease; *p* < 0.001), 299.728 ± 34.183 in Veh + CA/CPR (a 46.9-fold increase; *p* < 0.001), and 23.066 ± 18.572 in Empa + CA/CPR (a 92.3% reduction vs. Veh + CA/CPR; *p* < 0.001).

### 2.12. Empagliflozin Preserves Mitochondrial Ribosomal Abundance and Function After CA/CPR

Immunohistochemical analysis demonstrated that expression of MRPL13 and MRPS15, markers of mitochondrial ribosomal abundance, was markedly reduced in Veh + CA/CPR mice but preserved in Empa + CA/CPR mice (Figure S15A). Both MRPL13 and MRPS15 showed significant reductions in Veh + CA/CPR mice (*p* = 0.005 and *p* = 0.006).

Similarly, ND1 and COX1, mtDNA-encoded OXPHOS subunits that require mitochondrial ribosomal translation, were reduced in Veh + CA/CPR mice but maintained in Empa + CA/CPR mice (Figure S15B). Both ND1 and COX1 were significantly reduced in Veh + CA/CPR mice (*p* < 0.001). These findings indicate that empagliflozin preserves both mitochondrial ribosomal abundance and mitochondrial translational function, thereby maintaining mitochondrial protein synthesis capacity (Figure 5D).

### 2.13. Empagliflozin Reduces Tubular Apoptosis and Provides Multilevel Organelle Protection

Dual TUNEL and AQP1 staining demonstrated a significant increase in apoptotic proximal tubular cells in Veh + CA/CPR mice, whereas Empa + CA/CPR mice exhibited markedly fewer TUNEL-positive cells (Figure 6B). Quantitatively, TUNEL-positive cells were 0 ± 0 per mm^2^ in Veh + Sham, 0 ± 0 in Empa + Sham, 29.857 ± 3.761 in Veh + CA/CPR, and 2.571 ± 1.718 in Empa + CA/CPR (a 91.4% reduction vs. Veh + CA/CPR; *p* < 0.001).

Collectively, these findings indicate that empagliflozin elevates circulating BHB levels, enhances C/EBPβ and Pck1 expression, preserves mitoribosomes and OXPHOS translation, maintains mitochondrial and peroxisomal metabolism, suppresses oxidative stress and apoptosis, and ultimately attenuates CA/CPR-induced AKI (Figure 6C).

In summary, our findings identify Pck1 as a central metabolic safeguard that preserves mitoribosome integrity, mitochondrial and peroxisomal metabolism, and proximal tubular homeostasis during cardiac arrest/cardiopulmonary resuscitation-induced systemic ischemia. CA/CPR-induced depletion of BHB suppresses the C/EBPβ–Pck1 signaling axis, resulting in coordinated organelle dysfunction and AKI, whereas empagliflozin preserves this ketone-dependent pathway and confers multilevel organelle protection. These findings establish the BHB–C/EBPβ–Pck1 axis as a critical determinant of tubular resilience during severe ischemic stress associated with cardiac arrest.

### 2.14. Mitochondrial Dysfunction in Pck1CKO Mice

Primary tubular epithelial cells (TECs) isolated from CKO mice exhibited marked impairment of mitochondrial respiration. Basal and maximal oxygen consumption rates (OCR) were significantly reduced in CKO TECs compared with controls (*p* < 0.001; Figure S18A), indicating diminished OXPHOS capacity. JC-1 staining demonstrated a significant reduction in the red/green fluorescence ratio (*p* < 0.001; Figure S18B), consistent with loss of mitochondrial membrane potential. MitoSOX fluorescence was significantly increased (*p* < 0.001; Figure S18C), indicating elevated mitochondrial ROS production. ATP content was also significantly decreased (*p* < 0.001; Figure S18D). These findings collectively demonstrate that Pck1 deficiency leads to profound mitochondrial dysfunction at the functional level.

### 2.15. Electron Transport Chain (ETC) Complex Activities in CKO Kidneys

Mitochondria isolated from CKO kidneys showed selective reductions in ETC complex activities. Activities of CI, CIII, CIV, and CV were significantly decreased (all *p* < 0.01; Figure S19), whereas CII activity was increased (*p* = 0.003; Figure S19), likely reflecting compensatory upregulation of the nDNA-encoded complex. These results are consistent with impaired mitoribosomal translation of mtDNA-encoded subunits and explain the reduced OCR observed in CKO TECs.

### 2.16. Catalase and ACOX1 Activities in CKO Kidneys

Peroxisomal enzymatic function was significantly impaired in CKO kidneys. Catalase activity was markedly reduced (*p* = 0.001; Figure S20A), indicating diminished H_2_O_2_ detoxification capacity. ACOX1 activity was also significantly decreased (*p* = 0.001; Figure S20B), demonstrating impaired peroxisomal β-oxidation. These findings support the conclusion that Pck1 deficiency disrupts both mitochondrial and peroxisomal metabolic homeostasis.

### 2.17. Empagliflozin Preserves Mitochondrial Function Following CA/CPR

Empagliflozin treatment significantly improved mitochondrial function in TECs isolated from CA/CPR mice. OCR was markedly reduced in Veh + CA/CPR TECs but was restored to near-baseline levels in Empa + CA/CPR TECs (*p* < 0.001; Figure S21A). JC-1 red/green ratios were significantly improved by empagliflozin (*p* < 0.001; Figure S21B), indicating preservation of membrane potential. MitoSOX fluorescence was reduced in Empa + CA/CPR TECs (*p* < 0.001; Figure S21C), demonstrating suppression of mitochondrial ROS. ATP content was significantly higher in Empa + CA/CPR TECs compared with Veh + CA/CPR TECs (*p* < 0.001; Figure S21D). These results indicate that empagliflozin protects mitochondrial function after CA/CPR.

### 2.18. ETC Complex Activities in Empagliflozin-Treated Kidneys

CA/CPR significantly decreased CI, CIII, CIV, and CV activities, whereas empagliflozin treatment restored these activities toward baseline levels (all *p* < 0.05; Figure S22). CII activity remained unchanged across groups, consistent with its nDNA-encoded origin. These findings demonstrate that empagliflozin preserves mitoribosomal translation-dependent ETC complex activities following CA/CPR.

### 2.19. Catalase and ACOX1 Activities in Empagliflozin-Treated Kidneys

CA/CPR significantly reduced catalase and ACOX1 activities in Veh-treated mice (both *p* < 0.001; Figure S23A,B). Empagliflozin treatment restored both enzymatic activities in CA/CPR kidneys (*p* < 0.001), indicating preserved peroxisomal H_2_O_2_ detoxification and β-oxidation. No significant differences were observed between Veh + Sham and Empa + Sham groups.

### 2.20. Positive and Negative Controls for Immunofluorescence

Positive and negative controls were performed for all immunofluorescence experiments to validate antibody specificity. Kidneys from healthy 9-week-old wild-type mice served as positive controls for PCK1, LTL, AQP1, PMP70, ACOX1, catalase, PGC1α, and MCAD (Figures S24–S32). A 15-month-old wild-type kidney was used as the positive control for 4-HNE (Figure S33). Negative controls were generated by immune depletion using antigen-blocked primary antibodies, which abolished all specific signals. For TUNEL staining, omission of the TdT enzyme eliminated nuclear labeling (Figure S26). These controls confirm the specificity and reliability of all immunofluorescence signals presented in the main and Supplementary Materials.

## 3. Discussion

Acute kidney injury is a common and serious complication following cardiac arrest and cardiopulmonary resuscitation (CA/CPR), reflecting the profound systemic ischemia–reperfusion injury that characterizes post-cardiac arrest syndrome [11,12,13,14,15,16,17,18]. Despite its clinical significance, no established pharmacological therapy is currently available to prevent CA/CPR-induced AKI. In the present study, we identified the ketone-dependent BHB–C/EBPβ–Pck1 axis as a critical determinant of proximal tubular resilience under severe metabolic stress.

Our findings demonstrate that CA/CPR uniquely reduces circulating BHB levels, in contrast to other AKI models, including cisplatin nephrotoxicity [3,4] and LPS-induced sepsis [5]. This selective depletion of BHB suppresses a transcriptional program required for maintenance of mitochondrial, peroxisomal, and mitoribosomal homeostasis. Because BHB activates C/EBPβ and induces Pck1 expression, reduced BHB availability provides a mechanistic explanation for the coordinated organelle dysfunction observed after CA/CPR. These observations extend previous studies showing that BHB exerts renoprotective effects in ischemic, toxic, and inflammatory AKI even when endogenous BHB levels are unchanged [19,20,21].

Our results further establish Pck1 as an essential metabolic safeguard in proximal tubular cells. In addition to its canonical role in gluconeogenesis, Pck1 regulates mitochondrial translation, cataplerosis, and peroxisomal redox homeostasis [6,7,8]. Pck1 deficiency caused marked loss of mitoribosomes, impaired translation of mtDNA-encoded OXPHOS subunits, peroxisomal dysfunction, and increased oxidative stress, ultimately leading to tubular apoptosis. These findings position Pck1 as a central regulator of mitochondrial proteostasis during metabolic stress.

Under conditions of CA/CPR-induced metabolic collapse, empagliflozin restored circulating BHB levels, reactivated C/EBPβ and Pck1 expression, and preserved mitoribosome abundance and mitochondrial translational activity. Notably, empagliflozin did not increase mitoribosome abundance in uninjured kidneys, indicating that activation of Pck1 alone is insufficient to induce mitoribosome biogenesis. This observation suggests the presence of at least two distinct regulatory pathways governing mitoribosome homeostasis. The first is a Pck1-dependent mitochondrial maintenance pathway, which is restored by empagliflozin and functions to preserve mitoribosome abundance during stress. The second is the Nmnat1–Hic1 axis [22], which regulates mitoribosome expansion and is not modulated by empagliflozin. Indeed, Nmnat1 deficiency has been shown to induce excessive mitoribosome accumulation through suppression of Hic1 (Figure S16). These findings suggest that empagliflozin primarily acts through the Pck1-dependent maintenance pathway, preserving mitoribosome integrity during injury without promoting excessive organelle expansion under basal conditions.

Our renal findings are consistent with emerging evidence demonstrating ketone-dependent protective effects of empagliflozin in other organs following cardiac arrest. In a rat model of global cerebral ischemia, empagliflozin improved neurological outcomes, increased serum and brain BHB levels, reduced neuroinflammation, and preserved mitochondrial structure and complex I activity; these effects were abolished by inhibition of ketone oxidation [23]. Our study extends these observations by identifying a kidney-specific downstream mechanism involving Pck1-dependent preservation of mitochondrial translation.

Interpretation of ketone body dynamics after CA/CPR requires careful consideration. Circulating ketone levels are influenced by multiple factors, including fasting status, glucose and insulin administration, vasopressor use, temperature management, and metabolic redistribution during rewarming. Early increases in ketone levels may reflect acute stress responses and enhanced substrate utilization, whereas prolonged reductions may result from hepatic ischemia and systemic metabolic dysregulation [24]. Moreover, circulating BHB levels do not necessarily reflect total ketone availability, and tissue uptake, particularly in the brain, may not parallel plasma concentrations. Hypothermia-induced metabolic suppression and rewarming-associated metabolic shifts further complicate interpretation [25]. Current post-resuscitation management guidelines emphasize strict systemic and temperature control [26,27], underscoring the importance of interpreting metabolic markers within the broader physiological context.

Our findings also provide mechanistic insight into emerging clinical observations regarding SGLT2 inhibitors. Retrospective studies have suggested that SGLT2 inhibitors may reduce multiorgan injury after cardiac arrest and improve outcomes in patients with left ventricular assist devices [27]. Although these studies did not specifically evaluate post-resuscitation organ protection, they support the concept that SGLT2 inhibitors enhance systemic resilience under severe hemodynamic stress. Our data provide a potential biological explanation for these effects by demonstrating that SGLT2 inhibition preserves a ketone-dependent organelle maintenance pathway that is essential for cellular survival during ischemic injury.

Several limitations of this study should be acknowledged. First, no clinical trials have evaluated SGLT2 inhibitors for prevention of post-resuscitation organ injury, and their efficacy during the acute phase after cardiac arrest remains uncertain. Second, the applicability of SGLT2 inhibitors in patients with advanced renal dysfunction requires further investigation. Third, empagliflozin was administered before CA/CPR in our experimental model, whereas cardiac arrest in humans occurs unpredictably; therefore, whether administration after ROSC provides similar protection remains unknown. In addition, other pathways, including NAD+ metabolism, peroxisomal redox regulation, and mitochondrial dynamics, may also contribute to tubular resilience during ischemic stress.

To validate the appropriateness of comparing metabolic and molecular phenotypes across experimental conditions, we additionally assessed serum creatinine levels as a functional marker of AKI (Figure S17). Figure S17A demonstrated that all AKI models, except saline and sham controls, exhibited comparable elevations in serum creatinine, indicating similar degrees of renal dysfunction across models and supporting the validity of comparing circulating BHB levels under comparable AKI severity. Figure S17B further demonstrated that CA/CPR induced a clear time-dependent increase in serum creatinine, consistent with progressive renal injury in this model. In Figure S17C, Pck1 CKO mice subjected to short-duration CA/CPR exhibited greater increases in serum creatinine than control mice, demonstrating that Pck1 deficiency exacerbates susceptibility to CA/CPR-induced renal injury. Importantly, short-duration CA/CPR alone caused only minimal creatinine elevation, indicating that this protocol served as an appropriate sensitizing condition for revealing the Pck1-dependent phenotype without inducing overwhelming AKI that could confound mechanistic interpretation. Finally, Figure S17D demonstrated that empagliflozin significantly attenuated CA/CPR-induced creatinine elevation in the four-group comparison, consistent with its protective effects on tubular injury and mitochondrial–peroxisomal integrity. Collectively, these findings support the robustness of our mechanistic conclusions by confirming that AKI severity was appropriately controlled across experimental conditions.

Empagliflozin increased circulating BHB levels not only after CA/CPR but also in Sham mice. This finding reflects a well-recognized physiological effect of SGLT2 inhibition rather than a CA/CPR-specific response. SGLT2 inhibitors promote glucosuria and shift systemic metabolism toward enhanced fatty acid oxidation, resulting in mild ketogenesis even under non-injured conditions. Therefore, the elevation of BHB in Sham + Empa mice represents the baseline metabolic action of empagliflozin.

Importantly, CA/CPR caused a marked reduction in circulating BHB, consistent with metabolic collapse and impaired mitochondrial function. Empagliflozin restored BHB levels in CA/CPR mice to those observed in Sham controls, indicating preservation of mitochondrial ketone metabolism under severe ischemic stress. Thus, the key implication of Figure 4B is that empagliflozin prevents CA/CPR-induced depletion of BHB and maintains the BHB–C/EBPβ–Pck1 axis, which is essential for sustaining mitoribosomal integrity and OXPHOS capacity during acute kidney injury.

The functional experiments provide mechanistic support for the conclusion that Pck1 deficiency impairs mitochondrial and peroxisomal homeostasis, and that empagliflozin preserves these metabolic functions following cardiac arrest and cardiopulmonary resuscitation (CA/CPR). These Supplementary Materials extend the main results by demonstrating direct functional consequences of mitoribosomal disruption and its rescue by empagliflozin.

In Pck1 conditional knockout (CKO) mice, mitochondrial respiration was markedly impaired (Figure S18A). CKO tubular epithelial cells exhibited reduced basal and maximal oxygen consumption rates (OCR), loss of mitochondrial membrane potential (Figure S18B), increased mitochondrial ROS (Figure S18C), and decreased ATP production (Figure S18D). These functional abnormalities are consistent with the observed reductions in CI, CIII, CIV, and CV activities (Figure S19), all of which require mtDNA-encoded subunits synthesized by mitoribosomes. The selective increase in CII activity (Figure S19) likely reflects compensatory upregulation of the nDNA-encoded complex. Together, these findings demonstrate that Pck1 deficiency disrupts mitoribosomal function, leading to impaired OXPHOS capacity and increased oxidative stress.

Peroxisomal dysfunction was also evident in CKO kidneys, as shown by reduced catalase and ACOX1 activities (Figure S20). These results indicate impaired H_2_O_2_ detoxification and defective peroxisomal β-oxidation, suggesting that Pck1 deficiency affects both mitochondrial and peroxisomal metabolic pathways. The combined impairment of OXPHOS and peroxisomal FAO provides a mechanistic explanation for the severe energy depletion observed in CKO tubular epithelial cells.

In the CA/CPR model, empagliflozin significantly preserved mitochondrial function. Empagliflozin restored OCR (Figure S21A), mitochondrial membrane potential (Figure S21B), and ATP levels (Figure S21D), and suppressed mitochondrial ROS production (Figure S21C). Empagliflozin also rescued CI, CIII, CIV, and CV activities (Figure S22), indicating preservation of mitoribosomal translation of mtDNA-encoded OXPHOS subunits. These findings support the hypothesis that empagliflozin maintains mitochondrial translational capacity through the BHB–C/EBPβ–Pck1 axis. Furthermore, empagliflozin restored catalase and ACOX1 activities following CA/CPR (Figure S23), demonstrating protection of peroxisomal function and suggesting coordinated preservation of mitochondrial–peroxisomal metabolic networks.

Finally, the extensive positive and negative immunofluorescence controls (Figures S24–S33) confirm the specificity of all antibodies used in the main and Supplementary Materials. Immune-depletion controls abolished all specific signals, validating the reliability of the immunofluorescence data. For TUNEL staining, omission of the TdT enzyme eliminated nuclear labeling (Figure S26). These controls strengthen the interpretation of mitochondrial and peroxisomal protein expression patterns.

Collectively, these Supplementary Data provide comprehensive functional evidence that Pck1 is essential for maintaining mitochondrial ribosomal integrity, OXPHOS activity, and peroxisomal metabolism. They further demonstrate that empagliflozin protects renal metabolic function after CA/CPR by preserving mitoribosomal translation and peroxisomal enzymatic activity. These findings reinforce the mechanistic framework proposed in the main manuscript and substantiate the role of the BHB-dependent Pck1–mitoribosome axis in renal protection.

In conclusion, our findings demonstrate that empagliflozin functions as a metabolic stabilizer that preserves organelle homeostasis by maintaining the BHB–C/EBPβ–Pck1 axis during ischemic injury. By restoring ketone-dependent transcriptional signaling and sustaining mitochondrial translation, empagliflozin reinforces intrinsic metabolic defense mechanisms in proximal tubular cells during severe ischemic stress. This context-dependent mechanism distinguishes empagliflozin from agents that directly induce organelle biogenesis and highlights its role in preserving mitochondrial and peroxisomal resilience when endogenous ketone production and Pck1 expression are compromised.

## 4. Material and Methods

### 4.1. Animals

Male C57BL/6J mice (12 weeks old) were housed in a specific pathogen-free facility under controlled conditions (22–24 °C, 55–65% relative humidity, 12-h light/dark cycle) with free access to standard chow and water. All experimental procedures were approved by the Institutional Animal Care and Use Committee and were performed in accordance with international guidelines for laboratory animal welfare and the animal experimentation guidelines of the Tokushima University School of Medicine. This study was conducted under the approval of the Tokushima University Animal Care and Use Committee (Approval No. T2023-89).

Before all surgical procedures, mice were anesthetized with isoflurane (2–4% for induction and 1.2–1.5% for maintenance). At the end of each experiment, mice were euthanized by cervical dislocation under deep anesthesia. A total of 7 mice per group were used for all CA/CPR experiments, histological analyses, immunofluorescence staining, and biochemical assays unless otherwise specified.

### 4.2. CA/CPR Model

A mouse model of cardiac arrest/cardiopulmonary resuscitation (CA/CPR) was established in 12-week-old male C57BL/6J mice using previously validated protocols with minor modifications. Mice were anesthetized, placed in the supine position on a heating pad, endotracheally intubated, and mechanically ventilated. A jugular venous catheter was inserted for intravenous administration of KCl and epinephrine. Body temperature was continuously monitored using a rectal probe and maintained at 37 °C throughout the procedure.

Cardiac arrest was induced by intravenous injection of 50 μL of 0.5 M KCl, followed immediately by cessation of mechanical ventilation. Asystole was confirmed by electrocardiography. Mechanical ventilation was withheld for 7.5 min to induce systemic ischemia. KCl (FUJIFILM Wako, Osaka, Japan) and epinephrine (Sigma-Aldrich, St. Louis, MI, USA) were used for induction of cardiac arrest and resuscitation, respectively.

Resuscitation was initiated by restarting mechanical ventilation with oxygenated gas and performing manual chest compressions at a rate of 300 compressions/min over the lateral thoracic wall. Epinephrine (16 μg) was administered intravenously over 30–60 s during resuscitation. ROSC was confirmed by sustained electrocardiographic activity.

Mechanical ventilation was continued until spontaneous respiration exceeded 40 breaths/min, typically within 10–15 min after ROSC. Mice were then extubated, and the venous catheter and rectal probe were removed. Animals were transferred to a recovery cage maintained at 37 °C for 2 h before being returned to their home cages.

Sham-operated mice underwent anesthesia, intubation, temperature monitoring, and electrocardiographic recording for equivalent durations but did not undergo venous catheterization, KCl injection, or chest compressions.

Kidneys were harvested at 0, 6, and 24 h after ROSC for molecular and histological analyses, including quantitative PCR analysis of C/EBPβ and Pck1 and immunofluorescence staining of Pck1 using LTL as a proximal tubular marker.

For each time point (0, 6, and 24 h), 7 mice per group were analyzed.

### 4.3. Renal Proximal Tubule-Specific Pck1 Knockout Mice and Short-Duration CA/CPR Protocol

Renal proximal tubule-specific Pck1 knockout (Pck1 CKO; Pck1^flox/flox/gGT-Cre^tg/−) mice were generated as previously described [8]. Briefly, Pck1^flox/flox^ mice on a C57BL/6J background were crossed with γ-glutamyl transpeptidase (gGT)-Cre transgenic mice (Jackson Laboratory). This breeding strategy generated three control genotypes (Pck1^flox/flox^, Pck1^flox/−^, and Pck1^flox/−/gGT-Cre^tg/−). Pck1^flox/−/gGT-Cre^tg/− mice were subsequently crossed with Pck1^flox/flox^ mice to generate Pck1^flox/flox/gGT-Cre^tg/− mice, which were used as Pck1 CKO mice. Pck1^flox/flox^ littermates served as controls.

Twelve-week-old male Pck1 CKO and control mice were subjected to a modified short-duration CA/CPR protocol. The procedure was identical to the standard CA/CPR protocol except that the duration of cardiac arrest was reduced to 3 min. After the ischemic period, resuscitation and post-resuscitation care were performed as described for the standard protocol. Mice were euthanized 24 h after short-duration CA/CPR for renal and urinary analyses.

For histological analyses, kidneys were fixed in 4% paraformaldehyde, embedded in paraffin, and sectioned. PAS was performed to evaluate tubular injury, and tubular injury scores were assessed in a blinded manner.

A total of 7 mice per genotype (Pck1 CKO and control) were used for short-duration CA/CPR experiments and subsequent analyses.

Paraformaldehyde (FUJIFILM Wako, Japan), paraffin embedding reagents (Sakura Finetek, Tokyo, Japan), and PAS reagents (Sigma-Aldrich, USA) were obtained from the indicated suppliers.

### 4.4. Immunofluorescence Staining

Immunofluorescence staining was performed on 5-μm cryostat kidney sections. After fixation and blocking, sections were incubated overnight at 4 °C with primary antibodies against Pck1 (1:500, ab28455, Abcam, Cambridge, MA, USA), PMP70 (1:500, PA1-650, Millipore, Bedford, MA, USA), catalase (1:1000, ab52477, Abcam), ACOX1 (68017-1-Ig, Proteintech, Chicago, IL, USA), PGC-1α (1:250, 66369-1-Ig, Proteintech), MCAD (1:500, ab92461, Abcam), 4-HNE (1:100, HNEJ-2, Japan Institute for the Control of Aging, Shizuoka, Japan), and AQP1 (1:100, B-11, Santa Cruz Biotechnology, Santa Cruz, CA, USA). Proximal tubules were identified using biotinylated LTL (1:500, L-132, Vector Laboratories).

After washing, sections were incubated with fluorophore-conjugated secondary antibodies (Jackson ImmunoResearch Laboratories, West Grove, PA, USA), and nuclei were counterstained with DAPI (1:1000, Sigma-Aldrich, USA).

To identify apoptotic proximal tubular cells, dual TUNEL/AQP1 immunofluorescence staining was performed. TUNEL staining was conducted using a commercial kit (Roche Diagnostics, Basel, Switzerland) according to the manufacturer’s instructions, followed by AQP1 immunolabeling.

Fluorescence images were acquired using identical exposure settings for all experimental groups. Quantitative analyses were performed in LTL-positive proximal tubular regions using standardized regions of interest.

### 4.5. Immunohistochemistry

Immunohistochemistry was performed on paraffin-embedded kidney sections to evaluate mitochondrial ribosome abundance and mitochondrial translational activity. Following deparaffinization, rehydration, and antigen retrieval, sections were incubated with primary antibodies against MRPL13 (1:200, P0A5-103516, Thermo Fisher Scientific, Waltham, MA, USA) and MRPS15 (1:200, PA5-103517, Thermo Fisher Scientific) as markers of mitochondrial ribosome abundance. Additional sections were incubated with antibodies against ND1 (1:100, ab181848, Abcam, Cambridge, UK) and COX1 (1:100, ab14705, Abcam), which are mtDNA-encoded OXPHOS subunits translated exclusively by mitochondrial ribosomes and therefore serve as indicators of mitochondrial translational activity.

After incubation with horseradish peroxidase (HRP)-conjugated secondary antibodies, immunoreactivity was visualized using a DAB substrate. Sections were then counterstained with hematoxylin, dehydrated, and mounted. Images were captured using a 3CCD digital camera system under identical exposure conditions across all groups. Positively stained cortical areas were quantified as arbitrary units using standardized image analysis protocols.

HRP-conjugated secondary antibodies (Vector Laboratories, Newark, CA, USA), DAB substrate (FUJIFILM Wako, Japan), and hematoxylin (Sigma-Aldrich, USA) were obtained from the indicated suppliers.

### 4.6. Electron Microscopy

Kidney specimens were fixed and embedded in Epon epoxy resin (Hexion, Columbus, OH, USA). Electron micrographs of 10 randomly selected proximal tubular cells per kidney were obtained for morphometric analysis.

### 4.7. RNA Isolation, Reverse Transcription, and Quantitative PCR

Total RNA was isolated from cultured cells and kidney tissues using the RNeasy Plus Mini Kit (QIAGEN, Hilden, Germany). Quantitative real-time PCR was performed using the ABI Prism 7700 Sequence Detection System with SYBR Green reagents (Applied Biosystems, Foster City, CA, USA). cDNA was synthesized from 1 µg of total RNA using reverse transcriptase according to the manufacturer’s instructions.

Relative mRNA expression was quantified using the ΔΔCt method, following MIQE guidelines. Glyceraldehyde-3-phosphate dehydrogenase (GAPDH) was used as the internal reference gene because its Ct values were stable across all experimental groups. All reactions were performed in triplicate, and amplification specificity was confirmed by melt-curve analysis.

Primer sequences were as follows: Pck1: forward, 5′-AAGTGCCTGCACTCTGTGG-3′; reverse, 5′-CAGGCCCAGTTGTTGACC-3′; C/EBPβ: forward, 5′-AGCGGCTGCAGAAGAAGTAT-3′; reverse, 5′-CTGCTTGAACAAGTTCCGC-3′; and GAPDH: forward, 5′-CCAGGGCTGCTTTTAACTC-3′; reverse, 5′-GCTCCCCCCTGCAAATGA-3′.

qPCR cycling conditions were as follows: initial denaturation at 95 °C for 10 min, followed by 40 cycles of denaturation at 95 °C for 15 s, annealing at 60 °C for 30 s, and extension at 72 °C for 30 s. Melt curve analysis was performed from 65 °C to 95 °C with 0.5 °C increments.

### 4.8. Empagliflozin Administration and Experimental Design

Empagliflozin administration was performed according to previously published protocols. Eight-week-old male C57BL/6J mice (CLEA, Tokyo, Japan) received once-daily oral gavage of either vehicle (0.5% methylcellulose) or empagliflozin (10 mg/kg; Boehringer Ingelheim, Ingelheim am Rhein, Germany) suspended in 0.5% methylcellulose for seven consecutive days.

After 1 week of treatment, 9-week-old mice were randomly assigned to four groups (*n* = 7/group): Vehicle + Sham, Empagliflozin + Sham, Vehicle + CA/CPR, and Empagliflozin + CA/CPR.

CA/CPR was induced using the standard 7.5-min cardiac arrest protocol, whereas sham-operated mice underwent anesthesia, intubation, and monitoring without KCl injection or chest compressions. All mice were euthanized 24 h after CA/CPR or sham procedures for biochemical and histological analyses.

Outcome measurements included plasma BHB levels, renal C/EBPβ mRNA expression assessed by quantitative PCR, and immunofluorescence staining of Pck1 using LTL as a proximal tubular marker.

Renal injury was evaluated on Periodic Acid–Schiff (PAS)-stained kidney sections. Cortical tubular damage was scored in a blinded manner using a semi-quantitative scale based on the percentage of affected tubules in each field:

Score 0: No detectable tubular injury.
Score 1: Mild injury involving <25% of tubules (loss of brush border, mild epithelial flattening, occasional luminal debris).
Score 2: Moderate injury involving 25–50% of tubules (obvious epithelial flattening, luminal PAS-positive casts, focal tubular dilation).
Score 3: Severe injury involving 50–75% of tubules (widespread epithelial necrosis, prominent PAS-positive casts, marked dilation).
Score 4: Very severe injury involving >75% of tubules (diffuse necrosis, extensive PAS-positive casts, tubular collapse or disappearance).

For each mouse, 7 non-overlapping cortical fields were analyzed at ×100 magnification, and the mean tubular injury score was calculated. All scoring was performed by an observer blinded to the experimental groups.

### 4.9. Urine Collection and Analysis

Urine samples were collected from mice in all four experimental groups (Vehicle + Sham, Empagliflozin + Sham, Vehicle + CA/CPR, and Empagliflozin + CA/CPR) 24 h after short-duration CA/CPR or sham procedures.

Fresh urine samples were centrifuged at 3000× *g* for 10 min to remove debris. Supernatants were mixed with Laemmli sample buffer, and equal urine volumes were loaded onto 15% SDS–polyacrylamide gels under reducing conditions. Following electrophoresis, gels were stained with Coomassie Brilliant Blue to visualize urinary protein profiles, particularly albumin-range bands.

Band intensities were quantified by densitometric analysis using standardized parameters across all groups.

Laemmli sample buffer (Bio-Rad, Hercules, CA, USA), SDS–PAGE reagents (Bio-Rad, USA), and Coomassie Brilliant Blue (FUJIFILM Wako, Japan) were used for urine protein analysis.

### 4.10. Mitochondrial Function

Mitochondrial function was assessed using isolated primary kidney epithelial cells from each group of mice. Tubular epithelial cells were isolated as described previously [8]. Endogenous cellular oxygen consumption rate (OCR) was measured using an XF-24 extracellular flux analyzer (Seahorse Bioscience, Santa Clara, CA, USA) according to the manufacturer’s protocol.

Mitochondrial membrane potential was evaluated using the JC-1 mitochondrial membrane potential detection kit (Peninsula Laboratories, Inc., San Carlos, CA, USA), following the manufacturer’s instructions. Mitochondrial reactive oxygen species (ROS) levels were determined by staining harvested cells with MitoSOX Red (Molecular Probes, Eugene, OR, USA). ATP content was subsequently measured as described previously [8].

### 4.11. OXPHOS Complex Activities

Kidney mitochondria were isolated using differential centrifugation as described previously [8]. Enzyme activities of mitochondrial Complex I (CI), Complex II (CII), Complex III (CIII), Complex IV (CIV), and Complex V (CV) were measured at room temperature using a Beckman Coulter DU 530 Spectrophotometer (Beckman Coulter, Brea, CA, USA), following previously published protocols [3]. Citrate synthase activity was measured at 412 nm (ε = 13.6 mM^−1^ cm^−1^) and used to normalize mitochondrial protein content.

Rotenone-sensitive CI, malonate-sensitive CII, antimycin A-sensitive (AA) CIII, KCN-sensitive CIV, and oligomycin-sensitive CV activities were quantified. All activity results represent the average of seven independent assays performed using pooled mitochondrial samples from each group of mice.

### 4.12. Catalase and ACOX1 Enzyme Activities

Catalase activity was determined using a colorimetric activity assay kit (Invitrogen, Carlsbad, CA, USA). ACOX1 activity was measured using an ELISA-based assay kit (ProteinTech Group, Inc., Chicago, IL, USA). All assays were performed strictly according to the manufacturers’ protocols.

### 4.13. Controls for Immunofluorescence Experiments

Positive and negative controls were used to validate each primary antibody. As positive controls, kidneys from 9-week-old healthy wild-type mice were used, because these kidneys physiologically express all target molecules examined. For 4-HNE immunofluorescence, kidneys from 15-month-old wild-type mice were used, as aging kidneys are known to exhibit strong 4-HNE staining.

Negative controls were generated by immune depletion. Each primary antibody was incubated with 5 mg/mL of its corresponding antigen solution at a 1:2 (*v*:*v*) ratio at 4 °C overnight. The pre-blocked antibody was then used as the primary antibody under identical staining conditions as the experimental samples. For the TUNEL negative control, the TdT enzyme was omitted from the reaction mixture.

### 4.14. Statistical Analysis

All statistical analyses were performed using Prism 8 software (GraphPad Software, San Diego, CA, USA). Sample sizes were determined based on power calculations while adhering to the principles of reduction, refinement, and replacement (3Rs). Data are presented as mean ± standard error of the mean (SEM). Group comparisons were performed using appropriate statistical tests as indicated in the corresponding figure legends.

Data distribution was assessed using the Shapiro–Wilk normality test. For normally distributed data, two-group comparisons were performed using unpaired two-tailed Student’s *t*-tests, and multiple-group comparisons were analyzed using one-way or two-way ANOVA followed by Tukey’s post hoc test. For non-normally distributed data, non-parametric tests were applied as appropriate. Statistical significance was defined as *p* < 0.05.

## 5. Conclusions

This study identifies the BHB–C/EBPβ–Pck1 axis as a central metabolic pathway that preserves mitoribosome integrity, maintains organelle homeostasis, and protects proximal tubular cells from CA/CPR-induced injury. Empagliflozin restores this ketone-dependent signaling pathway, prevents mitoribosome collapse, and confers multilevel organelle protection. These findings provide a mechanistic basis for exploring SGLT2 inhibitors as potential therapeutic agents for post-resuscitation AKI and identify Pck1 as a promising target for enhancing organelle resilience during severe ischemic stress.

## Data Availability

The original contributions presented in this study are included in the article/Supplementary Materials. Further inquiries can be directed to the corresponding author.

## Supplementary Materials

The following supporting information can be downloaded at: https://www.mdpi.com/article/10.3390/ijms27146366/s1.

## Abbreviations

The following abbreviations are used in this manuscript:

AKI	acute kidney injury
CA/CPR	cardiac arrest and cardiopulmonary resuscitation
CKO	conditional knockout
Cont	control
Empa	empagliflozin
TEC	tubular epithelial cell
OCR	oxygen consumption rate
OXPHOS	oxidative phosphorylation
ETC	electron transport chain
CI–CV	Complex I to Complex V
mtDNA	mitochondrial DNA
nDNA	nuclear DNA
ROS	reactive oxygen species
JC-1	5,5′,6,6′-Tetrachloro-1,1′,3,3′-tetraethylbenzimidazolylcarbocyanine iodide
MitoSOX	MitoSOX Red mitochondrial superoxide indicator
ATP	adenosine triphosphate
PAS	Periodic Acid–Schiff
HE	hematoxylin and eosin
LPF	low-power field
HPF	high-power field
IF	immunofluorescence
IHC	immunohistochemistry
DAB	3,3′-diaminobenzidine
HRP	horseradish peroxidase
PCK1	phosphoenolpyruvate carboxykinase 1
PMP70	peroxisomal membrane protein 70
ACOX1	acyl-CoA oxidase 1
MCAD	medium-chain acyl-CoA dehydrogenase
PGC1α	peroxisome proliferator-activated receptor gamma coactivator 1-alpha
AQP1	aquaporin-1
LTL	Lotus tetragonolobus lectin 4-HNE, 4-hydroxynonenal
TEM	transmission electron microscopy
DAPI	4′,6-diamidino-2-phenylindole
TUNEL	terminal deoxynucleotididyl transferase dUTP nick end labeling
