# Supplementary material for: Empagliflozin Prevents Cardiac Arrest-Induced Renal Injury Through BHB-Dependent Mitoribosome Maintenance
Source: Int J Mol Sci. 2026 Jul 17;27(14):6366. doi: 10.3390/ijms27146366 (PMC13411704; doi:10.3390/ijms27146366)
Supplement: Supplementary file 1 [file ijms-27-06366-s001.zip › ijms-4361967-supplementary.pdf]

Supplementary File

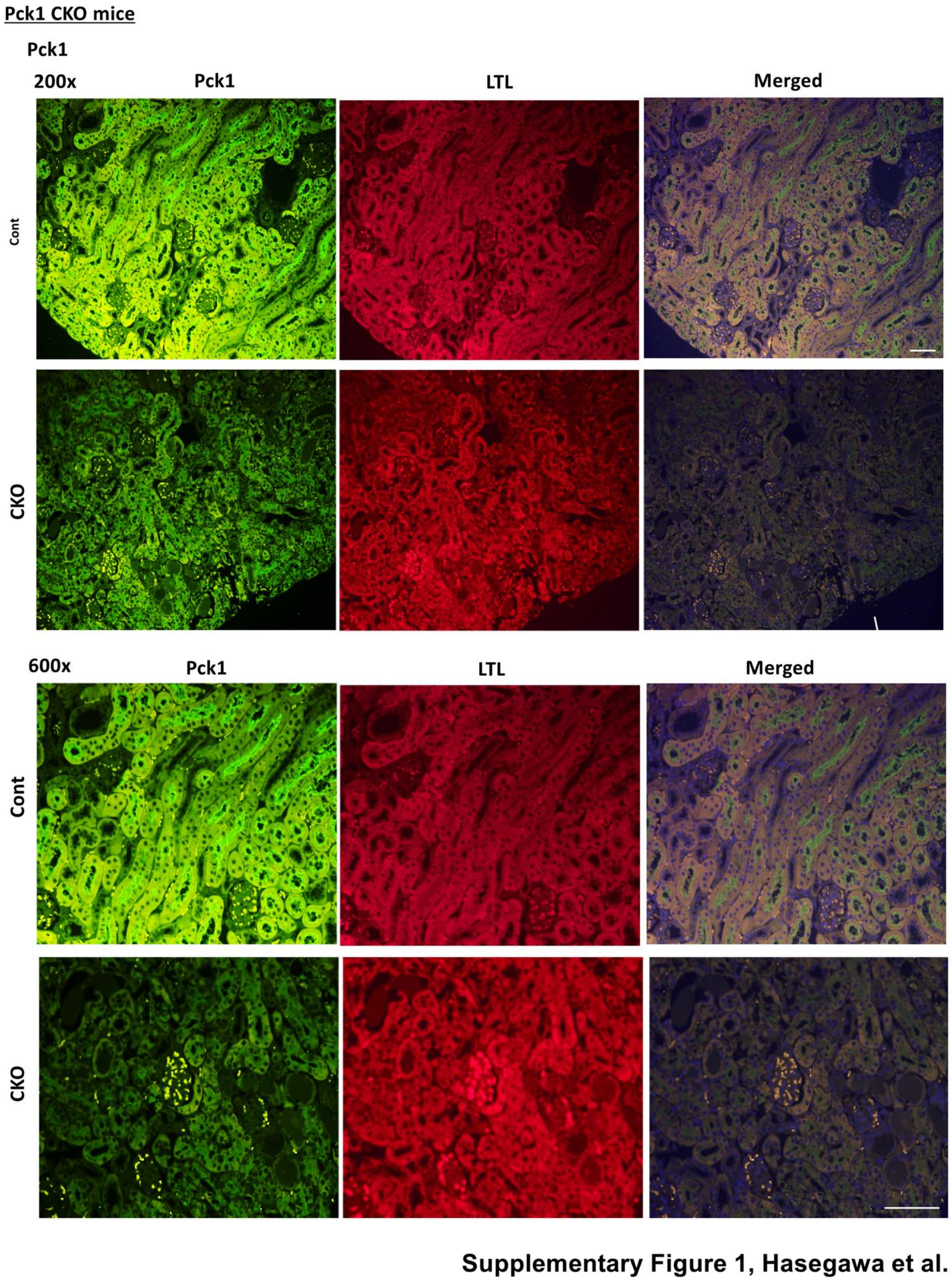

Figure S1. Pck1 immunofluorescence in Pck1 CKO mice after short-time CA/CPR. The low-magnification images (200×, upper panels) represent the original immunofluorescence images shown in Figure 1C from kidney sections of proximal tubule-specific Pck1 conditional knockout (CKO) and control (Cont) mice subjected to short-time CA/CPR at 9 weeks of age. These images are presented here at higher resolution because of space limitations in the main figure. Corresponding high-magnification images (600×, lower panels) are also shown. Kidney cryosections were stained with biotinylated LTL (red) as a proximal tubule marker and Pck1 (green). Representative photomicrographs demonstrate markedly reduced Pck1 expression in CKO mice compared with Cont mice (n = 7 mice/group; scale bar: 50 μm). Quantitative analyses are identical to those presented in Figure 1C.

CKO vs. Cont

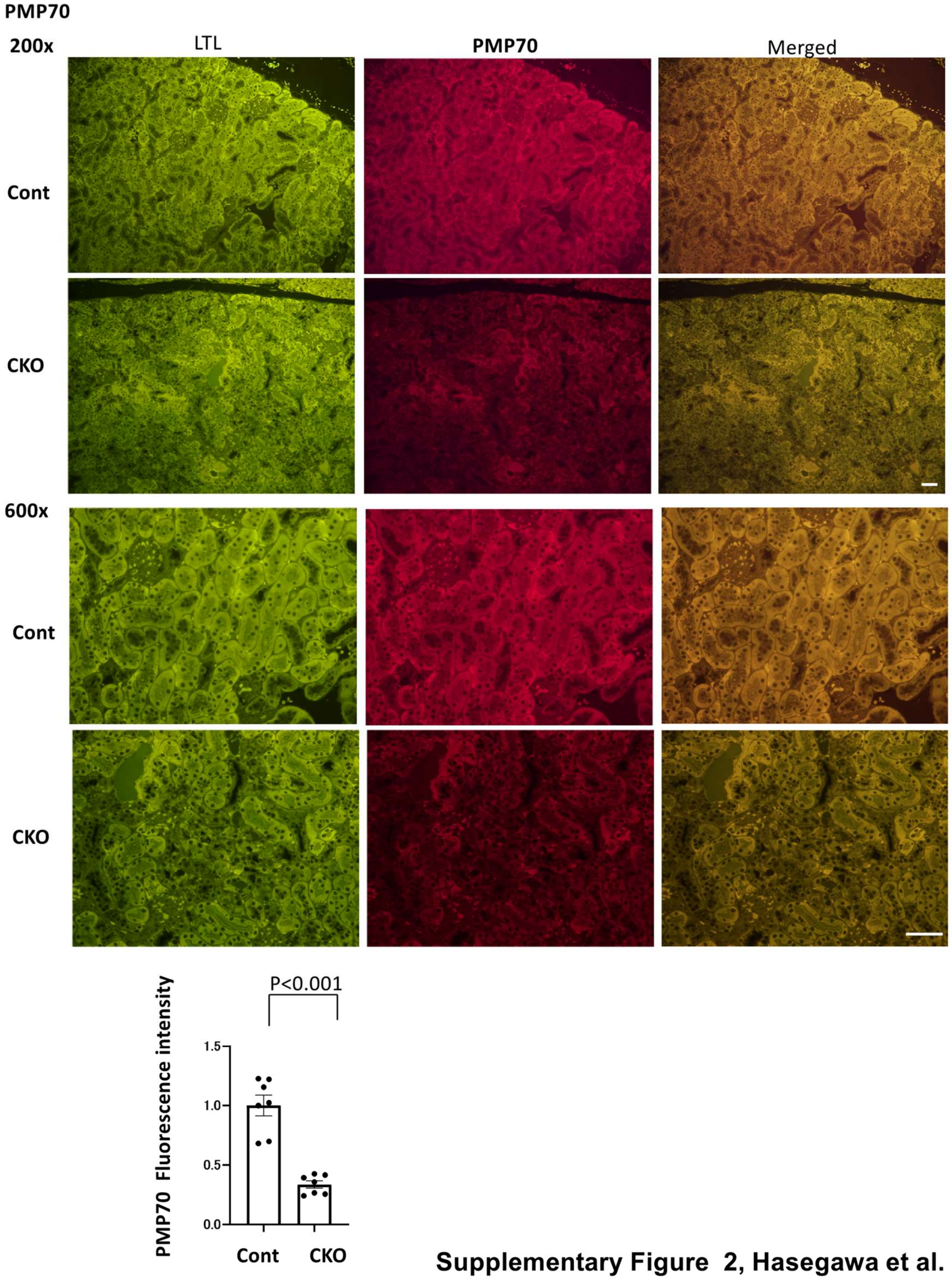

Supplementary Figure 2, Hasegawa et al.

CKO vs. Cont

Catalase

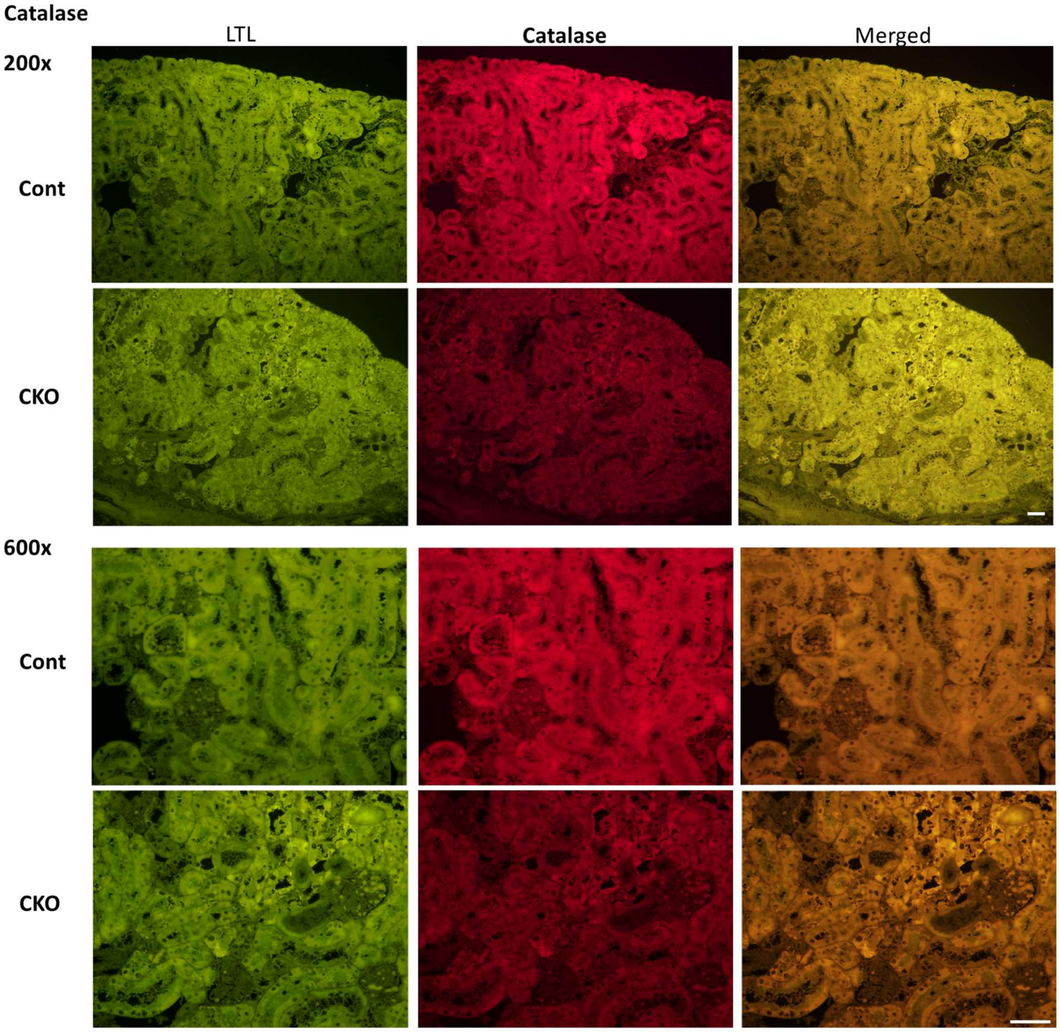

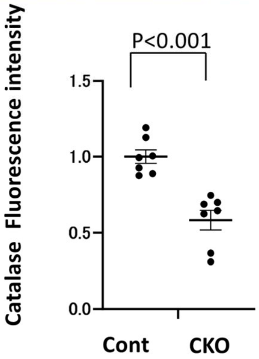

Supplementary Figure 3, Hasegawa et al.

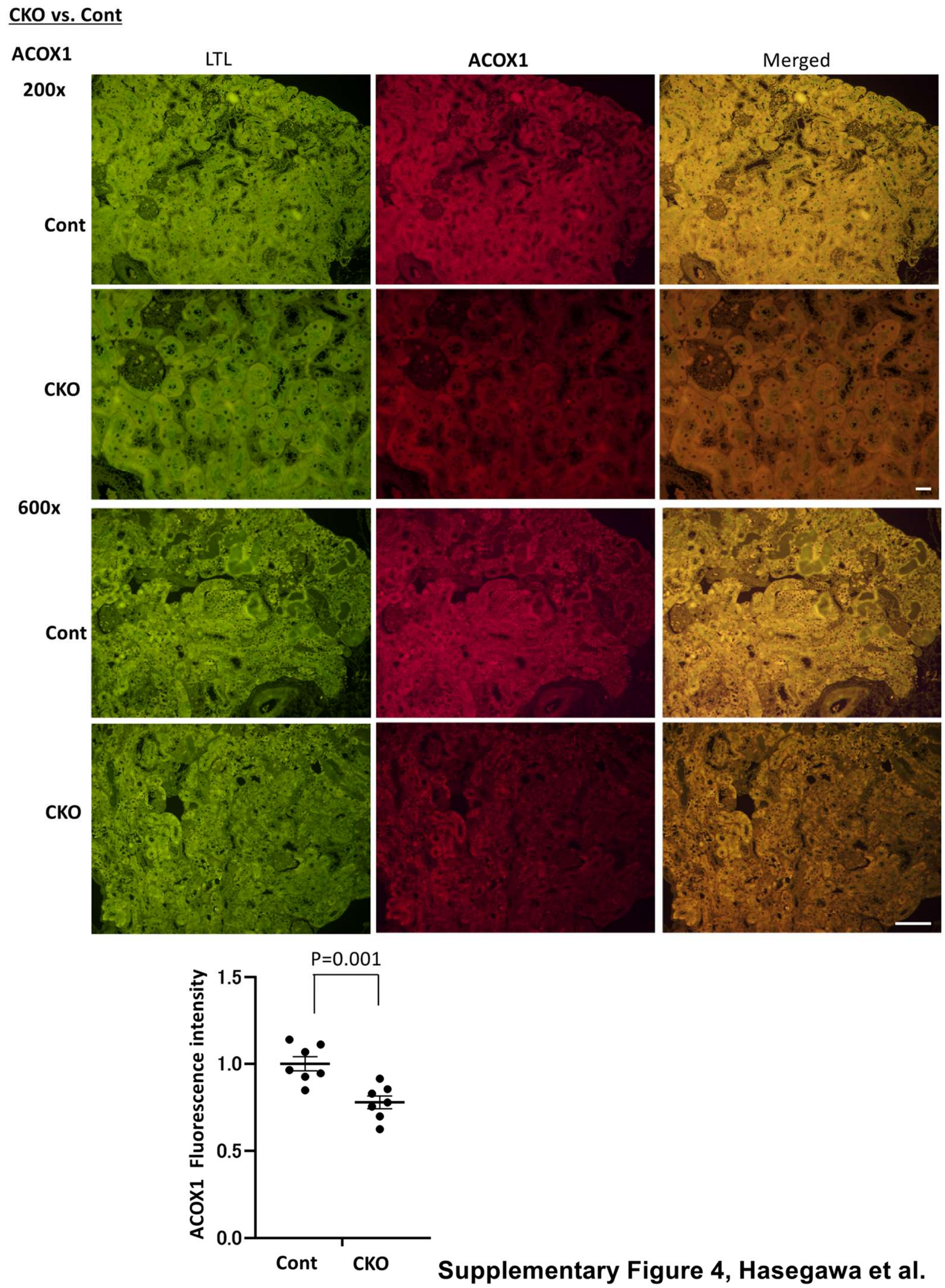

Supplementary Figure 4, Hasegawa et al.

Figures S2–S4. Peroxisomal marker immunofluorescence in Pck1 CKO mice subjected to short-time CA/CPR. Low-magnification (200×) and high-magnification (600×) immunofluorescence images of kidney sections from proximal tubule-specific Pck1 CKO and control (Cont) mice subjected to short-time CA/CPR at 9 weeks of age are shown. Kidney sections were stained with biotinylated LTL (green) as a proximal tubule marker, and peroxisomal markers were visualized in red. Supplementary Figure 2 shows PMP70 expression, Supplementary Figure 3 shows catalase expression, and Supplementary Figure 4 shows acyl-CoA oxidase 1 (ACOX1) expression. All three markers were markedly reduced in CKO mice compared with Cont mice (n = 7 mice/group; scale bar: 50 μm). The lower panels show quantitative fluorescence intensity analyses. For Supplementary Figure 2, the 600× images and quantitative analyses are identical to those shown in Figure 3A and are reproduced here at higher resolution to improve visual clarity. Statistical analyses were performed using a two-tailed Student's t-test.

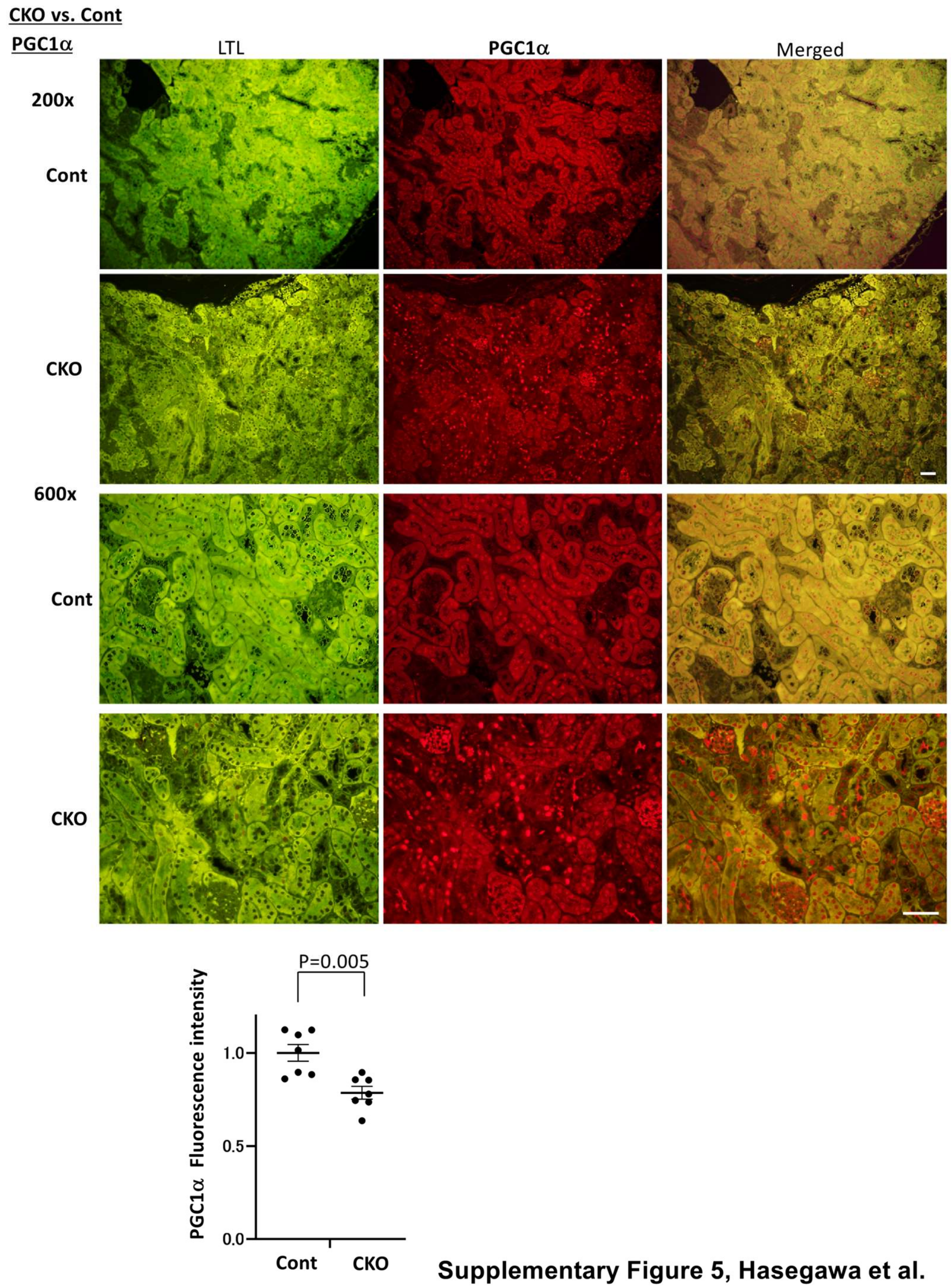

Supplementary Figure 5, Hasegawa et al.

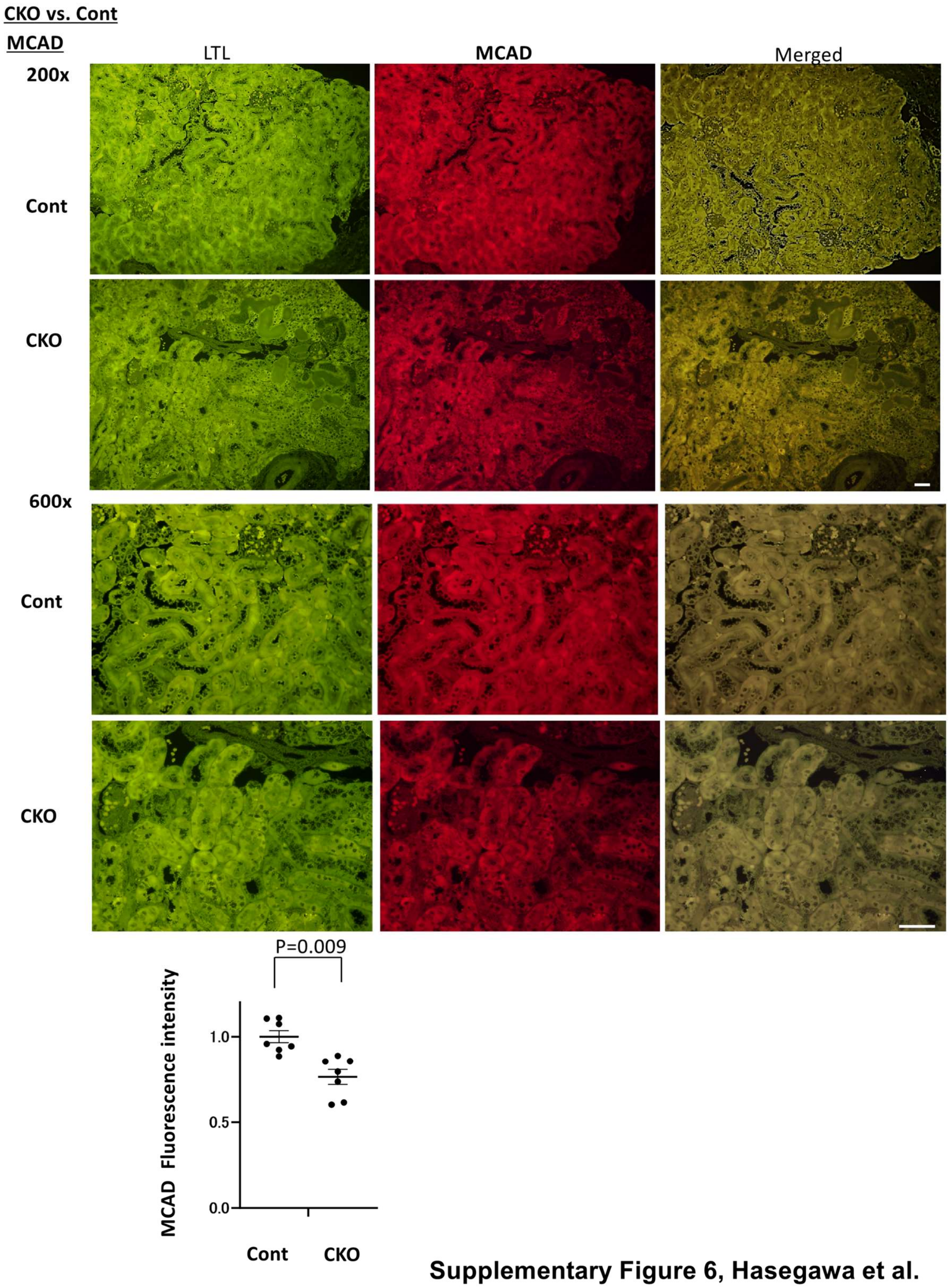

Supplementary Figure 6, Hasegawa et al.

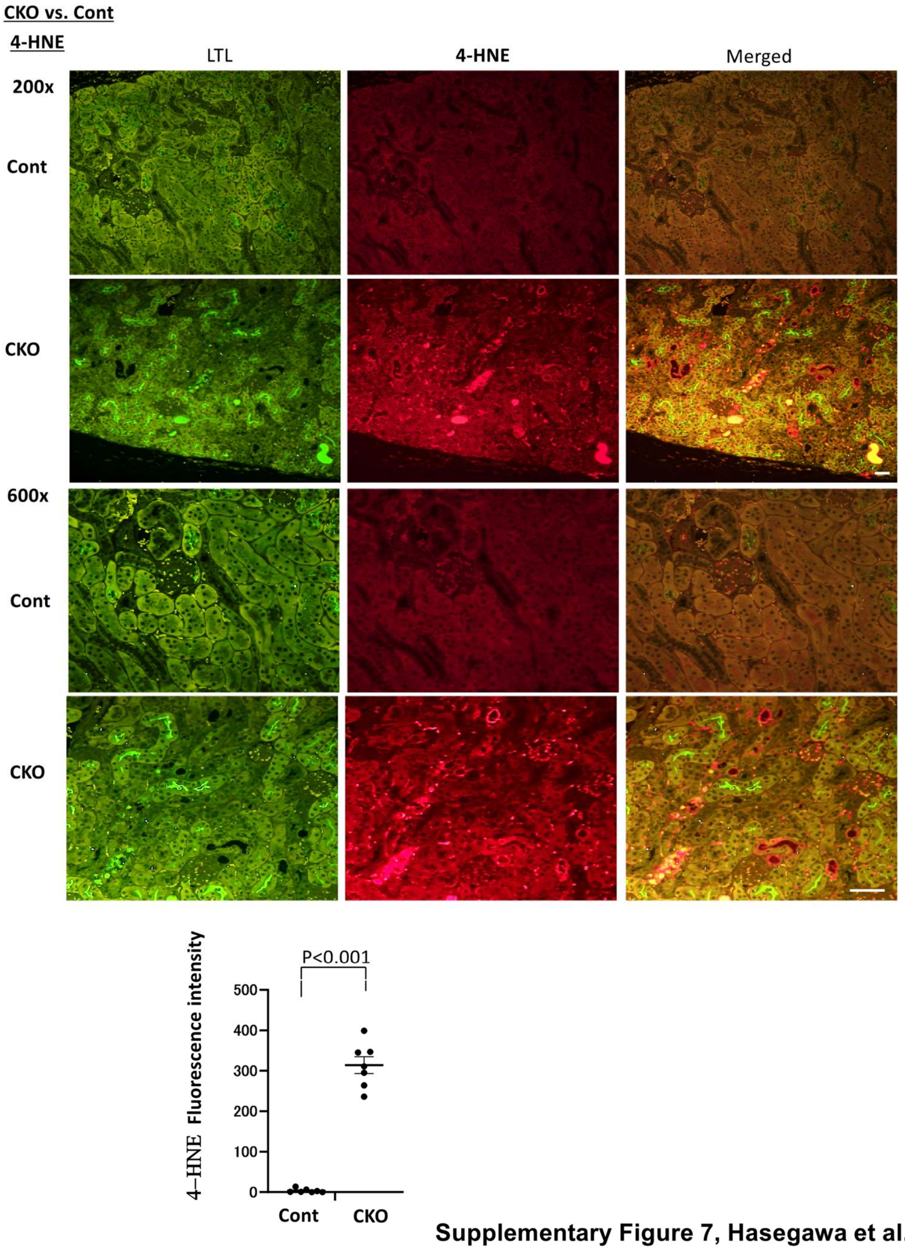

Supplementary Figure 7, Hasegawa et al.

Figures S5–S7. Mitochondrial markers and 4-HNE immunofluorescence in Pck1 CKO mice subjected to short-time CA/CPR. Low-magnification (200×) and high-magnification (600×) immunofluorescence images of kidney sections from proximal tubule-specific Pck1 CKO and control (Cont) mice subjected to short-time CA/CPR at 9 weeks of age are shown. Kidney sections were stained with biotinylated LTL (green) as a proximal tubule marker, and mitochondrial markers were visualized in red. Supplementary Figure 5 shows PGC-1α expression, and Supplementary Figure 6 shows medium-chain acyl-CoA dehydrogenase (MCAD) expression; both markers were reduced in CKO mice compared with Cont mice (n = 7 mice/group; scale bar: 50 μm). Supplementary Figure 7 shows immunostaining for 4-hydroxynonenal (4-HNE), indicating increased local reactive oxygen species (ROS) production predominantly in proximal tubules of CKO mice, whereas staining was less prominent in Cont mice (n = 7 mice/group; scale bar: 50 μm). The lower panels show quantitative fluorescence intensity analyses for each marker. Statistical analyses were performed using a two-tailed Student's t-test.

A. markers of mitochondrial ribosome number : **MRPL13 and MRPS15**

Marker	Mitochondrial Ribosomal Protein
MRPL13	Mitochondrial Ribosomal Protein Large Subunit
MRPS15	Mitochondrial Ribosomal Protein Small Subunit

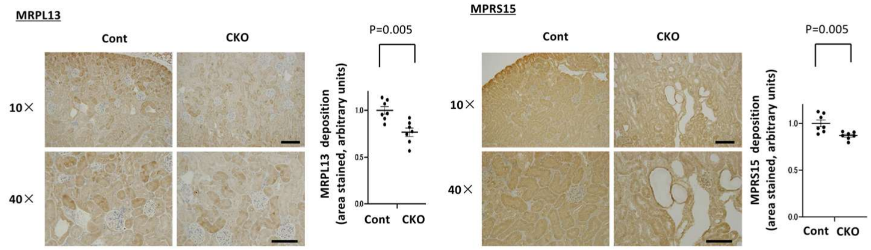

B. markers of mitochondrial ribosome function: **ND1 and COX1**

Marker	OXHPOS Complex	Encoded DNA	Protein synthesis (site)
NDUFA9	Complex I	Nuclear DNA(nDNA)	Cytoribosome
FP	Complex II	Nuclear DNA (nDNA)	Ctytoribosome
UQCRC2	Complex III	Nuclear DNA (nDNA)	Cytoribosome
COX1	Complex IV	Mitochondria DNA (mtDNA)	Mitoribosome
ATP5A1	Complex V	Nuclear DNA (nDNA)	Cytoribosome
ND1	Complex I	Mitochondria (mtDNA)	Mitoribosome

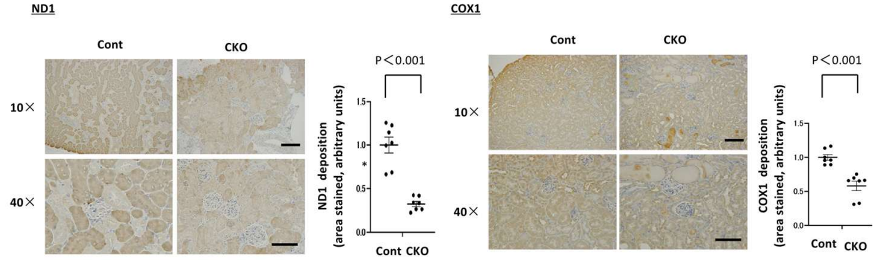

Supplementary Figure 8, Hasegawa et al.

Figure S8. Reduced mitoribosome abundance and impaired mitoribosomal function in Pck1 CKO mice subjected to short-time CA/CPR. (A) Immunohistochemical staining for the mitoribosomal proteins MRPL13 (mitochondrial ribosomal protein L13) and MRPS15 (mitochondrial ribosomal protein S15) was performed in kidney sections from proximal tubule-specific Pck1 CKO and control (Cont) mice subjected to short-time CA/CPR at 9 weeks of age. Both low-magnification (10×) and high-magnification (40×) images are shown. MRPL13 and MRPS15 represent large- and small-subunit mitoribosomal proteins, respectively, and together serve as markers of mitoribosome abundance. CKO mice exhibited markedly reduced MRPL13 and MRPS15 staining compared with Cont mice. Representative images and quantitative immunohistochemical scoring (mean ± SEM) are shown (n = 7 mice/group; scale bar: 50 μm). (B) To assess mitoribosomal function, immunostaining for ND1 and COX1, mtDNA-encoded OXPHOS subunits translated exclusively by mitoribosomes, was performed. Low-magnification (10×) and high-magnification (40×) images are shown. CKO mice exhibited significantly reduced ND1 and COX1 expression after short-time CA/CPR, indicating impaired mitoribosomal translation. Quantitative analyses are presented as mean ± SEM (n = 7 mice/group). Statistical analyses were performed using a two-tailed Student's t-test.

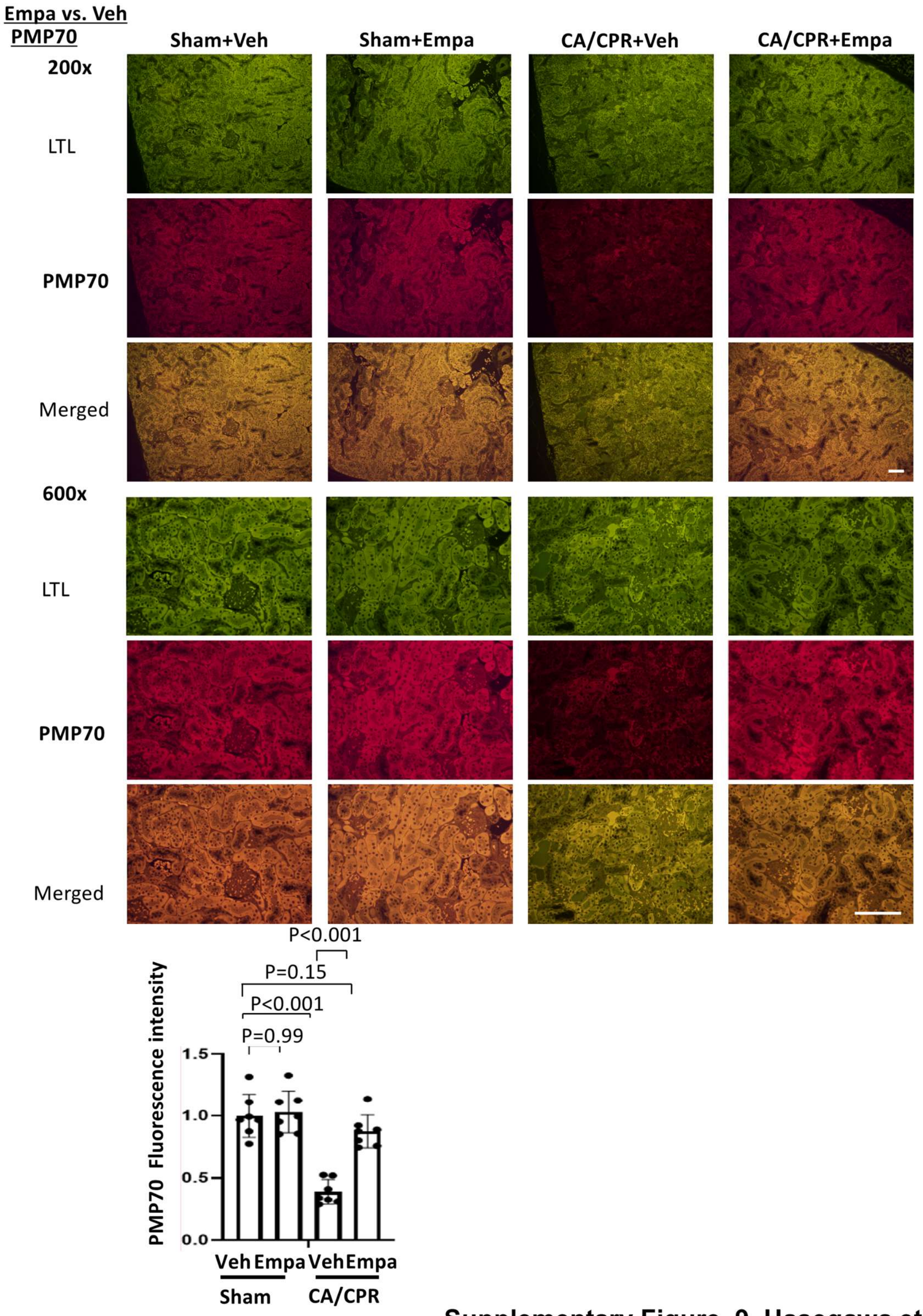

Supplementary Figure 9, Hasegawa et al.

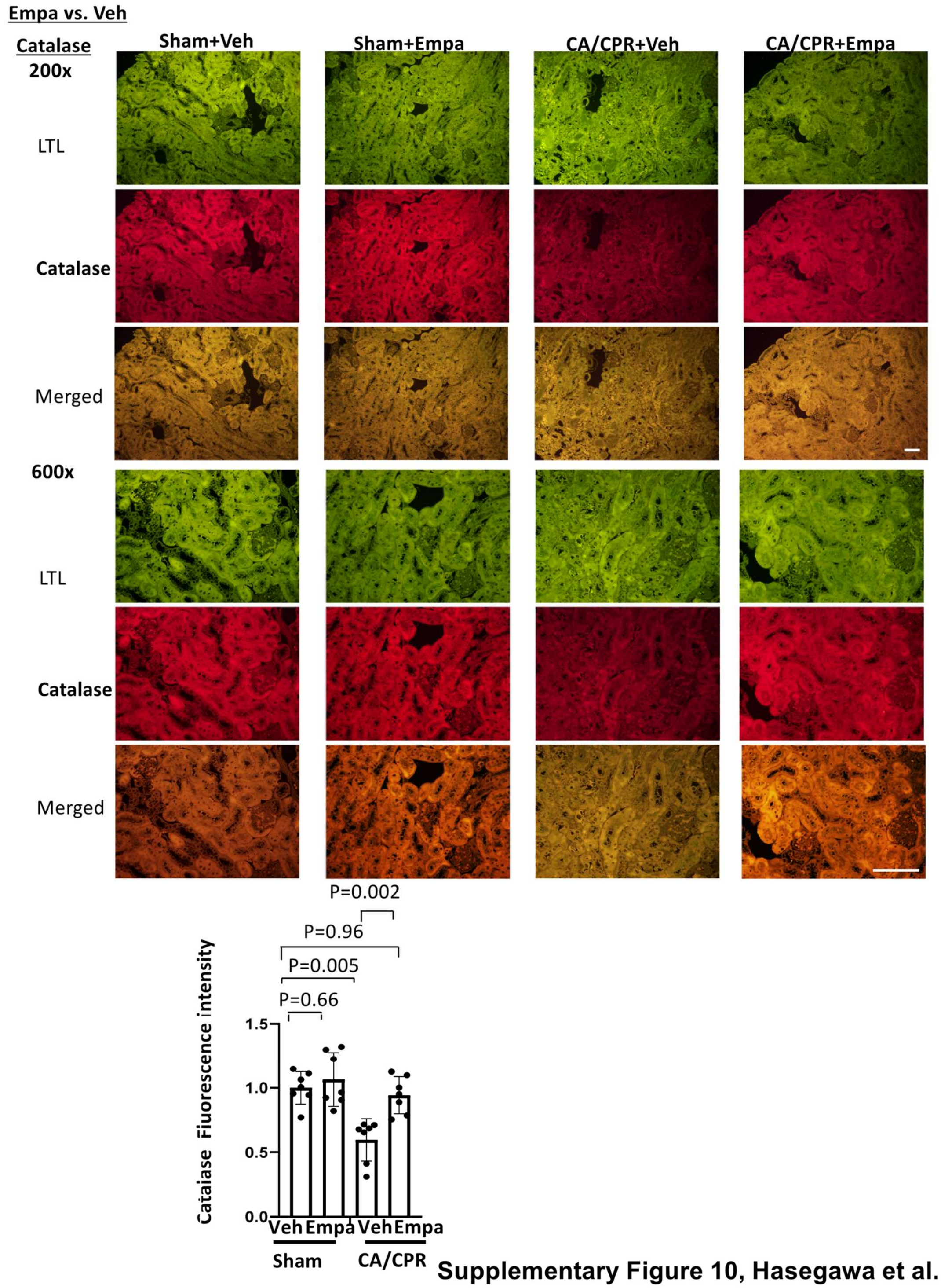

Supplementary Figure 10, Hasegawa et al.

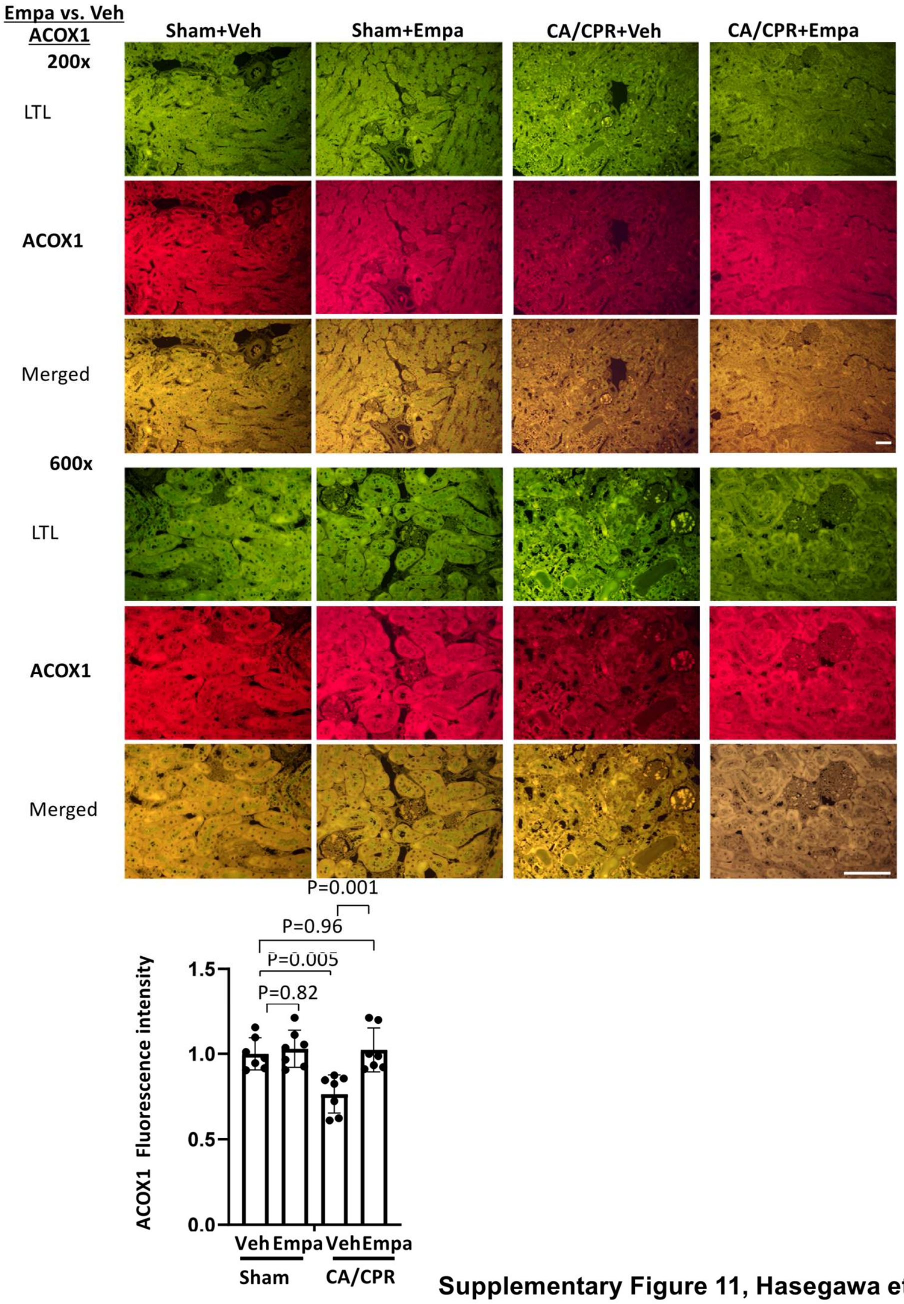

Supplementary Figure 11, Hasegawa et al.

Figures S9–S11. Peroxisomal marker immunofluorescence in vehicle- and empagliflozin-treated mice subjected to CA/CPR. Low-magnification (200×) and high-magnification (600×) immunofluorescence images of kidney sections from Veh + Sham, Empa + Sham, Veh + CA/CPR, and Empa + CA/CPR mice at 9 weeks of age are shown. Kidney sections were stained with biotinylated LTL (green) as a proximal tubule marker, and peroxisomal markers were visualized in red. Supplementary Figure 9 shows PMP70, a marker of peroxisome abundance, whereas Supplementary Figures 10 and 11 show catalase and acyl-CoA oxidase 1 (ACOX1), respectively, as markers of peroxisomal function. Veh + CA/CPR mice exhibited reduced expression of these peroxisomal markers, whereas Empa + CA/CPR mice showed preserved or restored expression. Representative images and quantitative fluorescence intensity analyses (mean ± SEM) are shown (n = 7 mice/group; scale bar: 50 μm). Statistical analyses were performed using a two-tailed Student's t-test.

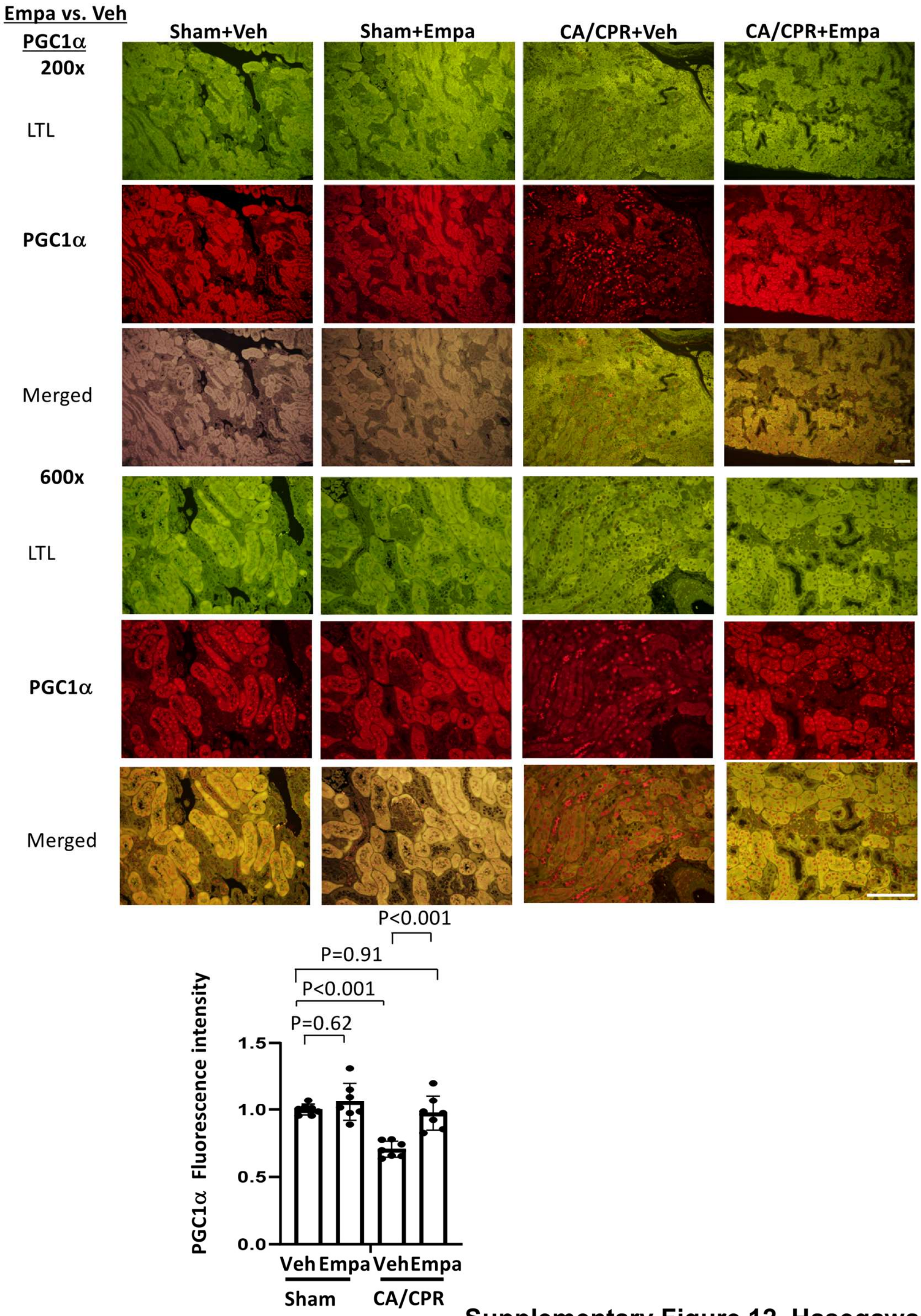

Supplementary Figure 12, Hasegawa et al.

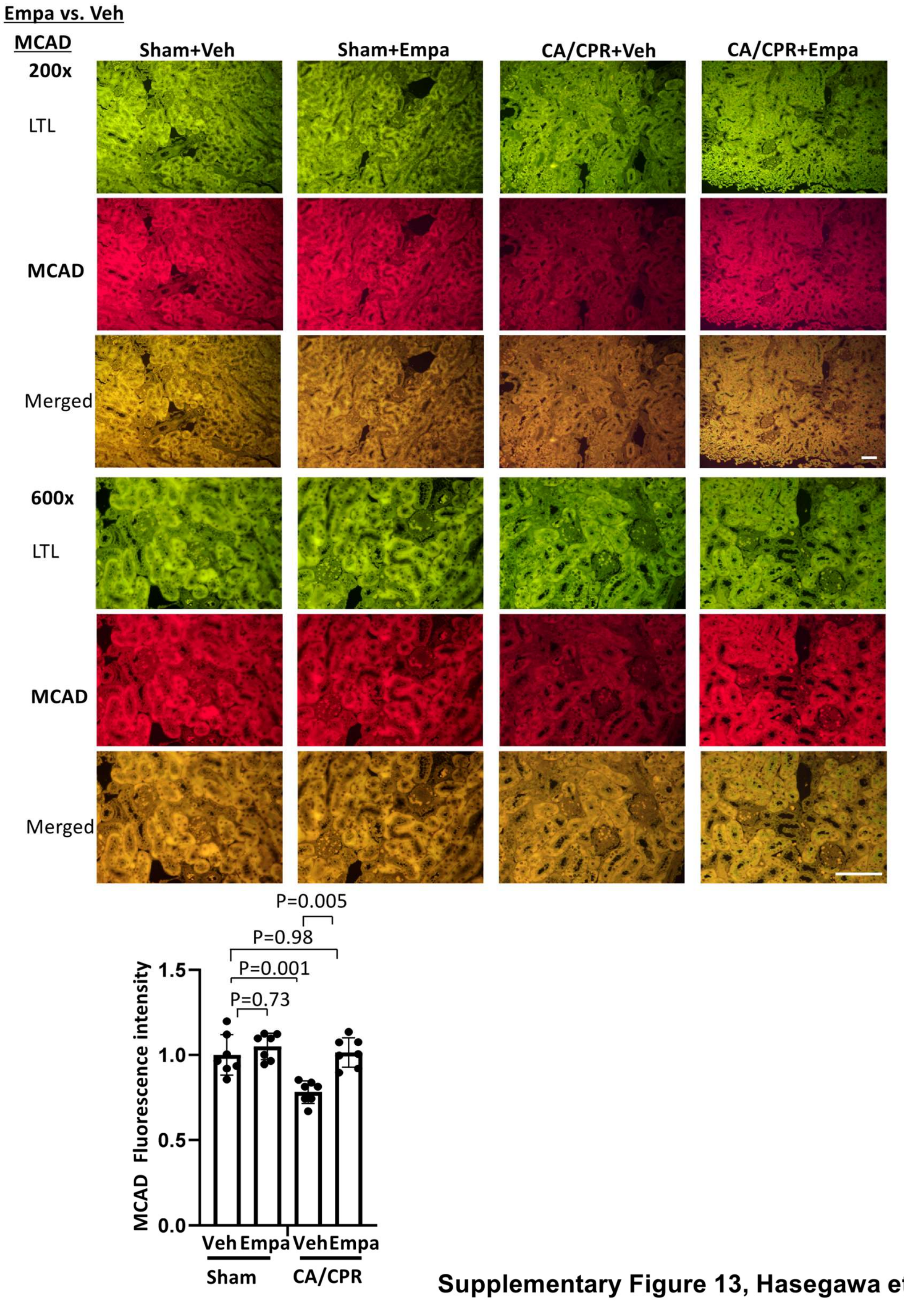

Supplementary Figure 13, Hasegawa et al.

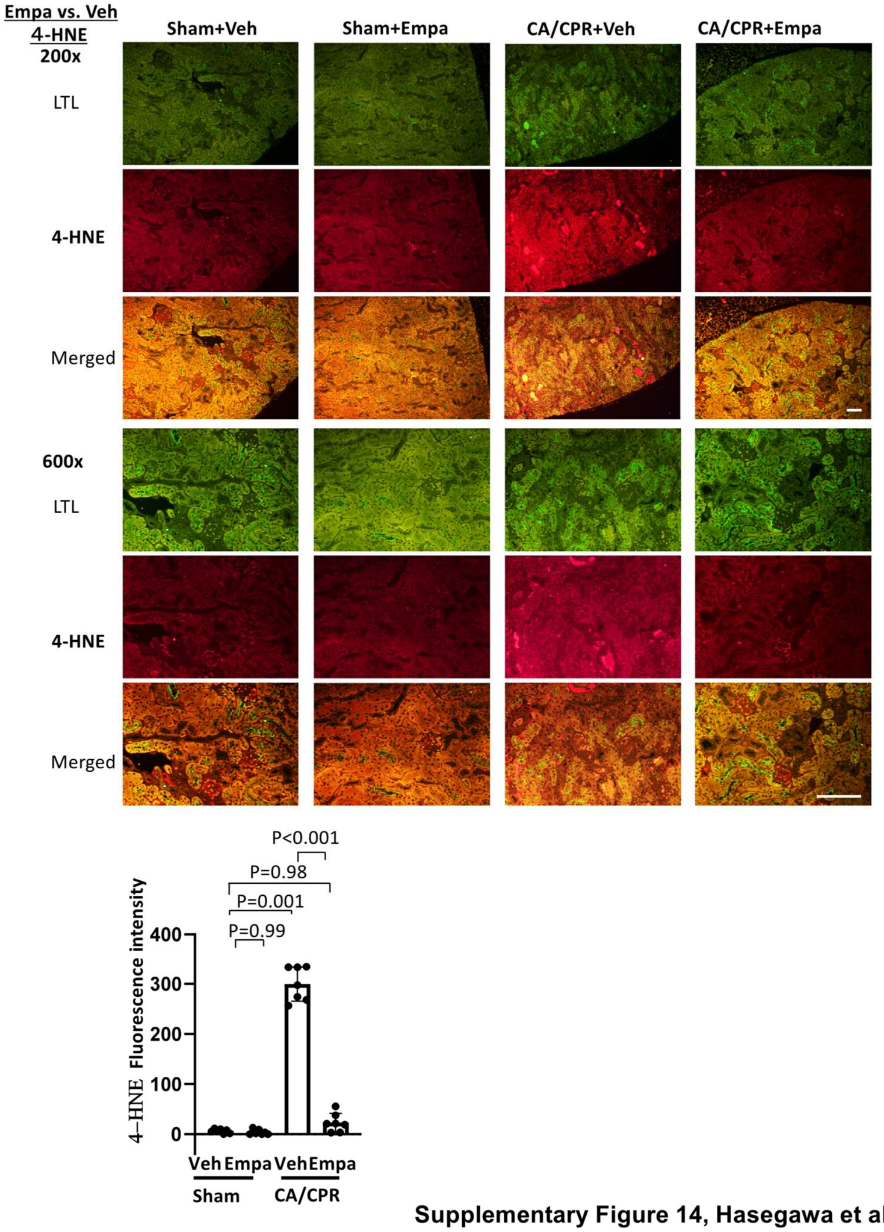

Supplementary Figure 14, Hasegawa et al.

Figures S12–S14. Mitochondrial markers and 4-HNE immunofluorescence in vehicle- and empagliflozin-treated mice subjected to CA/CPR. Low-magnification (200×) and high-magnification (600×) immunofluorescence images of kidney sections from Veh + Sham, Empa + Sham, Veh + CA/CPR, and Empa + CA/CPR mice at 9 weeks of age are shown. Kidney sections were stained with biotinylated LTL (green) as a proximal tubule marker, and mitochondrial markers were visualized in red. Supplementary Figure 12 shows PGC-1α, a marker of mitochondrial abundance; Supplementary Figure 13 shows medium-chain acyl-CoA dehydrogenase (MCAD), a marker of mitochondrial fatty acid oxidation; and Supplementary Figure 14 shows 4-hydroxynonenal (4-HNE), a marker of local ROS production. Veh + CA/CPR mice exhibited reduced mitochondrial abundance and function accompanied by increased ROS generation, whereas Empa + CA/CPR mice showed preserved or restored PGC-1α and MCAD expression together with markedly reduced 4-HNE staining. Representative images and quantitative fluorescence intensity analyses (mean ± SEM) are shown (n = 7 mice/group; scale bar: 50 μm). Statistical analyses were performed using a two-tailed Student's t-test.

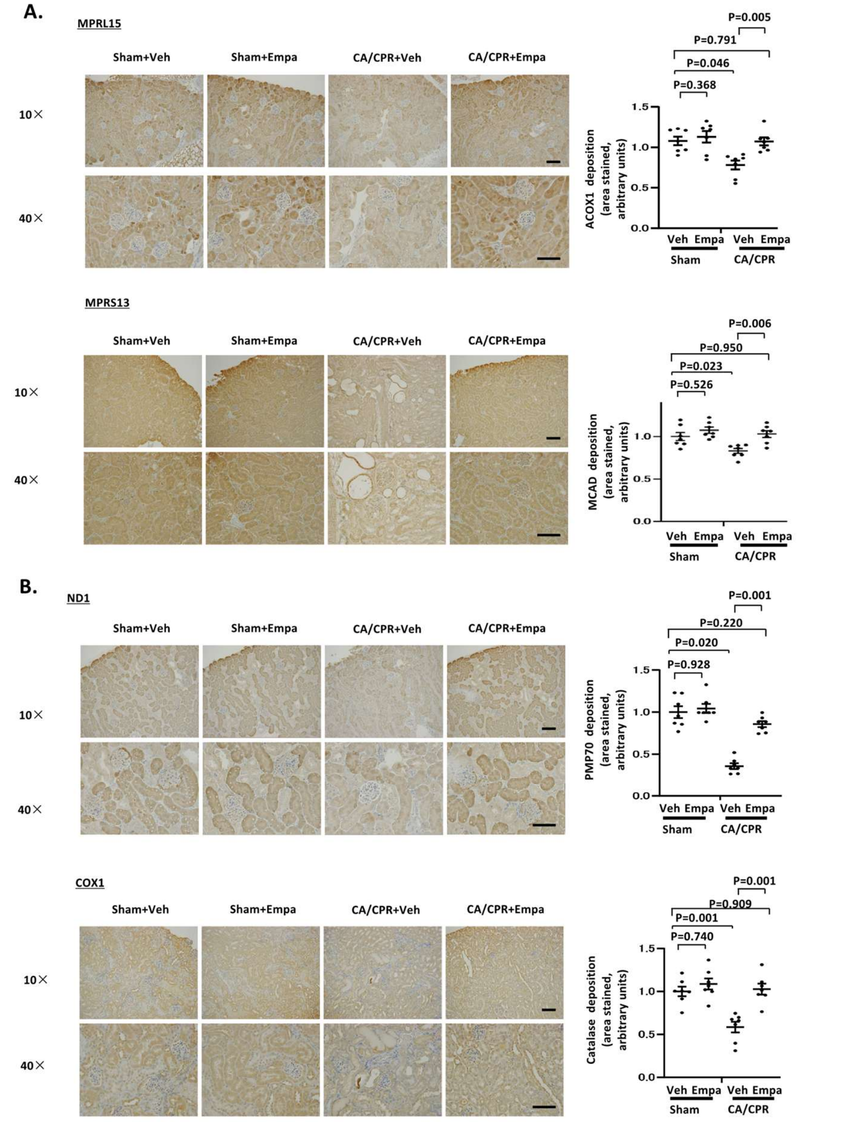

Supplementary Figure 15, Hasegawa et al.

Figure S15. Empagliflozin preserves mitoribosome abundance and function in mice subjected to CA/CPR. (A) Immunohistochemical staining for the mitoribosomal proteins MRPL13 (mitochondrial ribosomal protein L13) and MRPS15 (mitochondrial ribosomal protein S15) was performed in kidney sections from Veh + Sham, Empa + Sham, Veh + CA/CPR, and Empa + CA/CPR mice at 9 weeks of age. Both low-magnification (10×) and high-magnification (40×) images are shown. MRPL13 and MRPS15 represent large- and small-subunit mitoribosomal proteins, respectively, and together serve as markers of mitoribosome abundance. Veh + CA/CPR mice exhibited markedly reduced MRPL13 and MRPS15 staining, whereas Empa + CA/CPR mice showed preserved or restored expression. Representative images and quantitative immunohistochemical scoring (mean ± SEM) are shown (n = 7 mice/group; scale bar: 50 μm). (B) To evaluate mitoribosomal function, immunostaining for ND1 and COX1, mtDNA-encoded OXPHOS subunits translated exclusively by mitoribosomes, was performed. Low-magnification (10×) and high-magnification (40×) images are shown. Veh + CA/CPR mice exhibited significantly reduced ND1 and COX1 expression, indicating impaired mitoribosomal translation, whereas Empa + CA/CPR mice demonstrated recovery of these mtDNA-encoded OXPHOS proteins. Quantitative analyses are presented as mean ± SEM (n = 7 mice/group). Statistical analyses were performed using a two-tailed Student's t-test.

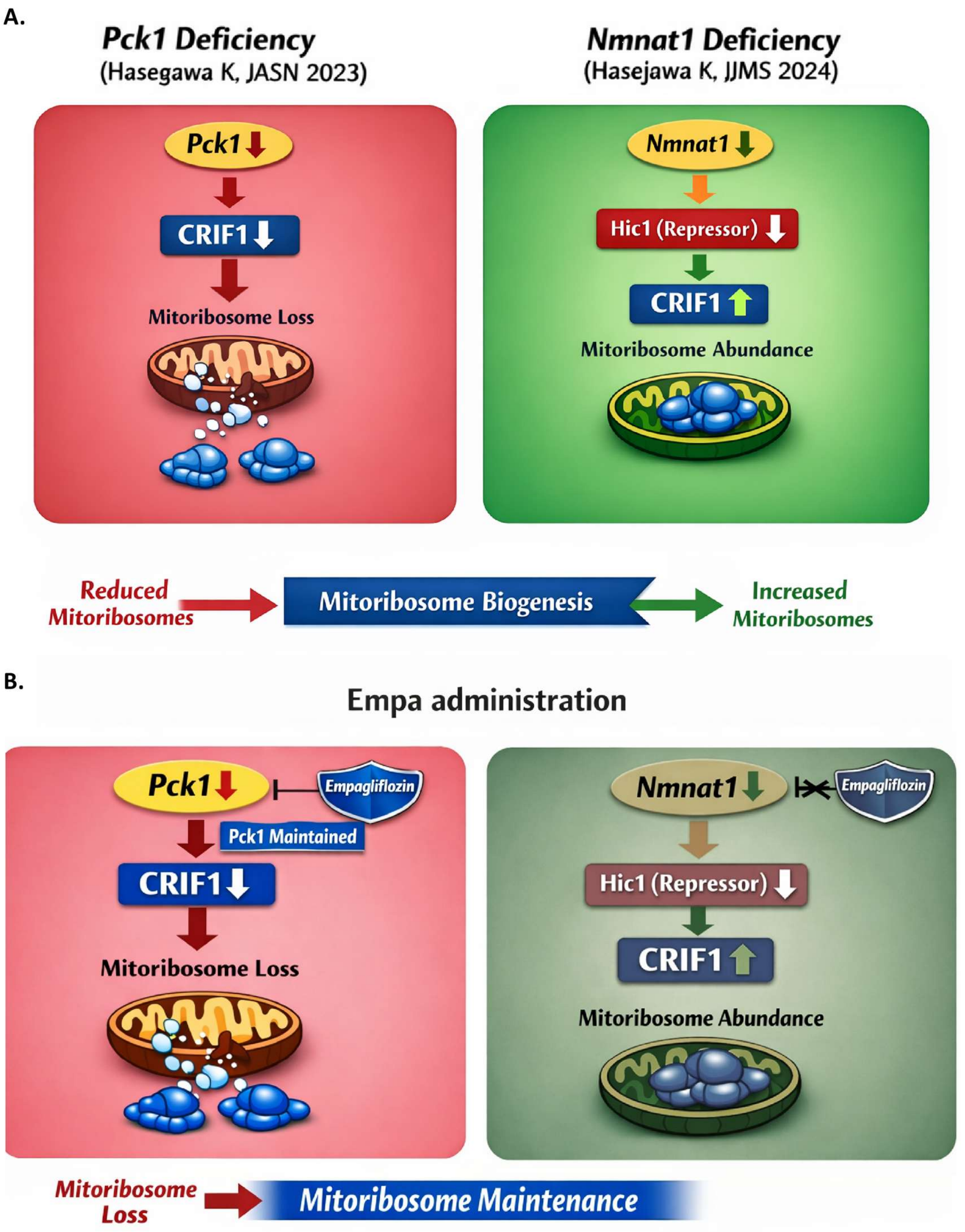

Supplementary Figure 16, Hasegawa et al.

Figure 16. Empagliflozin modulates mitoribosome maintenance under Pck1 and Nmnat1 deficiency. Schematic representation of the molecular pathways linking Pck1 and Nmnat1 deficiency to mitoribosome biogenesis and maintenance. In the upper panels, Pck1 deficiency (Hasegawa K, JASN 2023) reduces CRIF1 expression, leading to mitoribosome loss, whereas Nmnat1 deficiency (Hasegawa K, JJMS 2024) decreases Hic1 expression and increases CRIF1 expression, resulting in mitoribosome accumulation. In the lower panels, empagliflozin (Empa) maintains Pck1 expression but does not restore CRIF1, resulting in persistent mitoribosome depletion. In contrast, Empa does not affect Nmnat1 signaling; therefore, Hic1 remains suppressed and CRIF1 remains elevated, thereby preserving mitoribosome abundance.

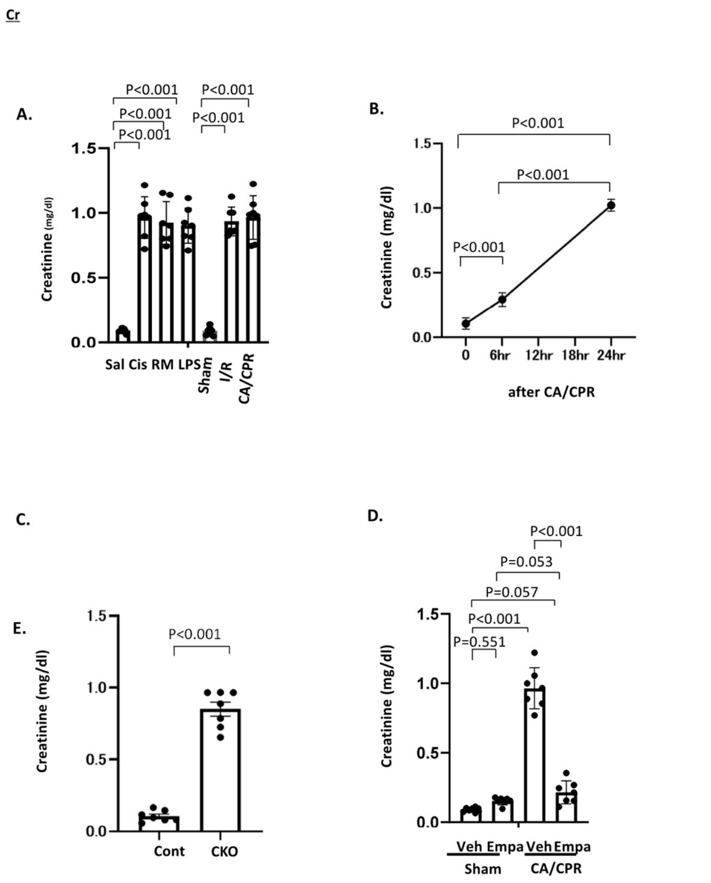

Supplementary Figure 17, Hasegawa et al.

Figure S17. Serum creatinine levels in multiple AKI models, the CA/CPR time course, Pck1 CKO mice, and empagliflozin-treated mice. (A) Serum creatinine levels in multiple AKI models. Acute kidney injury was induced in 9-week-old male C57BL/6 mice using cisplatin (Cis), rhabdomyolysis (RM), endotoxemia (LPS), ischemia–reperfusion (I/R), or cardiac arrest/cardiopulmonary resuscitation (CA/CPR). Controls received saline (Cis, RM, and LPS) or sham surgery (I/R and CA/CPR). Blood was collected 24 h after injury (n = 7 mice/group). All AKI models except saline and sham controls showed significant elevations in serum creatinine, indicating comparable AKI severity. Data were analyzed using one-way ANOVA followed by Tukey's post hoc test. Exact p-values are indicated in the figure. (B) Time course of serum creatinine after CA/CPR. Serum creatinine was measured at 0, 6, 12, 18, and 24 h after CA/CPR (n = 3 mice/time point). Creatinine levels increased progressively over time, confirming time-dependent worsening of renal dysfunction after CA/CPR. Data were analyzed using one-way ANOVA. Exact p-values are indicated in the figure. (C) Serum creatinine levels in proximal tubule-specific Pck1 CKO mice after short-time CA/CPR. Control (Cont) and Pck1 conditional knockout (CKO) mice were subjected to short-time CA/CPR (3-min arrest) at 9 weeks of age (n = 7 mice/group). CKO mice exhibited significantly higher serum creatinine levels than Cont mice, indicating increased susceptibility to CA/CPR-induced AKI. Data were analyzed using a two-tailed Student's t-test. Exact p-values are indicated in the figure. (D) Serum creatinine levels in Veh + Sham, Empa + Sham, Veh + CA/CPR, and Empa + CA/CPR mice. Empagliflozin (10 mg/kg/day) or vehicle was administered for 7 days before sham or CA/CPR procedures (n = 7 mice/group). CA/CPR markedly increased serum creatinine levels in vehicle-treated mice, whereas empagliflozin significantly attenuated this increase. Data were analyzed using two-way ANOVA followed by Tukey's post hoc test. Exact p-values are indicated in the figure.

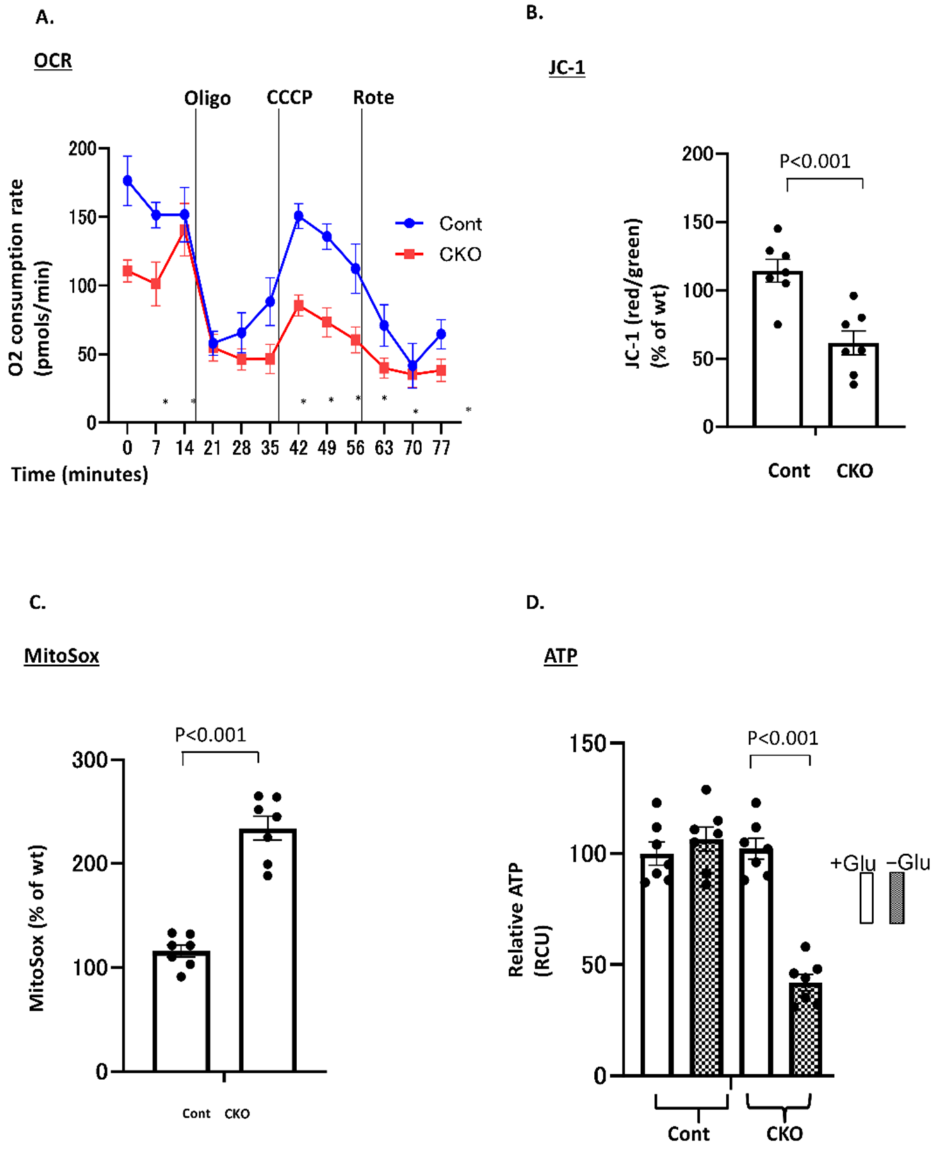

Supplementary Figure 18, Hasegawa et al.

Figure S18. Mitochondrial dysfunction in CKO mice. (A) The oxygen consumption rate (OCR) of TECs isolated from CKO and control mice was measured using a Seahorse XF-24 flux analyzer. N = 3. * $p < 0.05$. Cont, control; CKO, conditional knockout; OXPHOS, oxidative phosphorylation; CCCP, carbonyl cyanide m-chlorophenyl hydrazone. (B) The ratio of red/green fluorescence of JC-1 in TECs isolated from CKO and control mice was used as a measure of mitochondrial membrane potential. N = 7. (C) Fluorescence of MitoSox in TECs isolated from CKO and control mice as a measure of mitochondrial levels of reactive oxygen species. N = 7. (D) ATP content in TECs isolated from CKO and control mice. N = 7. All data are presented as mean ± SEM. Data were analyzed using a two-tailed Student's *t*-test. Exact *p*-values are indicated in the figures.

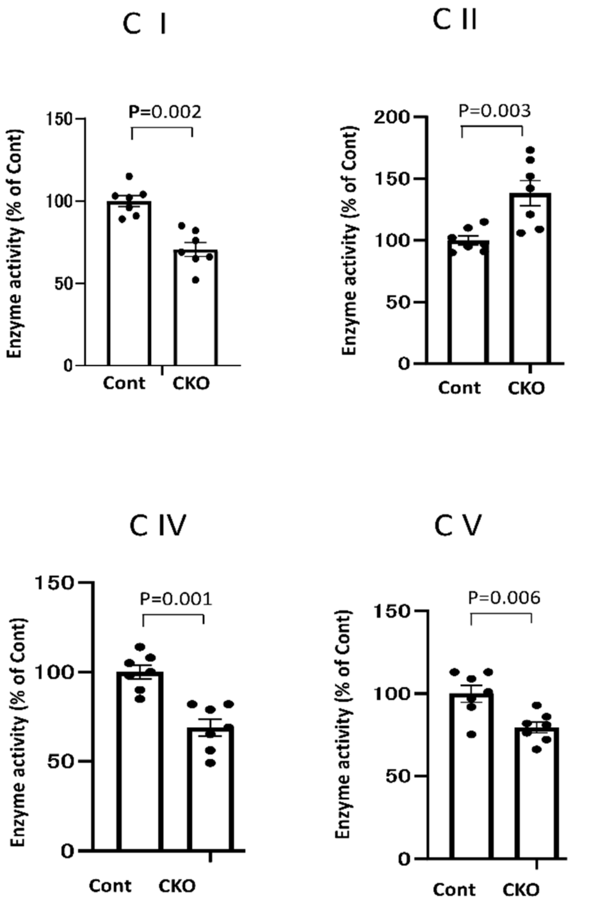

Supplementary Figure 19, Hasegawa et al.

Figure S19. Measurement of mitochondrial electron transport chain (ETC) complex enzyme activities in the kidneys of CKO mice. CII-related genes and proteins were only coded by nuclear DNA (nDNA) and cytoplasmic ribosomes (cytoribosomes), which were not influenced by mitochondrial DNA (mtDNA) and mitoribosomes. Conversely, mtDNA encodes and mitoribosomes synthesize CI-, CIII-, CIV-, CV-related genes and proteins, some of which were also encoded by nDNA and synthesized by cytoribosomes. Thus, mitoribosomes synthesize CI-, CIII-, CIV-, and CV-related component proteins but not CII-related proteins. Complex activities were analyzed as described in the Methods section. All activity levels were calculated as the average of seven assays from each group of mice. Citrate synthase activity was used to normalize the level of mitochondrial proteins. Activities of CI–CV are plotted. All data are shown as the mean ± standard error of the mean (SEM). The kidney tissue specimens were obtained from CKO and control mice. Mitoribosomal dysfunction decreased CI, CIII, CIV, and CV activity, which led to a reduction in oxygen consumption rate (OCR). Elevated CII activity might compensate for the reduction in CI, CIII, CIV, and CV activities. Data were analyzed using a two-tailed Student's *t*-test. Exact *p*-values are indicated in the figures.

A.

Catalase activity

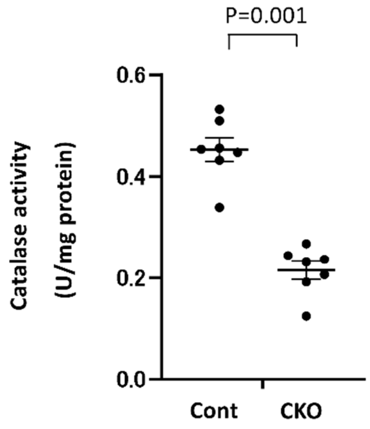

B.

ACOX1 activity

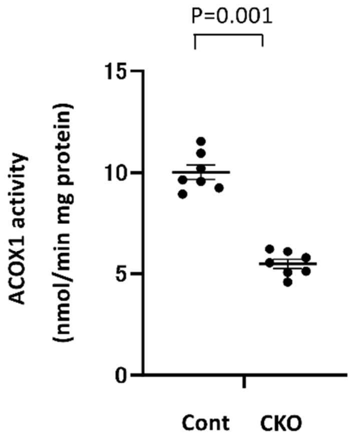

Supplementary Figure 20, Hasegawa et al.

Figure S20. Catalase and ACOX1 activity assays in conditional KO mice. Enzyme activities in conditional KO mice. Catalase (A) and ACOX1 (B) activities were assessed using kidney tissues from CKO and Cont mice. N = 7 mice per group. Data were assessed using two-tailed Student's t-test.

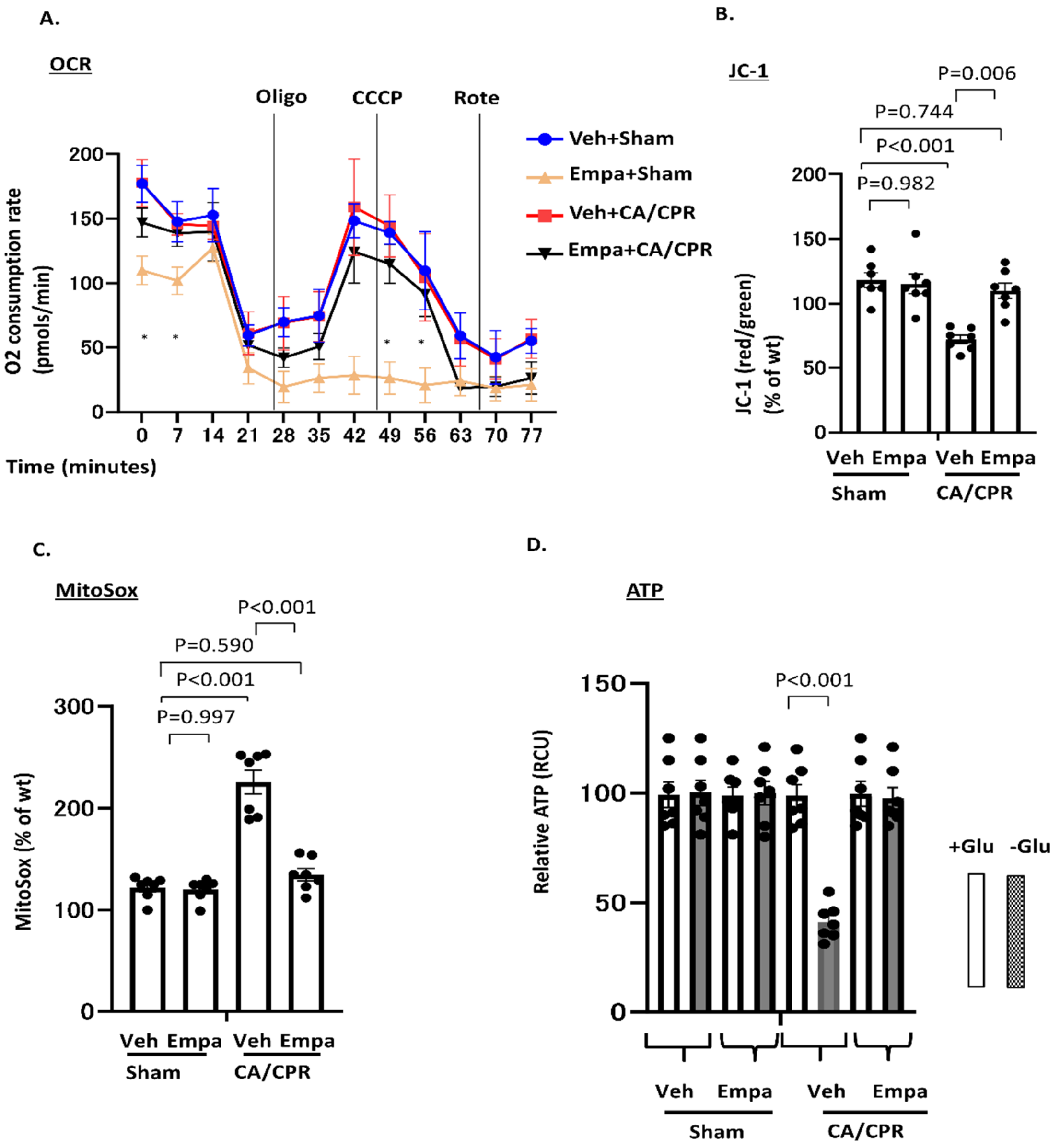

Supplementary Figure 21, Hasegawa et al.

Figure S21. Mitochondrial protection in empagliflozin-treated mice. (A) The OCR of tubular epithelial cells (TECs) isolated from each group of mice was measured using a Seahorse XF-24 flux analyzer. N = 3. *$p < 0.05$. (B) The ratio of red/green fluorescence of JC-1 of TECs isolated from Veh + Sham, Empa + Sham, Veh + CA/CPR, and Empa + CA/CPR mice at 9 weeks of age as a measure of the mitochondrial membrane potential. N = 6. (C) Fluorescence of MitoSox of TECs isolated from each mouse group as a measure of mitochondrial levels of reactive oxygen species. N = 7. (D) The ATP content of TECs isolated from each mouse group. N = 7. All data are presented as mean ± standard errors of the mean. Quantitative data were analyzed using one-way ANOVA followed by Bonferroni correction (n = 7 mice/group). Exact *p*-values are indicated in the figures.

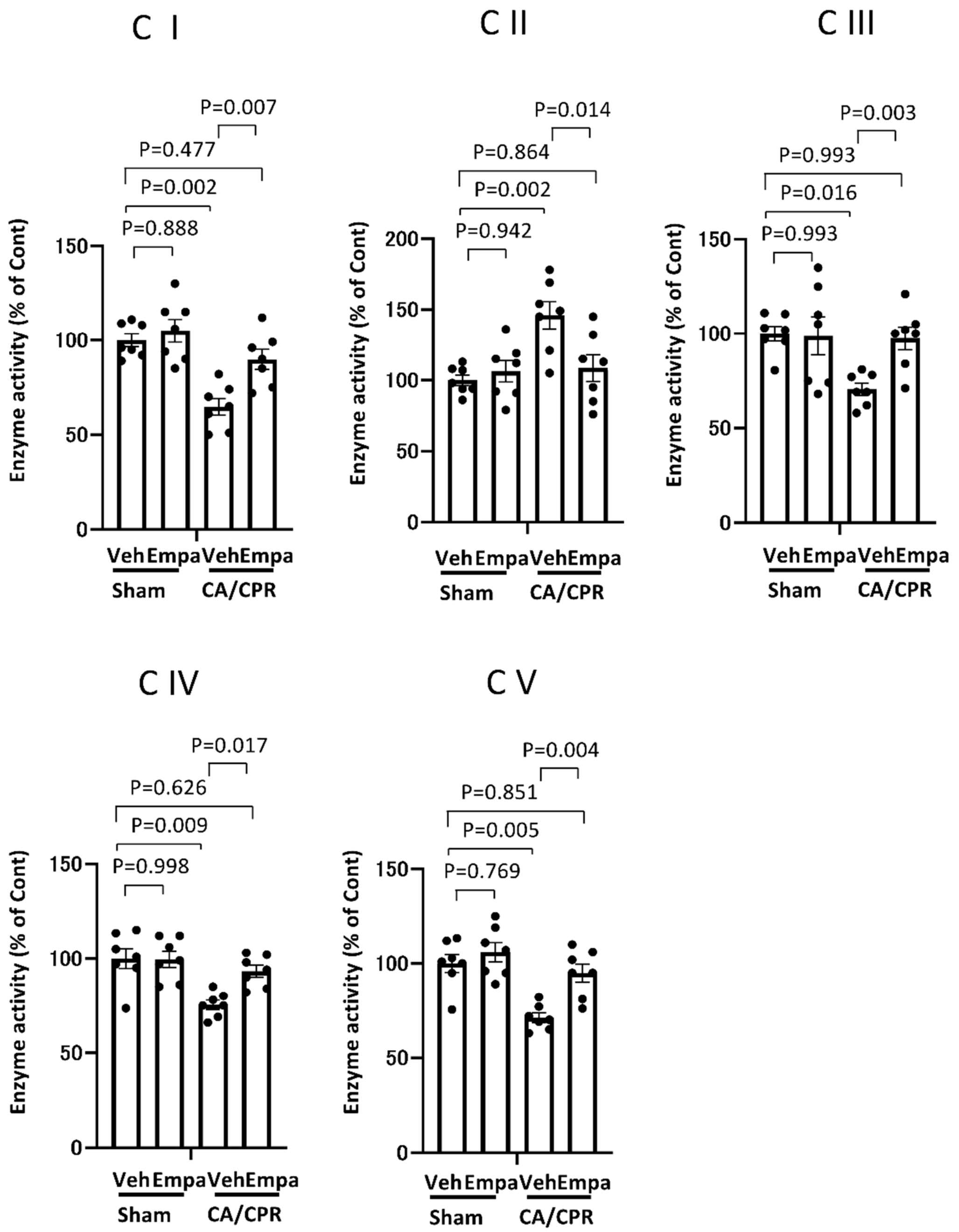

Supplementary Figure 22, Hasegawa et al.

Figure S22. Measurement of mitochondrial ETC complex enzyme activities in empagliflozin-treated mice. All activity findings are the average of 7 assays from the pooled samples of each group of mice at 9 weeks of age. Citrate synthase activities were used to normalize mitochondrial proteins. Activities for CI-CV are plotted. All data are shown as the mean ± standard error of the mean. Quantitative data were analyzed using one-way ANOVA followed by Bonferroni correction (n = 7 mice/group). Exact p-values are indicated in the figures.

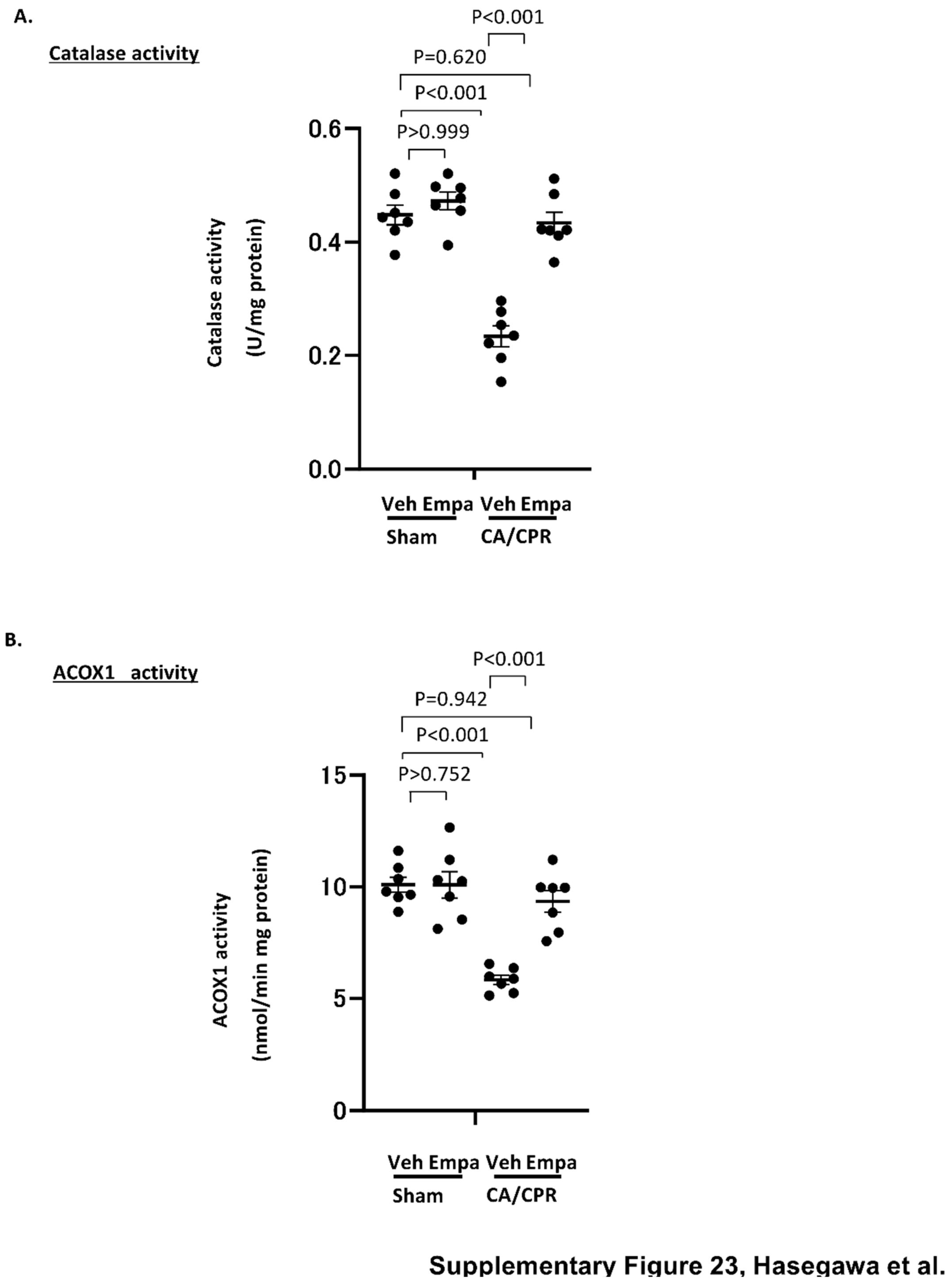

Figure S23. Catalase and ACOX1 activity assays in in empagliflozin-treated mice.Enzyme activities in each group of mice at 9 weeks of age. Catalase (A) and ACOX1 (B) activities were assessed using kidney tissues. N = 7 mice per group. Quantitative data were analyzed using one-way ANOVA followed by Bonferroni correction (n = 7 mice/group). Exact p-values are indicated in the figures.

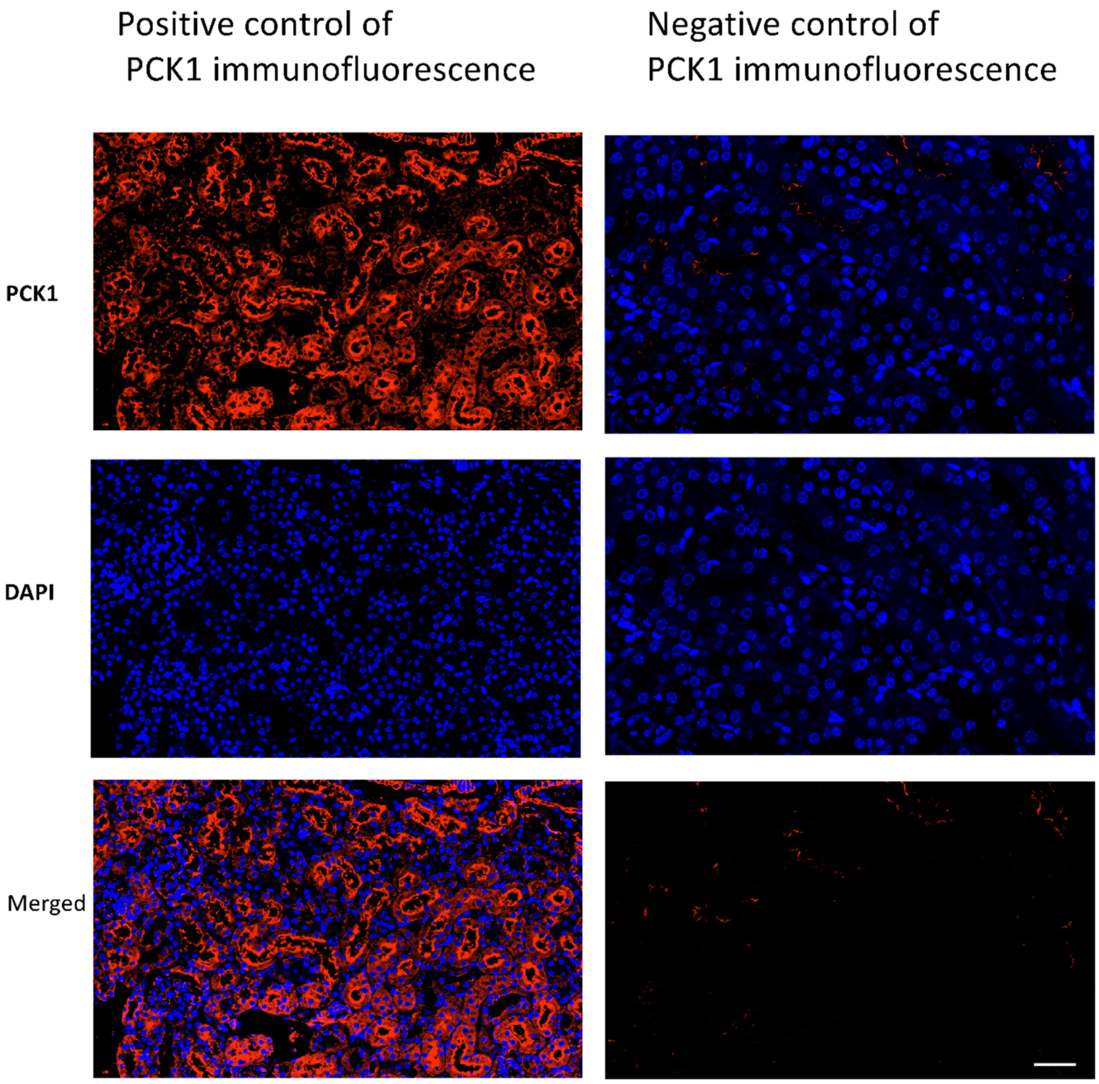

Supplementary Figure 24, Hasegawa et al.

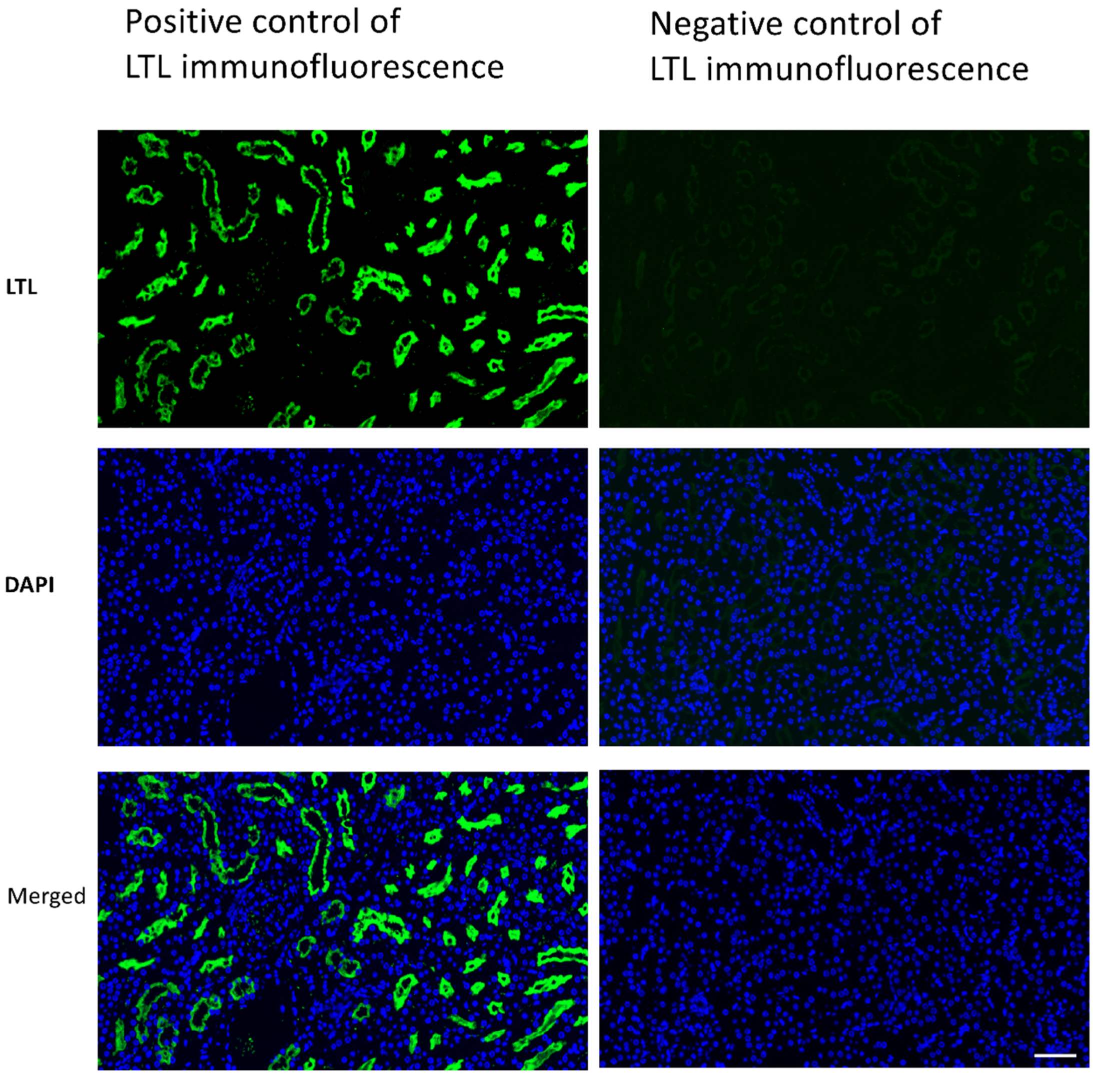

Supplementary Figure 25, Hasegawa et al.

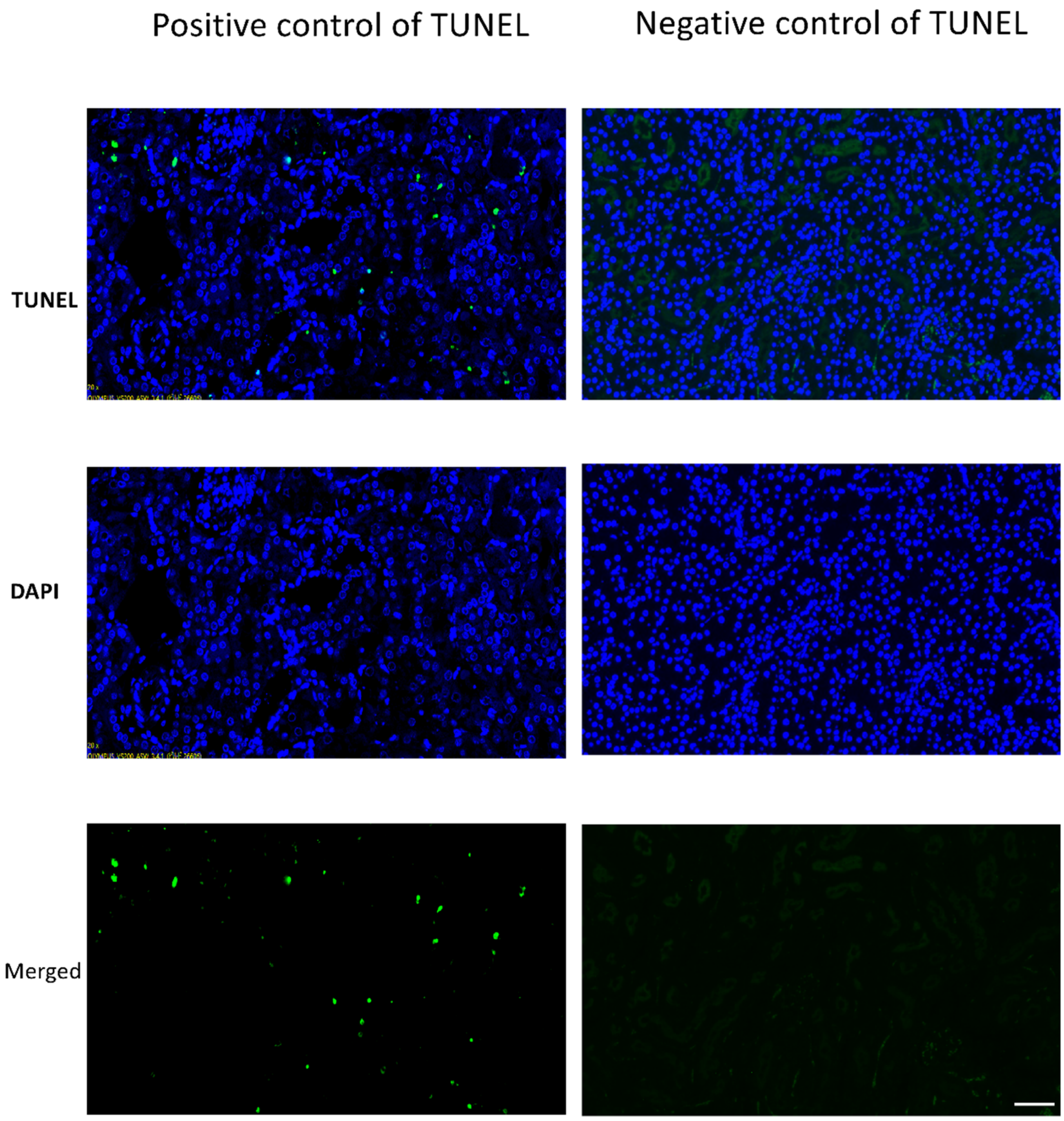

Supplementary Figure 26, Hasegawa et al.

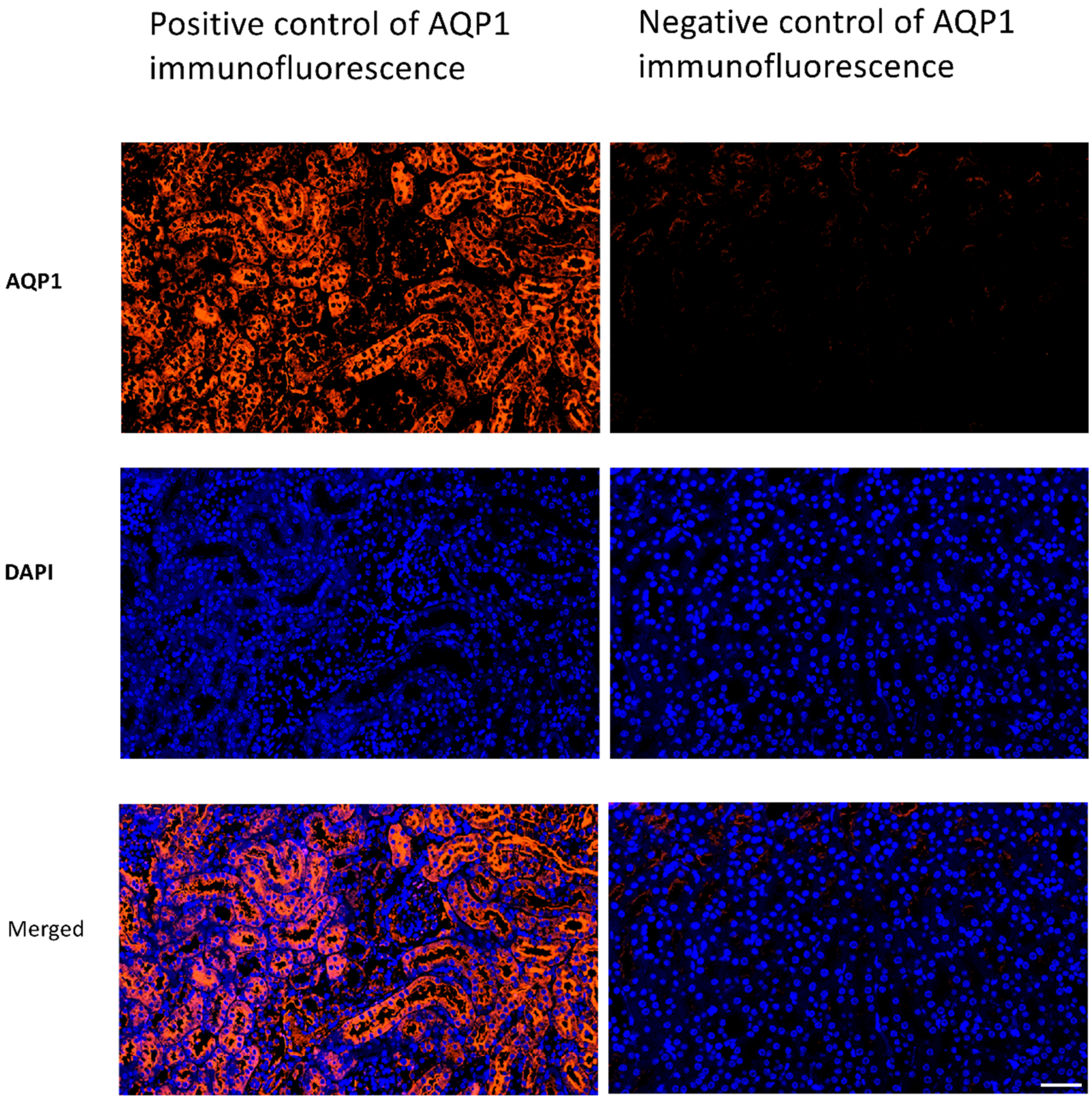

Supplementary Figure 27, Hasegawa et al.

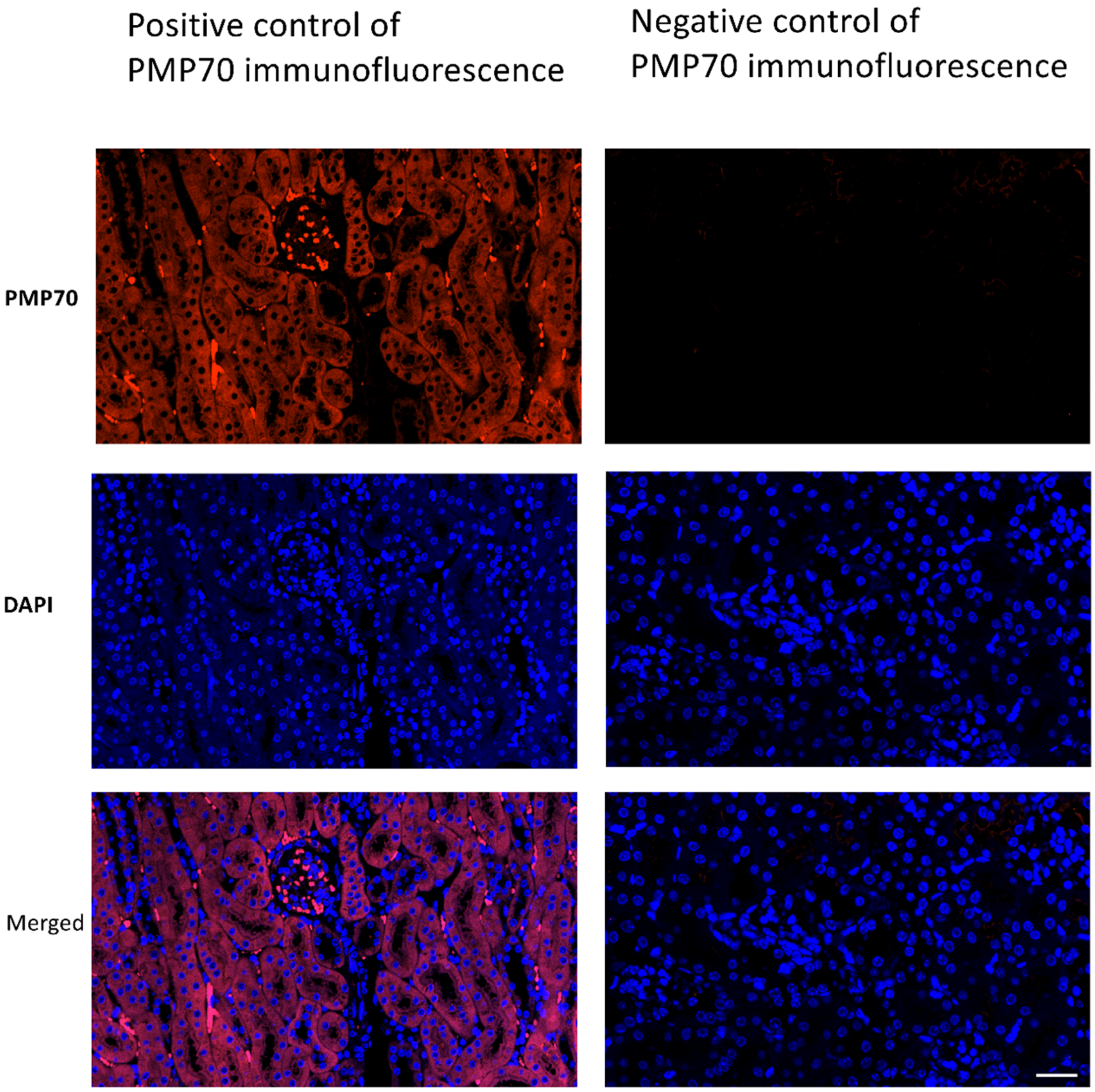

Supplementary Figure 28, Hasegawa et al.

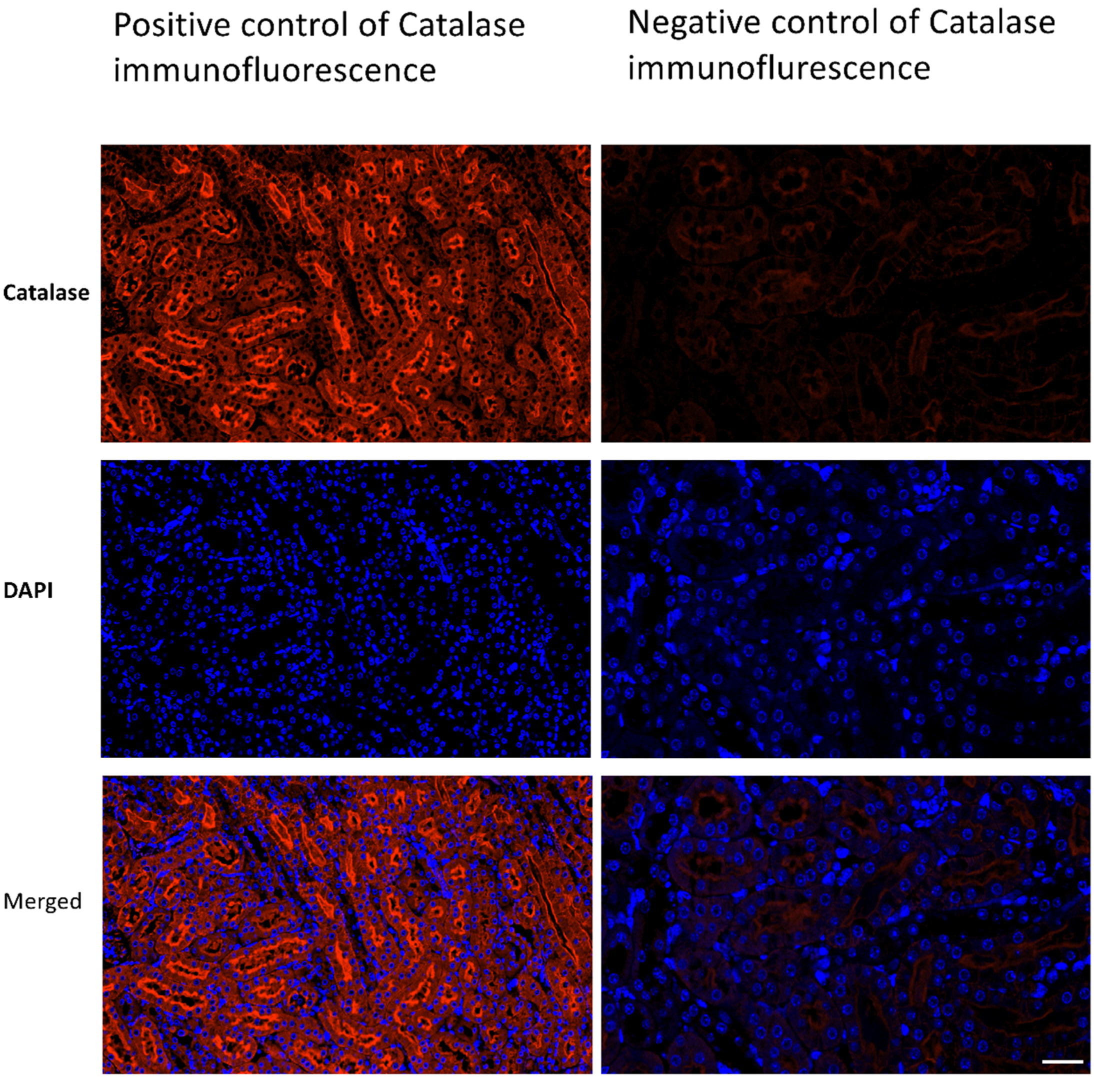

Supplementary Figure 29, Hasegawa et al.

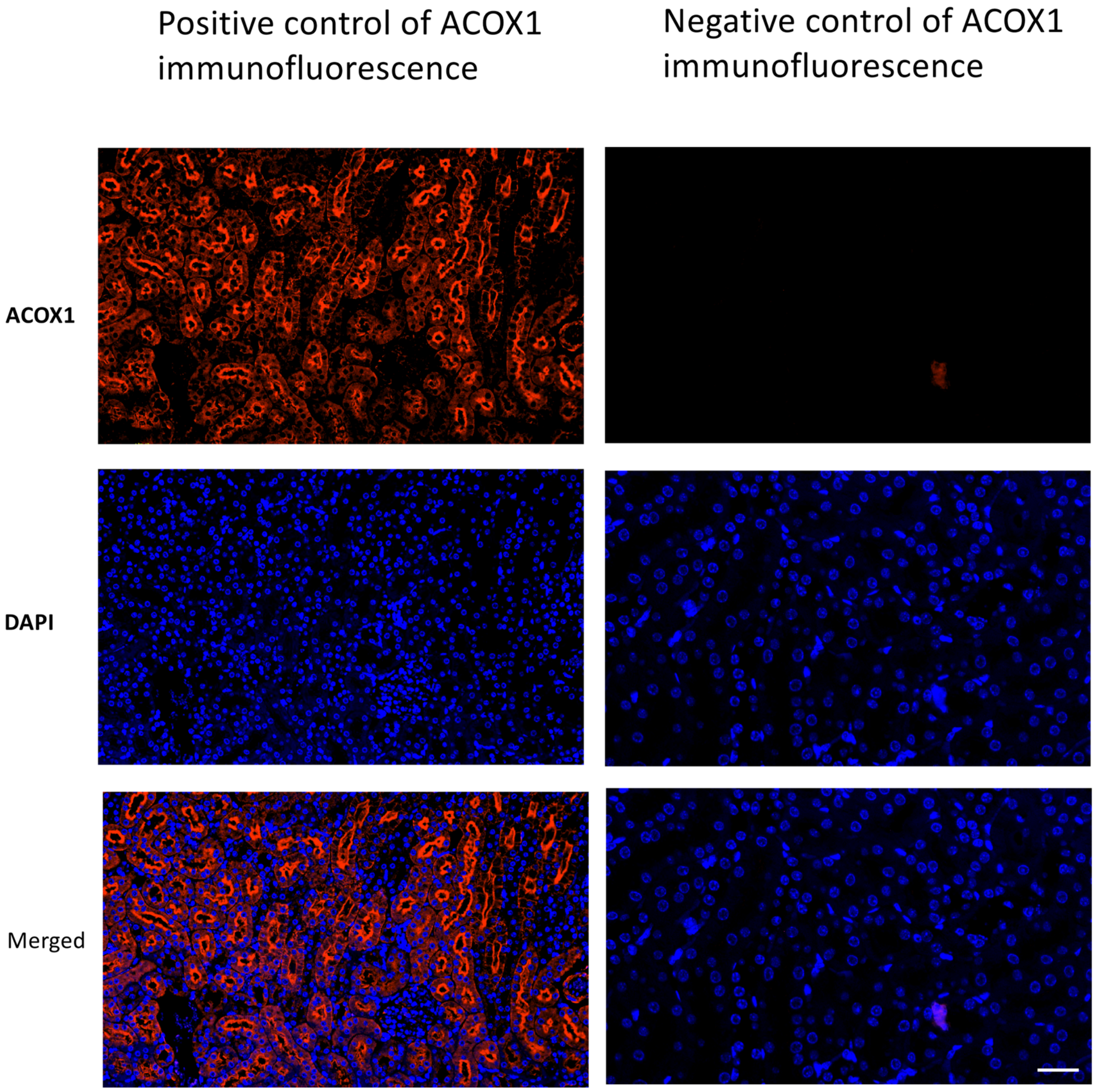

Supplementary Figure 30, Hasegawa et al.

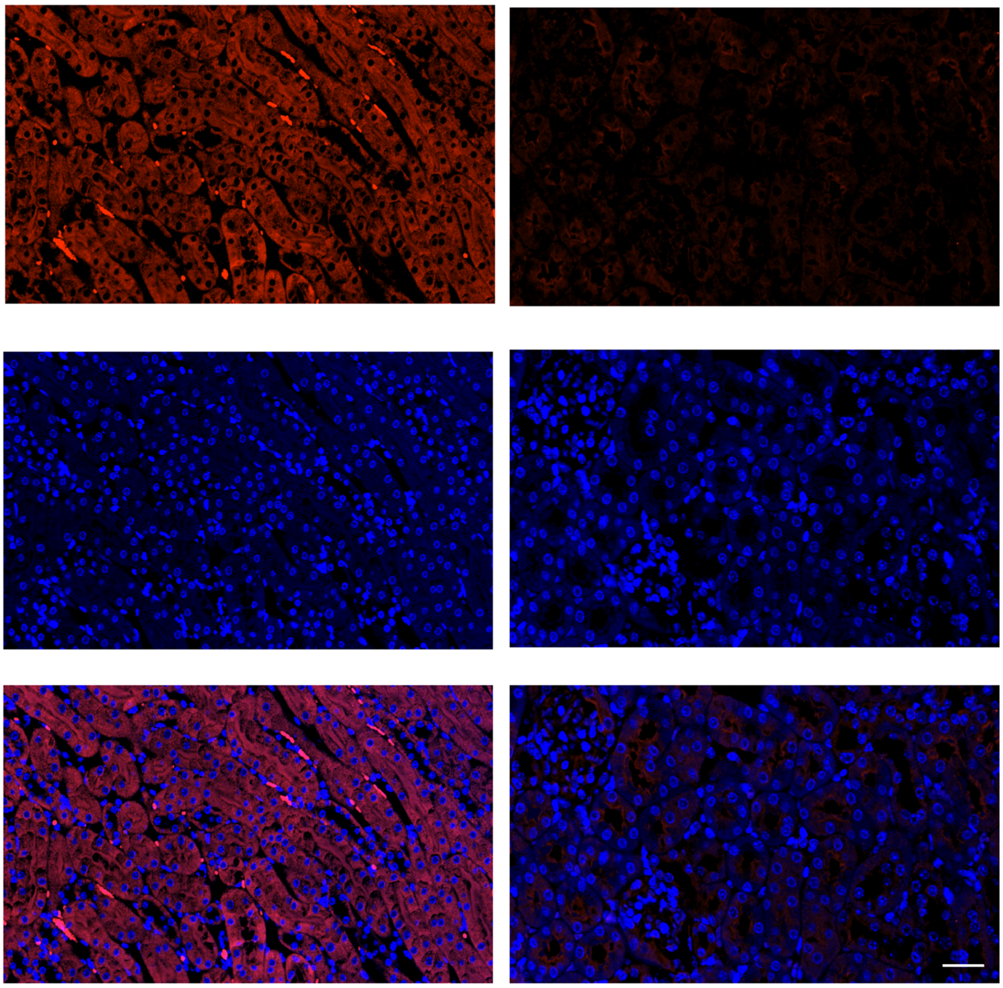

Supplementary Figure 31, Hasegawa et al.

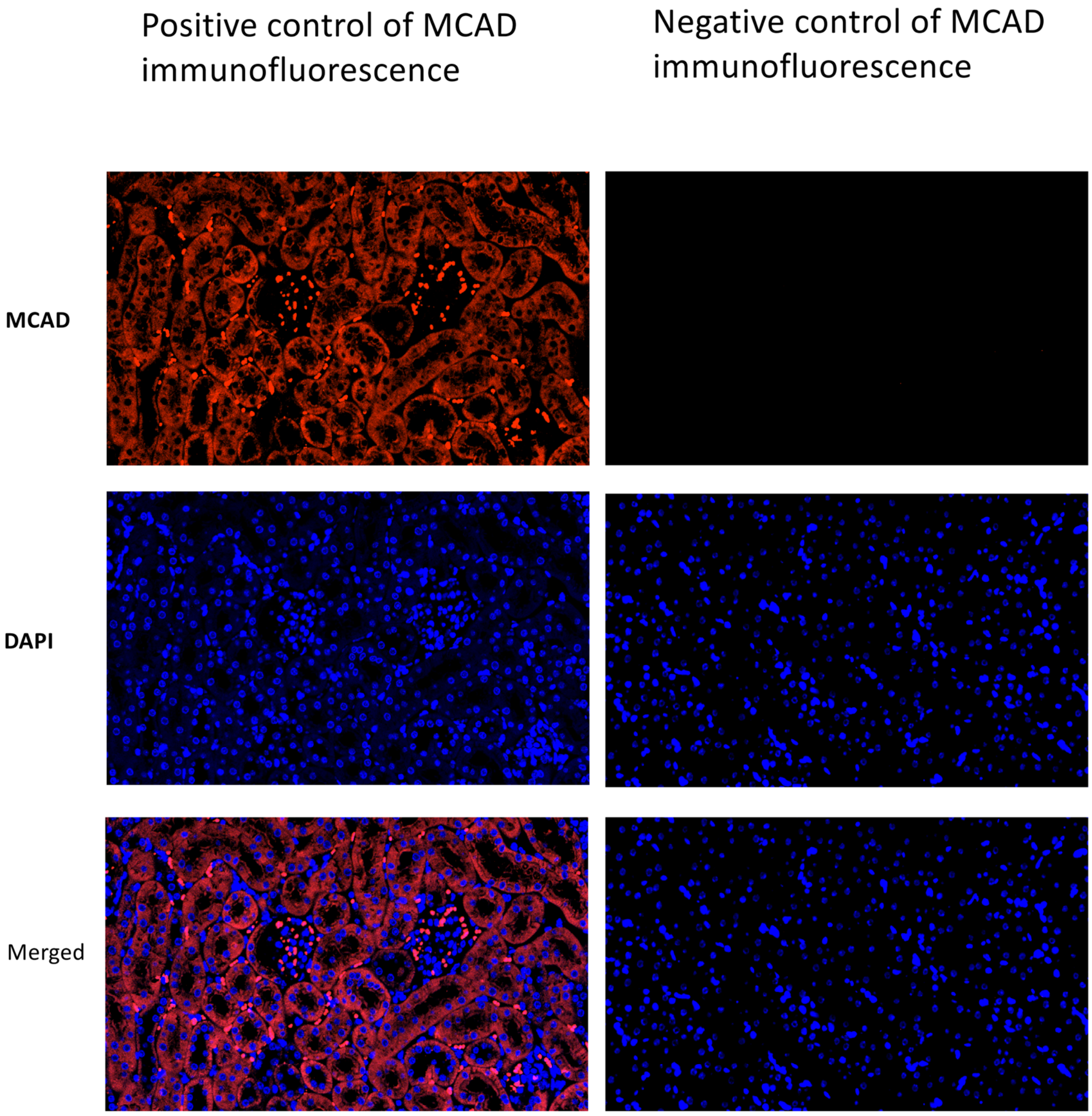

Supplementary Figure 32, Hasegawa et al.

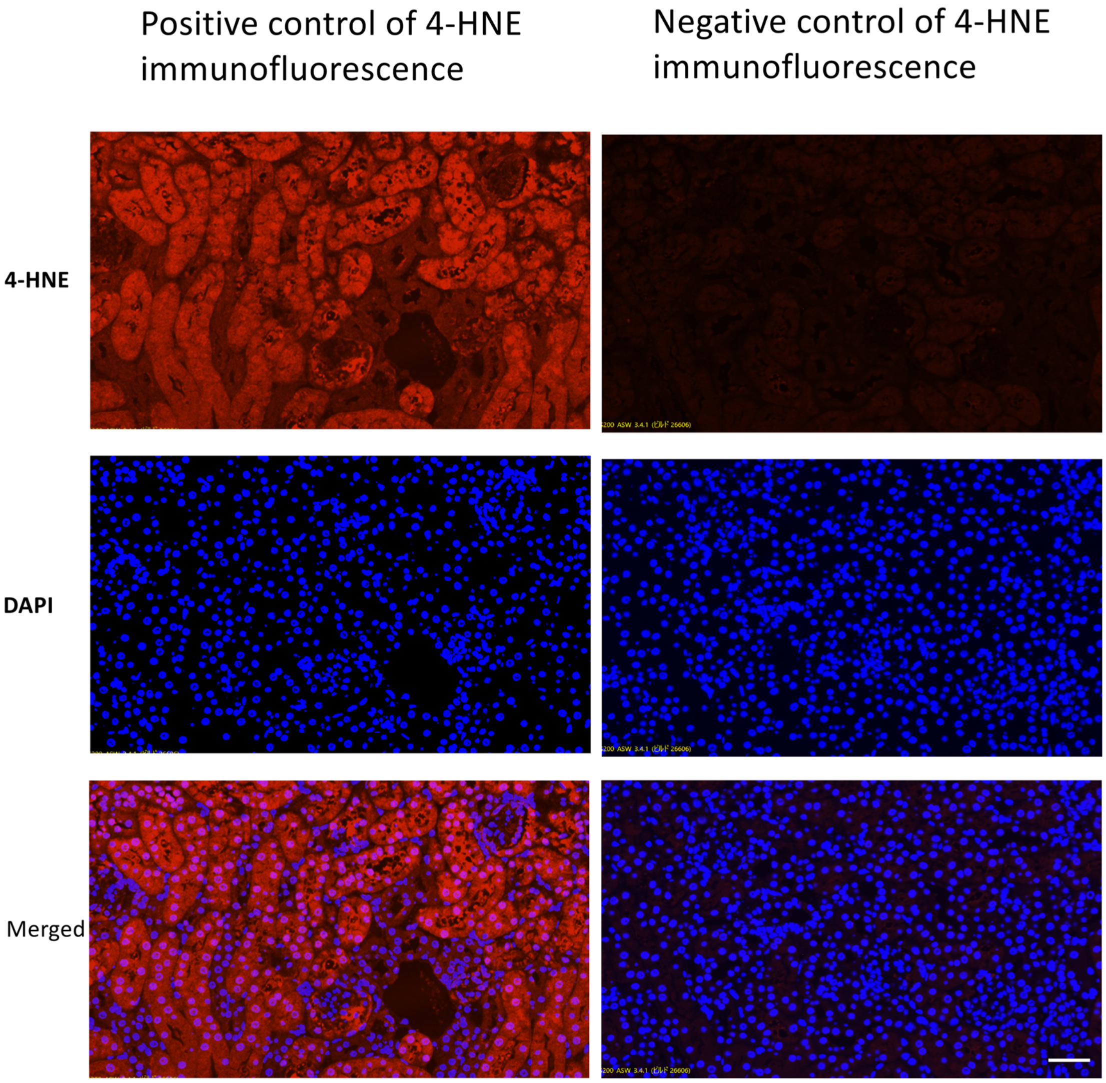

Supplementary Figure 33, Hasegawa et al.

Figure S24–33 Positive and negative controls of mouse kidney were used for immunofluorescence staining for LTL (S-Fig. 24), PCK1 (S-Fig. 25), TUNEL (S-Fig. 26), AQP-1 (S-Fig. 27), PMP70 (S-Fig. 28), ACOX1 (S-Fig. 29), catalase (S-Fig. 30), PGC1a (S-Fig. 31), MCAD (S-Fig. 32), and 4-HNE (S-Fig. 33). Kidneys from 9-week-old healthy wild-type mouse were used as positive control. For 4-HNE immunofluorescence, a 15-month-old aging mouse kidney was used. For the negative control of each immunofluorescence, primary antibodies preblocked with immunogen solution (i.e., immune depletion) were used. DAPI staining of nuclei are shown in blue. LTL immunofluorescence and TUNEL signals are shown in green. PCK1, AQP-1, PMP70, ACOX1, catalase, PGC1a, MCAD, and 4-HNE immunofluorescence are shown in red. Scale bars represent 50 μm. In the TUNEL negative control assay (S-Fig. 26), removal of the TdT enzyme from the TdT reaction buffer did not display TUNEL-positive cells.
